# How the Interplay among Conformational Disorder, Solvation, Local, and Charge-Transfer Excitations Affects the Absorption Spectrum and Photoinduced Dynamics of Perylene Diimide Dimers: A Molecular Dynamics/Quantum Vibronic Approach

**DOI:** 10.1021/acs.jctc.2c00063

**Published:** 2022-04-04

**Authors:** Alekos Segalina, Daniel Aranda, James A. Green, Vito Cristino, Stefano Caramori, Giacomo Prampolini, Mariachiara Pastore, Fabrizio Santoro

**Affiliations:** † Université de Lorraine and CNRS, LPCT, UMR 7019, F-54000 Nancy, France; ‡ Instituto de Ciencia Molecular (ICMol), Universidad de Valencia, Catedrático J. Beltrán 2, 46980 Paterna, Valencia, Spain; § Consiglio Nazionale delle Ricerche, Istituto di Biostrutture e Bioimmagini (IBB-CNR), via Mezzocannone 16, I-80136 Napoli, Italy; ∥ Dipartimento di Scienze Chimiche, Farmaceutiche ed Agrarie, Via Fossato di Mortara 17, 44121 Ferrara, Italy; ⊥ Istituto di Chimica dei Composti Organo Metallici, Consiglio Nazionale delle Ricerche, (ICCOM-CNR), SS di Pisa, Area della Ricerca, via G. Moruzzi 1, I-56124 Pisa, Italy

## Abstract

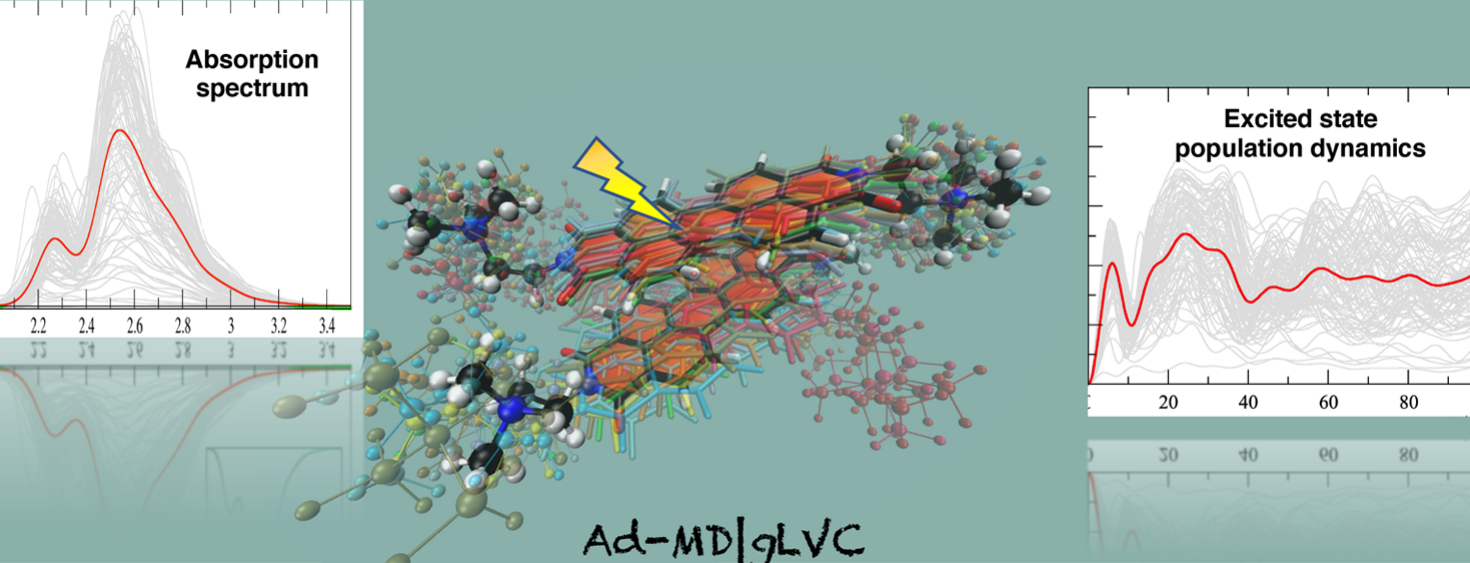

In this contribution we present a mixed quantum-classical dynamical approach for the computation of vibronic absorption spectra of molecular aggregates and their nonadiabatic dynamics, taking into account the coupling between local excitations (LE) and charge-transfer (CT) states. The approach is based on an adiabatic (Ad) separation between the soft degrees of freedom (DoFs) of the system and the stiff vibrations, which are described by the quantum dynamics (QD) of wave packets (WPs) moving on the coupled potential energy surfaces (PESs) of the LE and CT states. These PESs are described with a linear vibronic coupling (LVC) Hamiltonian, parameterized by an overlap-based diabatization on the grounds of time-dependent density functional theory computations. The WPs time evolution is computed with the multiconfiguration time-dependent Hartree method, using effective modes defined through a hierarchical representation of the LVC Hamiltonian. The soft DoFs are sampled with classical molecular dynamics (MD), and the coupling between the slow and fast DoFs is included by recomputing the key parameters of the LVC Hamiltonians, specifically for each MD configuration. This method, named Ad-MD|gLVC, is applied to a perylene diimide (PDI) dimer in acetonitrile and water solutions, and it is shown to accurately reproduce the change in the vibronic features of the absorption spectrum upon aggregation. Moreover, the microscopic insight offered by the MD trajectories allows for a detailed understanding of the role played by the fluctuation of the aggregate structure on the shape of the vibronic spectrum and on the population of LE and CT states. The nonadiabatic QD predicts an extremely fast (∼50 fs) energy transfer between the two LEs. CT states have only a moderate effect on the absorption spectrum, despite the fact that after photoexcitation they are shown to acquire a fast and non-negligible population, highlighting their relevance in dictating the charge separation and transport in PDI-based optical devices.

## 1 Introduction

The peculiar and tunable optoelectronic properties of perylene diimide (PDI)-based dyes and their relatively low production costs have made them attractive for several technological applications, including electronic displays, solar cell devices, and phototheranostic drugs for cancer therapy.^1−5^ In addition to the possibility of tethering PDI monomers by using various linkers, it has been shown that PDIs may spontaneously self-assemble in various solutions, forming large-size aggregates.^6−10^ In the latter case, the self-assembly is driven by a delicate interplay of nonbonded hydrophilic, hydrophobic, and π-stacking interactions.^6−10^ Such an equilibrium is easily altered by modifying, for instance, the PDI substitution patterns at the imide position, hence effectively controlling the dimension and shape of the aggregates.^6^ The shape of the aggregates, in turn, remarkably impacts the material’s optoelectronic properties.^11−14^

From a theoretical point of view, a simple connection between the structure of the supramolecular aggregates and the optical properties is possible within the qualitative Kasha’s model: cofacial arrangement leading to blue-shifted absorption relative to the monomer (H aggregate) and head-to-tail arrangement leading to red-shifted absorption (J-aggregate).^15^ However, potentially significant contributions are neglected in Kasha’s model: (i) the electron-vibrational coupling; (ii) the overlap between the frontier orbitals of the chromophores; (iii) the effect of interstate couplings among low-energy quasi-degenerate states; (iv) the nonbonded electrostatic interactions among the chromophores and with the environmental solvent molecules; and (v) the dynamical effects. As a matter of fact, unlike inorganic crystalline or glassy materials, the optical excitations and charge transport in π-conjugated molecular aggregates involve significant rearrangements of the nuclei positions, yielding strong vibronic coupling between electronic excitations and intramolecular vibrations.^12,16−18^ Since the seminal works of Fulton and Gouterman,^19,20^ where the effect of the vibronic coupling in molecular dimers was first discussed, many later works investigated the impact of vibronic coupling on molecular aggregates, showing, in many cases, its fundamental role in obtaining a good agreement with experimental spectra.^18,21−36^

In this framework, the case of PDI is paradigmatic. The optical absorption spectra of both PDI monomer and aggregates actually exhibit an evident vibronic progression that has been connected to the coupling between the first bright electronic transition and the intramolecular totally symmetric vinyl stretching mode, which has an energy of ∼0.17 eV.^13,37^ The net difference between the aggregates’ spectrum and the one of the monomer stands not only in the energetic shift, as argued by Kasha, but also in the change of the relative intensity between the first two vibronic peaks (*R* = *I*_0→0_/*I*_0→1_), which is indeed used as a spectral signature to identify the formation of PDI aggregates.^10,12,13,38,39^ As evidenced by Spano and co-workers,^12,13^ a large *R* factor (*R* > 1) is observed in J-aggregate spectra, whereas a small value of *R* (*R* < 1) corresponds to the case of H aggregates. The magnitude of this difference depends on the extent (number of chromophores involved), the excitonic bandwidth, and the nature of the exciton (pure Frenkel, charge transfer (CT), or mixed Frenkel/CT).

Furthermore, several studies have claimed that a proper description of the absorption and emission properties of PDI aggregates requires taking into account the interstate coupling, e.g., between Frenkel-like and CT-like states that compose the low-energy quasi-degenerate excited-state manifold.^12,14,40−43^ In PDI aggregates, when the interstate coupling is large enough, in particular when the Frenkel-like and CT-like states are strongly mixed, the excited-state relaxation may lead to the formation of excimers, self-trapped excitons, and the population of CT states, which have a major role in dictating the emission properties and the exciton diffusion.^2,12,40,44−46^ Moreover, as far as aggregates absorption spectra in solution are concerned, these appear broadened by the structural thermal fluctuations of both the self-assembled monomers and the surrounding solvent molecules.^47−50^ Indeed, recent studies have shown that the contribution of structural disorder and environmental interactions has a remarkable effect on the spectroscopy of self-assembled aggregates in solution and cannot be neglected to get an accurate description of the vibronic line shape of the absorption spectra.^35,47,48,51,52^ Clearly, the spectral line shapes hold a treasure trove of information regarding the nature of the fundamental electronic excitations and valuable insights into the way in which the chromophores pack together.

Although, as reported earlier, a qualitative understanding of the factors ruling the change of the absorption spectrum of PDI upon aggregation has been reached, viable computational approaches to describe the interplay among all these factors in a coherent and nonphenomenological way have been lacking until very recently.^35,52^ The scope of the present work is to introduce a novel mixed quantum-classical (MQC) computational protocol, hereafter named adiabatic molecular dynamics with generalized linear vibronic coupling (Ad-MD|gLVC), aimed at simulating the spectroscopy of aggregates in solution, while taking into account thermal fluctuations of solute–solute and solute–solvent interactions, vibronic effects, and interstate coupling for excitonic aggregates. This approach is employed to perform a complete study of the dynamics and spectroscopy of the *N*,*N*-bis(2-(trimethylammonium)ethylene) perylene-3,4,9,10-tetracarboxylic acid diimide (PDI) dimer, in both acetonitrile (ACN) and aqueous solution. Ad-MD|gLVC remarkably extends the capabilities of the Ad-MD|gVH method, recently proposed by some of us,^50^ introducing the possibility to treat systems with coupled electronic states. Because in these cases no analytical expression is available for the time-dependent correlation functions needed to compute the vibronic spectra, Ad-MD|gLVC implements a direct numerical propagation of the vibronic wave packet on coupled potential energy surfaces (PESs). To take into account all of the effects discussed earlier, Ad-MD|gLVC combines extensive classical molecular dynamics (MD) simulations with quantum dynamics (QD) wave-packet propagations. For the former, we use a molecule-specific quantum mechanically derived force field (QMD-FF), built according to the JOYCE protocol,^53−55^ that was recently benchmarked for the PDI monomer.^49^ For the latter, we use the multiconfiguration time-dependent Hartree (MCTDH) QD wave-packet propagations,^56,57^ and its multilayer (ML) generalization,^58^ in combination with a linear vibronic coupling (LVC) Hamiltonian^59^ parameterized with a fragment-diabatization approach recently developed by some of the authors.^60^ The latter technique allows for defining the adiabatic electronic states of the PDI dimer as a combination of diabatic local excitations (LEs) of the PDI units and relative CT states. Similarly to the previous Ad-MD|gVH method, to account for the dynamics on the calculation of the spectra, solute plus solvent nuclear degrees of freedom are adiabatically separated into stiff and soft modes, including the fast stiff modes at the quantum mechanical (QM) level, while the slow soft modes are treated classically. The MD simulations sample a thermodynamically reliable set of large amplitude soft modes, and, at each snapshot extracted from the MD trajectory, the coupling between soft and stiff modes is introduced by parameterizing, in the subspace of the stiff coordinates, specific vibronic LVC Hamiltonians describing the coupled states. The final spectrum is eventually computed as a conformational average of the nonadiabatic spectra, obtained by Fourier transforming the QD correlation function of the stiff modes in each individual MD configuration.

We will show that the Ad-MD|gLVC approach permits both the reproduction of steady-state spectra and the investigation of the ultrafast nonadiabatic dynamics of the photoexcited aggregate involving local and CT excitations. While here Ad-MD|gLVC is applied to PDI dimers in both acetonitrile and water, the protocol is general and can be applied to a variety of molecular aggregates, even in more-complex environments than a homogeneous solvent. It may be worth anticipating that Ad-MD|gLVC has a significant, but still affordable, computational cost. Its strength, however, is that it is nonphenomenological so that each parameter is rigorously connected with a microscopic variable and can therefore be computed from first principles. Due to these characteristics, Ad-MD|gLVC can allow a detailed atomistic understanding of the factors determining the photophysics of the system, permitting one to examine, or reexamine, the robustness of phenomenological reduced-dimensionality models and to perform the calculations in complex systems when the assumptions on which such models are based may not hold. The paper is organized as follows: sections 2 and 3, respectively, introduce the methods and computational details, section 4 is devoted to the presentation of the results, and section 5 reports the discussion and some concluding remarks.

## 2 Ad-MD|gLVC Method

### 2.1 General Workflow

**Figure 1:**
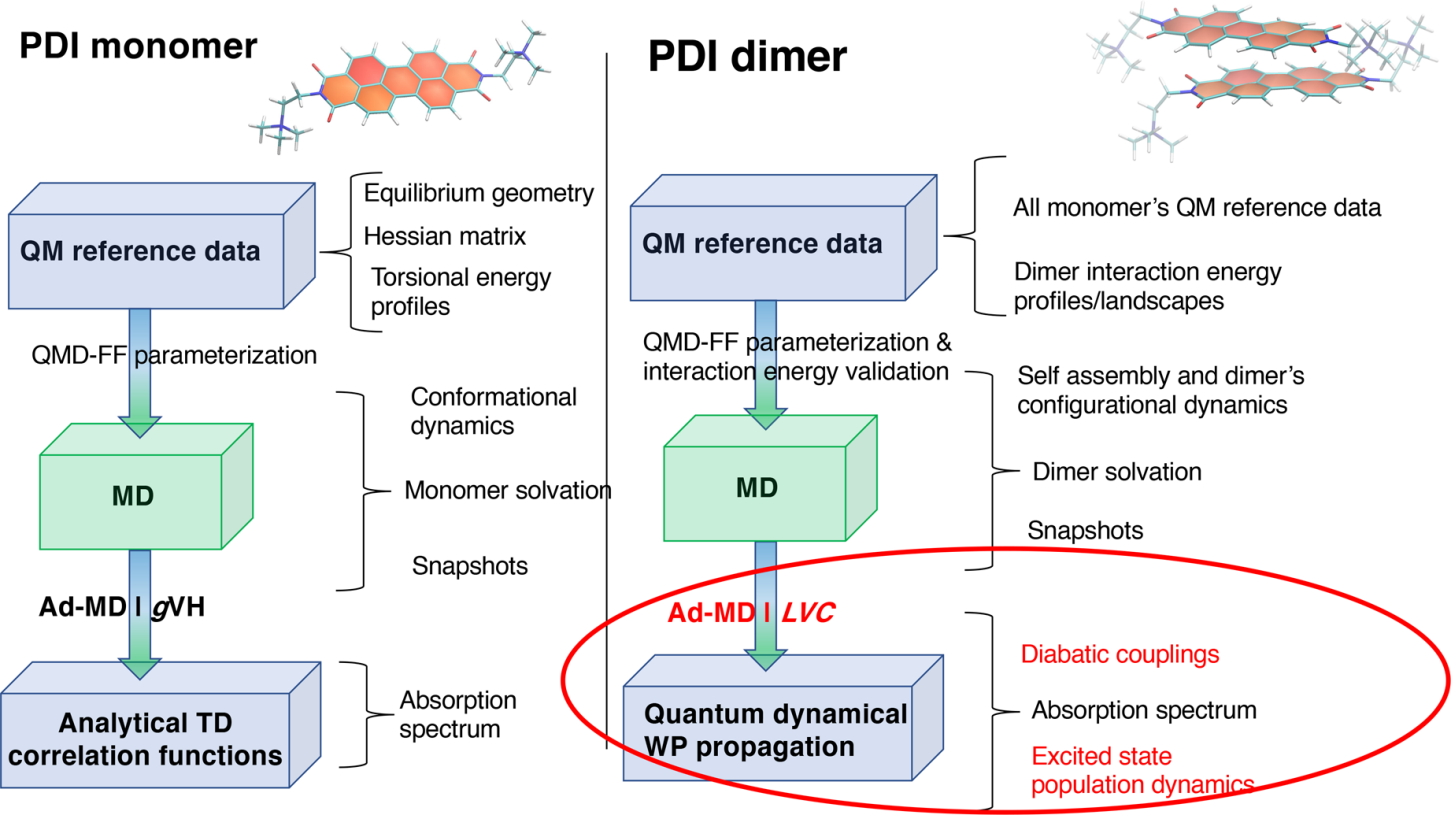
Comparison between the Ad-MD|gVH scheme (left)^50^ adopted in ref (49) to study the PDI monomer and the new MQC protocol (right) presented in this work for self-assembled dimers. In the boxes we highlight the main computational steps, while to the right of the brackets we indicate the main outputs. The key differences are highlighted with a red circle, whereas the QM and classical level of theory of the calculations are indicated in blue and green colors, respectively.

The computational scheme presented in this work is a generalization to nonadiabatic cases of the MQC protocol named Ad-MD|gVH, which is briefly sketched in the left side of Figure 1 and was developed by some of the authors^50^ and recently applied to compute the vibronic absorption spectrum of a solvated PDI monomer.^49^

As shown in the right panel of Figure 1, when dealing with more than one PDI unit, the computational protocol requires a number of generalizations, the most important of which is evidenced in red and concerns the calculation of the final absorption spectrum. In brief, we modify the vibronic calculation engine, substituting the vertical Hessian (VH) model,^61^ based on noninteracting harmonic PESs, with an LVC model of coupled harmonic PESs.^62^ As a consequence, we change the computational technique from the evaluation of analytical time correlation functions to numerical quantum dynamical propagations. In more detail, like the Ad-MD|gVH scheme,^50^ the new protocol is based on a partition of the nuclear degrees of freedom in stiff coordinates **r** (to be treated at the QM level) and much slower soft coordinates **R**, comprising the flexible coordinates of the solute and all of the solvent coordinates, to be treated at the classical level. The final absorption signal can then be retrieved from an average of quantum vibronic spectra computed in the stiff modes subspace, where the average is taken over the conformational space spanned by soft coordinates sampled by a classical MD run, carried out with purposely tailored QMD-FFs.^55,63^ Concretely, the MQC expression for the spectral line shape is^50^
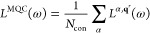
1where the sum is made on *N*_con_ representative snapshots α taken from the classical MD trajectory. Each spectrum *L*^α,**q**^*r*^^(ω) is computed in reduced-dimensionality normal-coordinates **q**^*r*^ obtained by projecting out all of the flexible coordinates **R**, but it is specific for the snapshot α because the PESs for **r** coordinates are recomputed at the specific value of **R** in that snapshot. In Ad-MD|gVH, the initial- and final-state PESs along **q**^*r*^ are assumed to be harmonic, and interstate electronic couplings are neglected. Each PES is constructed from energies, gradients, and Hessians at the **R** value, a procedure that we defined as the generalized vertical Hessian (gVH) model, as Hessians are computed out-of-equilibrium.^50^ In this framework, spectra can be computed straightforwardly because the necessary correlation functions are analytical.^65^ Such an approach is perfectly suited for the PDI chromophore, which possesses a single bright (HOMO → LUMO) transition in the low-energy range, well-separated from other electronic states. However, the assumptions behind Ad-MD|gVH do not hold when addressing the spectroscopic behavior of a PDI dimer. In fact, in this case, one needs to consider the couplings between pairs of four different diabatic states, as displayed in Figure S2: a local excitation on each monomer, |*L*_1_⟩ and |*L*_2_⟩, and two charge-transfer states, arising from transitions between HOMO and LUMO located on different monomers, i.e., |CT(1 → 2)⟩ and |CT(2 → 1)⟩. To this end, as evidenced in the bottom right part of Figure 1, in Ad-MD|gLVC the gVH model is replaced by a more-complex generalized LVC Hamiltonian, parameterized at each snapshot with a diabatization procedure. Because of interstate couplings, time correlation functions between different diabatic states cannot be computed analytically in the LVC model but must be obtained by numerical propagation of the vibronic wave packets on the coupled PESs according to quantum dynamics.^66^ Here we adopt the MCTDH approach,^56,57^ as detailed in the following section.

### 2.2 Computation of the Absorption Spectrum with the LVC Hamiltonian

We consider the following LVC Hamiltonian for a coupled set of diabatic electronic states |**d**⟩ = (|*d*_1_⟩, |*d*_2_⟩, ..., |*d*_*n*_⟩)
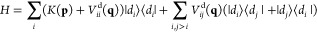
2

Here **q** are the dimensionless normal mode coordinates of the ground electronic state S_0_, **p** are their conjugate momenta, and *i* and *j* label the electronic states. The kinetic *K* and potential *V* terms of the Hamiltonian are defined as
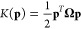
3
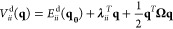
4
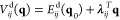
5where ℏ = 1, **Ω** is the diagonal matrix of normal-mode frequencies ω_*k*_ for mode *k*, *E*_*ii*_^d^(**q**_**0**_) are the diabatic energies, and *E*_*ij*_^d^(**q**_**0**_) are the electronic coupling constants between diabatic states at the reference geometry **q**_**0**_. Notice that these latter coupling constants do not appear in the standard LVC approach^59^ but are expected to exist and be important in the exciton/charge-transfer diabatic models. The vectors **λ**_*ii*_ and **λ**_*ij*_ (with *j* ≠ *i*) are the gradients of the diabatic PESs and the interstate couplings, respectively.

In a TD framework, the absorption spectrum ϵ(ω) at 0 K can be expressed as
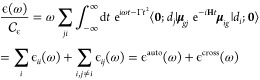
6where **μ**_*gj*_ = ⟨*g*|**μ**|*d*_*j*_⟩ are the matrix elements of the electric dipole moment, **0** is the ground-vibrational state of the ground electronic state *g*, whose energy is set to 0, and  contains all of the physical constants (its expression and its value to give molar absorptivity in units of *M*^–1^ cm^–1^ can be found in refs (67) and (68)). Moreover, a quadratic damping term ruled by a parameter Γ was introduced, corresponding to a Gaussian broadening in the frequency domain. By construction, diabatic states are built to be virtually independent of the nuclear coordinates. This implies that the elements **μ**_*gj*_ can be considered constant (Condon approximation) and the problem can be reduced to the computation of the time correlation functions
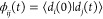
7

This step must be performed numerically, propagating the initial wave packets |*d*_*j*_(0)⟩ on the coupled PESs of the LVC Hamiltonian. The cross-correlation functions ϕ_*ij*_(*t*) (*j* ≠ *i*) are required to obtain the terms ϵ_*ij*_(ω), which modulate the spectral shape, although they do not contribute to the total intensity. Once the different ϕ_*ij*_(*t*) are computed, the total correlation function, ϕ_tot_(*t*), is obtained by a weighted sum where the weight on each term is the scalar product of the electric transition dipole moments of the corresponding states:
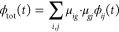
8

The final absorption spectrum is eventually obtained by Fourier transforming ϕ_tot_(*t*) (eq 6). The derivation of analytical expressions for the total intensity (*M*_0_) and the center of gravity (first moment, *M*_1_) of the absorption line shape predicted by our model is straightforward (see section S1.3 in the Supporting Information). For an ideal PDI dimer with two degenerate local states, |*L*_1_⟩ and |*L*_2_⟩, and two completely dark CT states, |CT_12_⟩ and |CT_21_⟩, *M*_1_ becomes
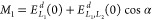
9where *E*_*L*_1__^*d*^(0) is the first moment of the monomer spectrum and α is the angle between the transition dipoles of the two states. In a stacked dimer fully eclipsed (α = 0), the so-called exciton electronic coupling *E*_*L*_1_,*L*_2__^*d*^(0) is expected to be positive, and therefore the spectrum of the dimer blue-shifts with respect to the monomer by the extent of *E*_*L*_1_,*L*_2__^*d*^(0).

### 2.3 Implementation

#### 2.3.1 Ad-MD**|**gLVC Full Route

As displayed in Figure 1, the new Ad-MD|gLVC scheme does not significantly differ from the previous Ad-MD|gVH procedure in the production of a set of reliable snapshots through the MD runs.

**Figure 2:**
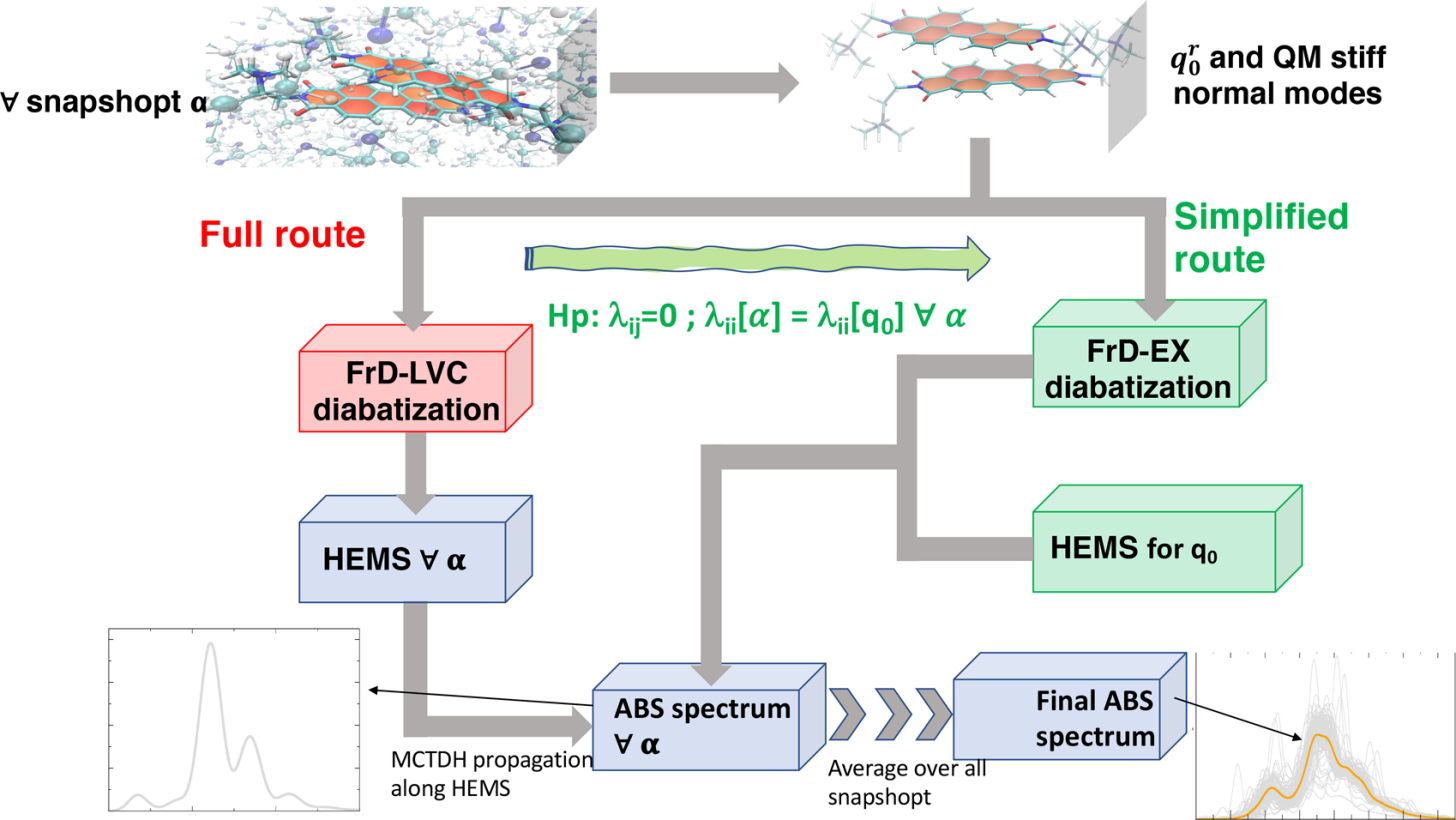
Ad-MD|gLVC workflow of both the full route (left side) and the simplified one (right side). The red boxes indicate the computationally more-demanding steps, whereas green boxes indicate the steps where the cost in terms of CPU time is significantly reduced. See the text for definitions of the employed acronyms and symbols.

As already mentioned, the main difference stands in the computational protocol applied to each sampled snapshot α. All the steps required by the full (i.e., without any further assumption) Ad-MDg|LVC procedure are sketched in the left side of Figure 2. It should be noticed that, while in Ad-MD|gVH we account for differences in the quadratic terms of the final and initial PESs so that they can have different normal modes and frequencies, in Ad-MD|gLVC we assume that all diabatic states have the same Hessian as the ground state (GS) (see eq 4). An analogous approximation transforms the VH model into the simpler vertical gradient (VG) one.^61^ To account for the effect of these quadratic terms, we would need to replace the LVC model with the so-called quadratic vibronic coupling (QVC) one,^62^ but this is computationally too expensive and LVC is enough to capture the main effects we want to study. Conversely, considering that the straightforward application of the full Ad-MD|gLVC protocol is also rather time-consuming, we have also designed a much faster simplified procedure, which is displayed in green in the right branch of the same figure. In practice, according to the full Ad-MD|gLVC scheme, for each snapshot α the following steps are necessary:

(1) *Reduced QM Hessian and ground-state equilibrium geometry***q**_**0**_^*r*^. The energy, gradient, and Hessian of the GS are computed at a proper QM level. The flexible coordinates are projected out and considered frozen, normal modes **q**^**r**^ describing only the stiff coordinates are computed, and a GS equilibrium geometry along these modes is extrapolated assuming harmonic approximation. Such a geometry **r**_eq_^α^, as well as the normal modes **q**^**r**,α^ and their frequencies, are specific for the snapshot α.

(2) *FrD-LVC diabatization*. The full LVC Hamiltonian is built up by displacing the **r**_eq_^α^ for positive and negative steps Δ_*k*_ along each mode *q*_*k*_^*r*,α^, performing a time-dependent density functional theory (TD-DFT) computation and a fragment-based diabatization (FrD) with a maximum-overlap criterion implemented in our in-house code Overdia. For this reason we indicate this LVC Hamiltonian with the label FrD-LVC. This step (shown in red in Figure 2) is the most time-consuming because it requires 2 × *N*_vib_^*r*^ + 1 TD-DFT computations for the dimer (where *N*_vib_^*r*^ is the number of stiff normal modes), plus the computation of the necessary overlaps between the transition densities necessary to apply the diabatization scheme.

(3) *Hierarchical effective mode selection (HEMS)*. The low- to medium-resolution spectrum can be computed in a very efficient way by adopting a hierarchical transformation of the LVC Hamiltonian in blocks of sequentially coupled effective modes.^69−71^ Such a hierarchy is obtained with a generalization of the Lanczos algorithm.^72^ Few blocks are sufficient to converge the spectrum.

(4) *MCTDH.* Nonadiabatic QD propagations of the vibronic wave packets are performed with the MCTDH method, and time-dependent correlation functions are computed.

(5) *Absorption spectrum*. Absorption spectra for each snapshot are obtained by Fourier transform of the total correlation function, and the final average spectrum is obtained according to eq 1.

#### 2.3.2 Ad-MD|gLVC Simplified Route

As commented in the previous list, the generation of the LVC Hamiltonian is the slow part of the protocol, and the repetition for a representative number of snapshots (∼100) demands a significant computational effort. Yet, two observations, shown in green in Figure 2, allow for a remarkable increase in speed. In the following we will prove that, for the PDI dimer, it is possible to neglect the linear terms in the interstate couplings in eq 5 (i.e., **λ**_*ij*_^α^ = **0**) and to assume that the diagonal gradients **λ**_*ii*_^α^ are independent of the specific snapshot α. The first approximation implies that we assume that the interstate couplings depend on the snapshot α because they depend on the fluctuations of the slow coordinates but are independent of the small oscillations of the fast coordinates **q**^*r*^. As far as the second approximation is concerned, different strategies can be conceived to estimate the snapshot-independent gradients **λ**_*ii*_. The protocol that we test and adopt in this work uses only computations for an isolated PDI monomer, optimized in its ground state in the gas phase. More specifically we assume that the relevant normal modes of the PDI dimer can be represented by the sum of the sets of the normal modes of two isolated monomers in the gas phase {**q**^*M*_1_^, **q**^*M*_2_^}, hence computing the gradient of the local excitation of the monomer (**g**^*L*^) and of the CT cation (**g**^*C*^) and anion (**g**^*A*^). In this framework, the gradients of the two local excitations |*L*_1_⟩ and |*L*_2_⟩ of the dimer and the ones of the two CT states, CT(1 → 2) and CT(2 → 1), can be eventually approximated by the vectors {**g**^*L*^, **0**} and {**0**, **g**^*L*^} and the vectors {**g**^*C*^, **g**^*A*^} and {**g**^*A*^, **g**^*C*^}, respectively. It is noteworthy that these gradients can be computed analytically and, therefore, much more rapidly than what is required for the parameterization of the LVC Hamiltonian according to the full route (see section 3.4). In practice, in this approximation only the diabatic vertical energies *E*_*ii*_(**q**_0_^*r*,α^) and interstate couplings *E*_*ij*_(**q**_0_^*r*,α^) (*j* ≠ *i*) need to be recomputed at each snapshot α. This operation is much faster than the generation of a full LVC Hamiltonian as it requires a single TD-DFT computation for the dimer and one for each of the two monomers, resulting in a huge speedup with respect to the full protocol. We note that such a single-point diabatization was first presented by some of the authors with the name “FrD-EX”,^60^ and it was used to parameterize a purely electronic excitonic model with only the terms *E*_*ii*_ and *E*_*ij*_. Therefore, in this new scheme in Figure 2, step 2 is replaced by the FrD-EX diabatization. Moreover, the generation of the HEMS is identical for all snapshots and can be performed just once, hence replacing step 1, which was carried out for each snapshot α. More technical details on the different steps of the computations are given in the following section.

## 3 Experimental and Computational Details

### 3.1 Experimental Section

UV–visible spectra were acquired in transmission mode with an Agilent-Cary 300 UV–visible Spectrophotometer in the 800–200 nm interval at a scan rate of 600 nm/min, with a spectral bandwidth of 2 nm and employing a quartz cell with an optical path of 2 mm. (PF_6_)_2_-PDI salts are not soluble in water, whereas they are soluble in acetonitrile (ACN) where, however, aggregation cannot be observed even for saturated solutions. Therefore, to observe PDI aggregation, 20 μL aliquots of 5 mM PDI in ACN (PDI@ACN) were progressively added to 2 mL of water up to a total 100 μL of ACN in water. After this point a further final addition of 50 μL of 5 mM PDI@ACN was accomplished. Alternatively, difference spectra reflecting the spectral changes due to PDI aggregation can be obtained by starting from a mother solution of PDI in ACN and adding increasing amounts of water. All difference spectra, along with the related experimental details, can be found in section S2.1.

### 3.2 Molecular Dynamics

The absorption spectrum of a Cl_2_-PDI salt solvated in ACN, which can be recorded experimentally,^10^ was previously simulated by us by means of the Ad-MD|gVH method, taking into account a single PDI monomer.^49^ Here we instead considered two Cl_2_-PDI units and computed Ad-MD|gLVC spectra in both ACN and water. Despite the computational burden, this choice was made, on the one hand, for an internal coherence when comparing the vibronic progressions of dimer and monomer (ACN) and, on the other hand, in view of a more realistic comparison with the experimental spectrum of the aggregates obtained in water. It is worthwhile to stress that Cl_2_-PDI solvation and PDI self-aggregation are observed with MD in both environments because of the resulting high concentration, notwithstanding the significant number (1–3 × 10^3^) of solvent molecules considered. The fact that the counterion dissociated in our simulations made immaterial the use of Cl^–^ or PF_6_; thus, we used chlorine for internal coherence with the MD simulations previously performed for the monomer.^49^ As discussed in detail in the following, negligible differences arose in the final computed spectra between the two solvents; hence, for the sake of brevity, most of the results in water will be reported in the Supporting Information, whereas the discussion in the main text, unless otherwise stated, refers to simulations in ACN.

An accurate QMD-FF for a PDI monomer was previously parameterized by some of the authors,^49^ as briefly summarized in the following. All intramolecular PDI parameters were obtained with the JOYCE code.^49,53,55^ As far as PDI’s intermolecular terms are concerned, the point charges were obtained through the RESP protocol by using the Antechamber suite, while the Lennard-Jones (LJ) parameters were transferred from the OPLS libraries.^49,73,74^ The accuracy of the description of the intermolecular interactions within the dimer, which was not considered in our previous work on the PDI monomer,^49^ is here carefully validated through the analysis of the MM interaction potential energy surface (IPES) along selected dimer arrangements. For solvents and counterions, the TIP3P model was employed for water, whereas the parameters concerning ACN and Cl^–^ were taken from the OPLS FF.^73,74^ Further details on PDI’s QMD-FF can be found in the original article.^49^

As far as MD simulations are concerned, two systems, PDI_2_@ACN and PDI_2_@H_2_O, were prepared by solvating into a cubic box two PDI units with ∼1000 ACN or ∼3000 H_2_O molecules, respectively. In both systems, four Cl^–^ anions also have been included to ensure the electroneutrality of the cell. The two PDIs were placed randomly in the simulation boxes at a distance between the centers of mass of ∼16 Å. Moreover, two sets of MD runs were carried out for PDI_2_@ACN, separately for the syn and anti conformers, with the aim to rationalize the role of the lateral pendants on self-aggregation. All MD simulations (in periodic boundary conditions) were computed by making use of the GROMACS 5.1 Engine. The PDI_2_@ACN and PDI_2_@H_2_O systems were initially minimized to avoid bad contacts and, subsequently, thermally equilibrated for ∼2 ns at 300 K in the NVT ensemble. Very long production runs were carried out in the NPT ensemble for 1 μs at 1 atm and 300 K and considering a time step of 1 fs using the LINCS algorithm^75^ to fix the bond distances involving hydrogen atoms. The temperature and the pressure coupling were described through Parrinello–Raman and the v-rescale schemes using coupling constants of 0.1 and 1 ps, respectively. Finally, for the short-range Coulombic and LJ terms, the cutoff radius was set to 11 Å, while the long-range electrostatics interactions were treated using the particle mesh Ewald (PME) procedure.

### 3.3 QM Calculations

**Figure 3:**
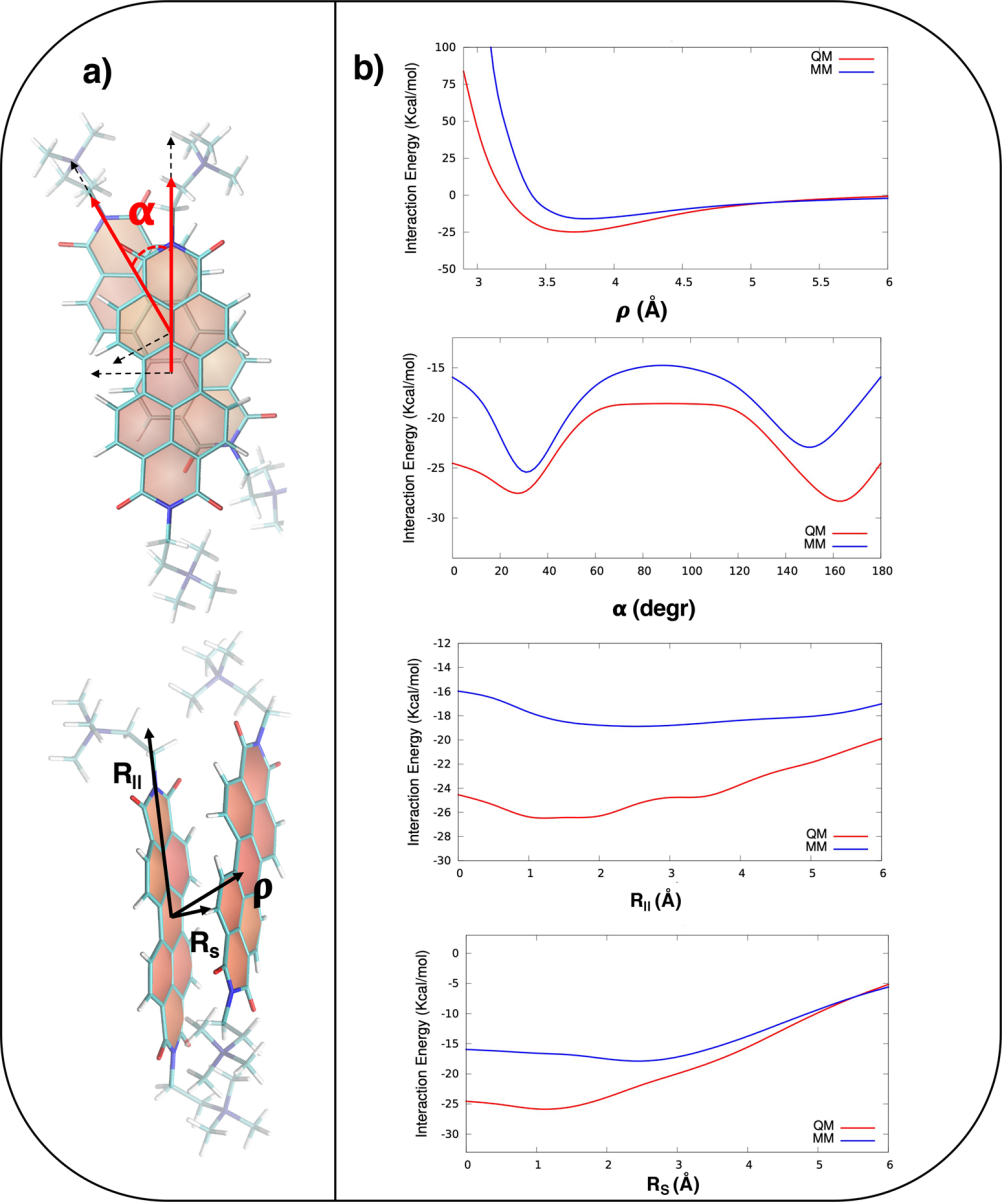
(a) Structure of the PDI dimer, where the arrows highlight the angles, distances, and displacements analyzed in this work. (b) Comparison between QM torsional relaxed energy scans (red lines) and relative MM relaxed profiles (blue lines) computed for the PDI dimer in ACN solution.

All QM calculations were carried out at the DFT and TD-DFT level, using the Gaussian 16 package^76^ with the CAM-B3LYP^77^ functional and the 6-31G(d) basis set and adopting Grimme’s D3 dispersion correction.^78,79^ The QM interaction energy between two monomers, which is necessary to benchmark the MM IPES, was computed along the coordinates displayed in Figure 3a, including the solvent effect at the C-PCM level to screen the electrostatic repulsion due to the +2 charge of the lateral chains. The same solvent description was applied in an unconstrained optimization of the ground-state PDI-dimer geometry, being employed as a reference to parameterize the full FrD-LVC Hamiltonian and performing TD-DFT calculations on molecular structures displaced along all of the reduced-dimensionality normal modes.

The QM calculations required for the MQC spectrum were carried out for each snapshot α extracted from the MD runs as described in the following. First, the reference geometry, **q**_o_^*r*,α^, optimized in the reduced space of the fast coordinates *r* was computed at the QM/MM level as described in section S1.4. Therein, we also showed for a few selected snapshots that alternative computational protocols to run the optimization led to only marginal differences in the determination of the **q**_o_^*r*,α^ structures and therefore of the diabatic energies *E*_*ii*_(0) and couplings *E*_*ij*_(0) for the PDI dimer. Next, to apply the FrD-EX diabatization scheme, single-point calculations were carried out at the TD-DFT level on each reoptimized geometry **q**_o_^*r*,α^ on both the monomers and the dimer. Calculations on the monomers were used to compute the reference states to define the diabatic states, whereas calculations on the dimer were performed to obtain the adiabatic states, which were then rotated to overlap with the reference states as much as possible. This operation defines the adiabatic-to-diabatic transformation, which was computed with a freely distributed code, Overdia, that was developed by some of the authors.^80^ Further details are given in the following section.

### 3.4 Fragment Diabatization

In all of the monomer and dimer calculations, a two-layer approach was adopted for taking the environment into account, again considering the electrostatic embedding (EE) of all solvent molecules and counterions within a radius of 20 Å, which were included as QMD-FF point charges (pc). Furthermore, in the monomer calculations, the electrostatic effect of the presence of the second monomer on the reference states was introduced by including it in the EEpc shell. We considered four diabatic states for the PDI dimer: two local excitations of the individual monomers and their two associated CT states. Reference states for the local excitations were obtained by reading the corresponding TD-DFT response vectors in the monomer calculations. As reference for the CT states, we defined states made up by an orbital transition from the highest occupied molecular orbital (HOMO) of the first monomer to the lowest unoccupied molecular orbital (LUMO) of the second one, and vice versa. Afterward, using a maximum-overlap-based fragment diabatization, we determined the combinations of the adiabatic excited states of the dimer that resemble the reference states as much as possible (see the Supporting Information for a sketch). This procedure^60^ defined an adiabatic-to-diabatic transformation matrix (**D**). Application to the diagonal matrix of the adiabatic (TD-DFT) energies of the dimer allowed for computing the diabatic vertical energies *E*_*ii*_(**q**_0_^*r*,α^) and couplings *E*_*ij*_(**q**_0_^*r*,α^). For the simplified route in Figure 2 (right), this operation needed to be done just once for each snapshot α. In fact, the interstate couplings were assumed to be independent of the oscillations of the fast coordinates **q**^*r*^, and the gradients of the diabatic diagonal potentials along **q**^*r*^ were taken to be independent of the snapshot α. These gradients were computed analytically with DFT for the cation and anion of the monomer for the CT states and with TD-DFT for the monomer local excitation. On the contrary, for application of the full protocol in Figure 2 (left), the gradients of the diagonal and off-diagonal LVC diabatic potentials (**λ**_*ii*_^α^ and **λ**_*ij*_^α^, respectively) were obtained by numerical differentiation.^81^ To do that, for each snapshot we performed 2 × *N*_vib_^*r*^ additional calculations, each at a structure displaced with respect to the equilibrium position **q**_0_^*r*,α^ by a small quantity Δ_*k*_ = ±0.02 along a single normal coordinate *q*_*k*_^α^, and then applied the transformation (**D**^α^) to the elements of the matrix of the adiabatic potential energies *V*^ad,α^(Δ_*k*_):
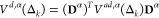
10
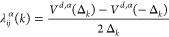
11

### 3.5 Quantum Dynamics of the Nuclear Wave Packets

Nuclear wave-packet propagations were performed using the MCTDH method^56,57^ as implemented in the Quantics code, with the specific settings shown in the Supporting Information.^82,83^ There are 224 fast coordinates of the PDI dimer, making a straightforward application of MCTDH very challenging. In these cases, fully converged low-resolution spectra, which account for the effect of all nuclear coordinates, can be obtained by exploiting a hierarchical representation of the Hamiltonian in terms of effective collective coordinates. They are divided in blocks, each comprising a number of coordinates, defined in such a way that the short-time dynamics (the only one relevant for the low-resolution spectrum) is dominated by a few blocks.^69−72,84^ In Figure S5 we show that 3 blocks (12 coordinates) already provide converged spectra for a Gaussian broadening with a half width at half-maximum (HWHM) of 0.03 eV and that including a fourth block results in very minor changes. Therefore, in the following, 3 blocks were adopted to compute the spectra of all snapshots. The number of coordinates necessary to accurately describe the dynamics increased with the time range monitored after the photoexcitation. Hence, to ensure fast and fully converged results, we used the Multi-Layer (ML) extension of MCTDH,^58^ ML-MCTDH. Indeed, this extension allowed us to include even the effect of all of the normal coordinates (44) with sizable couplings. QD simulations in 44 dimensions were actually run to compute the time evolution of the electronic populations up to *t* = 100 fs, as discussed in section 4.5.

## 4 Results

### 4.1 Validation of the Ad-MD|gLVC Simplified Route

As discussed in section 2, the significant cost of the full Ad-MD|gLVC protocol prompted us to devise the more computationally effective route displayed in Figure 2. For the specific case of the PDI dimer, the approximations over which such a simplified procedure is rooted were tested with care before systematically applying them along the MD trajectories. To this end, (i) we considered a single-dimer geometry in the anti conformation, namely, the optimized structure in ACN; (ii) we applied the full route by projecting out^49,50^ the coordinates of the whole flexible lateral chains (all bonds, angles, and dihedrals) as well as the intermonomer coordinates;^85^ and (iii) on this reduced-dimensionality model (from 450 to 224 normal modes), we parameterized a full LVC Hamiltonian and computed the static spectrum (see section S2.2 for further details). The absorption spectrum was thereafter also computed with the simplified route described in section 2.3 and obtained by setting **λ**_*ij*_ = **0** and . While further details are available in section S2.3, the results displayed in Figure S10 show that imposing **λ**_*ij*_ = **0** had almost no effect on the spectrum, whereas the second approximation only introduced modest changes. All dimer spectra presented in the following sections were therefore computed by adopting both approximations of the simplified route.

### 4.2 PDI Dynamics in Solution: Structural Properties

After validating the reliability of the LVC simplified route, we went beyond the static approximation, introducing the effects of the dynamics of both the PDI dimer and the solvent on the spectrum through MD simulations. PDI shows two distinct isomers (syn and anti) depending on the relative position of the flexible lateral pendants with respect to the plane defined by the π-core. As recently shown for the monomer case,^49^ although the two isomers had almost the same energy, the interconversion from one isomeric form to the other required overcoming a rather high energy barrier. For this reason, both the syn and anti isomers were separately considered. Despite the fact that the lateral pendants had a negligible effect on the monomer optical properties,^49^ in the case of aggregates, they were expected to play a significant role by influencing the stability and organization of the supramolecular π-stacked aggregate, thus indirectly affecting the final spectral shape.

Therefore, the first point to assess was the accuracy of the intermolecular FF in describing the aromatic π-stacking interactions, which significantly depended on the choice of the FF parameters.^86^ To this end, we compared the MM and IPESs computed at the QM level along the most relevant intermonomer coordinates, as displayed in Figure 3a. As shown in Figure S1, they were defined according to the molecular reference axes of each PDI aromatic core. Thereby, we defined the distance between the center of the two PDIs as ρ, which can be decomposed, as shown in Figure 3a, as the distance between the two PDI planes, R_π_, and the displacement along the short and long axes, R_*s*_ and R_∥_, respectively. Finally, we called α the angle between the two long axes and β (see Figure S1) the angle between the vectors normal to the PDI planes. It is worth highlighting here that, when both monomers bore the lateral pendants in the syn conformation, the aromatic cores were able to get closer and a larger portion of the IPES could be explored. Therefore, the FF IPES validation was carried out through the analysis of the earlier-mentioned geometrical descriptors and applied to the syn conformers. In Figure 3b, we compare IPES’s profiles along the selected geometrical descriptors computed at the QM and MM levels. As far as the distance between the centers of the aromatic planes is concerned, the top panel of Figure 3b shows that both approaches predicted the lowest interaction energy for ρ ≈ 3.6 Å, in agreement with the value expected for π-stacked systems.^32,87^ Hence, all of the other IPES scans were then obtained by keeping ρ fixed at 3.6 Å. In the second panel of Figure 3b, the QM intermolecular energy scan along the twist angle α shows two nearly degenerate minima, at ∼160° and ∼30°, whose positions are well-reproduced by the MM scan although with opposite relative stabilities. Also, the energy barrier separating the two minima was slightly overestimated at the MM level. Finally, for the IPES scans along the R_*s*_ and R_∥_ directions, displayed in the bottom panels of Figure 3b, we kept the two PDIs eclipsed; hence, α = β = 0° and ρ and R_π_ coincided (see section S1.1 for further details). DFT and MM scans both indicated that the attractive interactions along both axes were the greatest for displacements of ∼2 Å. These minima were slightly shallower at the MM level. Overall, the MM forces were expected to reliably reproduce the QM reference ones, confirming the accuracy of our FF in describing the interaction between monomers.

The validated FF was employed in NPT simulations of the solvated PDI dimer in ambient conditions to retrieve the statistical distribution of aggregate configurations required by the Ad-MD|gLVC method. To investigate the effect of side chains on the shape of the sampled geometrical arrangements, two separate starting configurations were considered, where the lateral pendants of both PDI monomers were placed in either anti or syn conformation. The time evolution of the geometrical descriptors defined in Figure 3a was monitored along both trajectories with reference to data in ACN, as this was the only solvent for which we ran MD simulations considering two different starting configurations. It is important to mention that, during the long μs dynamics starting with the separated monomers both bearing pendants in the anti conformation (anti–anti dimer), one monomer underwent an anti-to-syn transition, thus settling in an anti–syn arrangement. In Figure S11, we show that only one transition was observed in ACN during the μs dynamics, and it took place between 700 and 800 ns. Therefore, as evident by the chain conformational distribution shown in the same figure, when the monomers both started in the anti conformation, roughly 80% of the dimer configurations were found in anti–anti, while the rest were in an anti–syn arrangement. Nonetheless, because in the other MD run starting with a syn–syn configuration the opposite syn-to-anti transition was never observed, for the sake of simplicity the two MD runs were still labeled as anti or syn trajectories. Most importantly, it should be stressed that the anti → syn transition took place well after the PDI aggregate formation, which took place much more rapidly. In fact, in agreement with the conclusions drawn from the IPES analysis, Figure S14 shows the time evolution of ρ, which clearly indicated that, independently from the starting configuration, the two monomers formed a stable aggregate in <1 ns.

**Figure 4:**
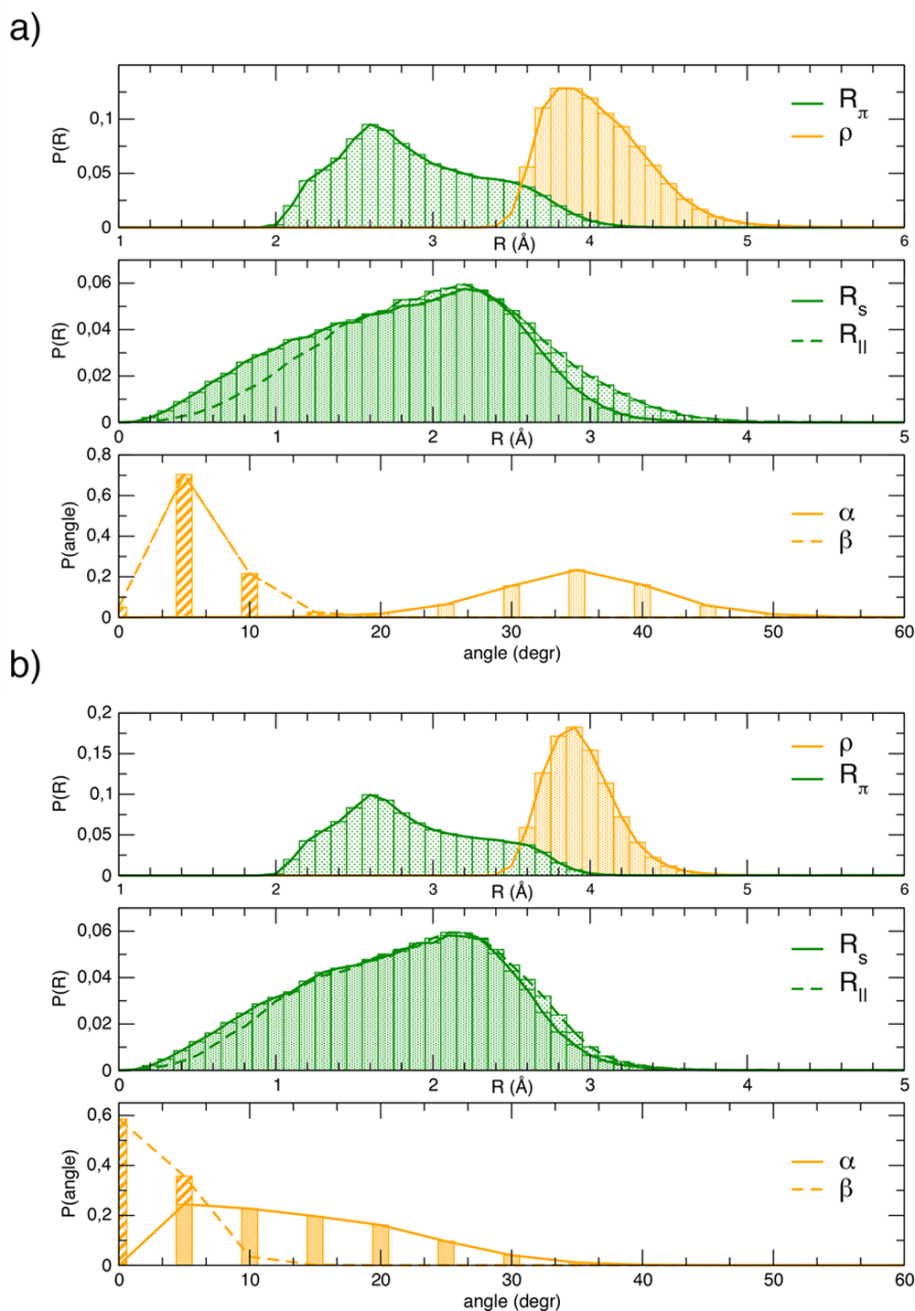
Distributions of intermolecular (PDI–PDI) angles, distances, and displacements as defined in Figure 3 computed along the MD trajectories of the PDI_2_@ACN starting with the (a) anti and (b) syn isomers.

The top panel in Figure 4a shows the distribution along the anti MD trajectory of the distances between the π planes (R_π_) and between the center of the PDI cores (ρ). The former shows a maximum ∼2.6 Å and a long tail at larger distances, whereas the latter shows a more narrowed distribution with a maximum ∼3.9 Å. The difference between these two distributions suggests that PDIs were almost never found perfectly superimposed. This was confirmed by the distributions of the displacements along the short (R_*s*_) and long (R_∥_) axes (the middle panel of Figure 4a), which showed a maximum ∼2.2 Å, and the ones of the α and β angles (bottom panel in Figure 4a), which indicated that the two PDIs are always twisted (α ≈ 20°–50°) and slightly tilted (β ≈ 0°–15°). The correlation among the different geometrical descriptors is shown in Figures S15–S17, where 2D heat maps are displayed for the ρ distance with the considered angles and displacements, computed along the anti trajectory. Although the most probable ρ distance was ∼3.9 Å, it was evident that, by displacing, twisting, or tilting one monomer with respect to the other, the dimer was able to visit regions where the two planar cores were closer, and a non-negligible population also was registered in the 3.6–3.9 Å range. By comparing the distributions of the considered geometrical parameters obtained in the anti and syn MD runs, one can assess the role of the lateral pendants in modulating the aggregate’s structure. The distributions of ρ, R_π_, R_*s*_, and R_∥_, displayed in Figure 4b, were essentially unchanged due to the position of the lateral chains. R_π_ had a right skewed distribution with a maximum around 2.6 Å, while ρ had an almost symmetric distribution that was slightly narrower compared to the one of Figure 4a, while still having a maximum around 3.9 Å. Similarly, R_*s*_ and R_∥_ had very left skewed distributions and showed maxima around 2.2 Å. Again, as evidenced from the ρ–R_π_ correlation in Figure S18, the two PDI cores were rarely found superimposed. The main difference between the two isomers instead was evident from the distributions of both α and β (bottom panels of Figure 4b). At variance with the anti trajectory, the syn isomers formed aggregates that populated more eclipsed (α < 20°) dimer arrangements, as evidenced from the distribution in the bottom panel of Figure 4b. In turn, this again reflected the possibility for the two monomers to reach closer intercore distances, as shown by the cross-correlation displayed in Figures S15 and S18. Conversely, as far as the β angle was concerned, the two PDI planes were more likely to be parallel when considering the syn isomer, as demonstrated by the narrowed symmetric distribution of β centered around 0° in Figure 4b.

Finally, with respect to ACN, the results in water, displayed in Figures S13 and S21, showed rather similar distributions of the chain conformation and the intermolecular descriptors, respectively. The only noticeable difference was in the anti → syn transition of one of the two PDIs, which occurred more rapidly (<100 ns). Consequently, the two PDI planes were on average more parallel, as evidenced by the distribution of the β angle, and more likely closer and stable, as illustrated by the narrowed distribution of ρ. Considering the results achieved in both solvents, MD simulations indicated that the PDI dimer was able to visit a plethora of accessible configurations, whose shape and incidence were driven by the subtle interplay of the conformation of the lateral pendants, the interaction with the solvent, and thermal fluctuations.

### 4.3 Parametric Study of Stacked Dimers

The structural fluctuations of the dimer discussed earlier were expected to impact the absorption spectrum and, hence, the excited-state dynamics through the electronic coupling between the LE and CT states. While a detailed discussion of the effect of the supramolecular dynamics on the final spectroscopic properties will be given in sections 4.4 and 4.6, here we first report a simple parametric study, aimed at rationalizing the effect of each of the geometrical descriptors introduced in the previous section on diabatic energies (*E*_*ii*_) and couplings (*E*_*ij*_). Specifically, we will focus our attention on the dependence of the excitonic coupling, *E*_LE–LE_(0); the LE–CT coupling, *E*_LE–CT_(0); and the CT–LE diabatic energy gap, Δ*E*_CT–LE_(0), upon varying the stacking and sliding distances R_π_, R_∥_, R_*s*_, the coplanar angle α, and the tumbling angle β. Notice that β actually is a solid angle; therefore, for simplicity we only considered the tumbling angles along the sliding directions, defined here as β(R_∥_) and β(R_*s*_).

**Figure 5:**
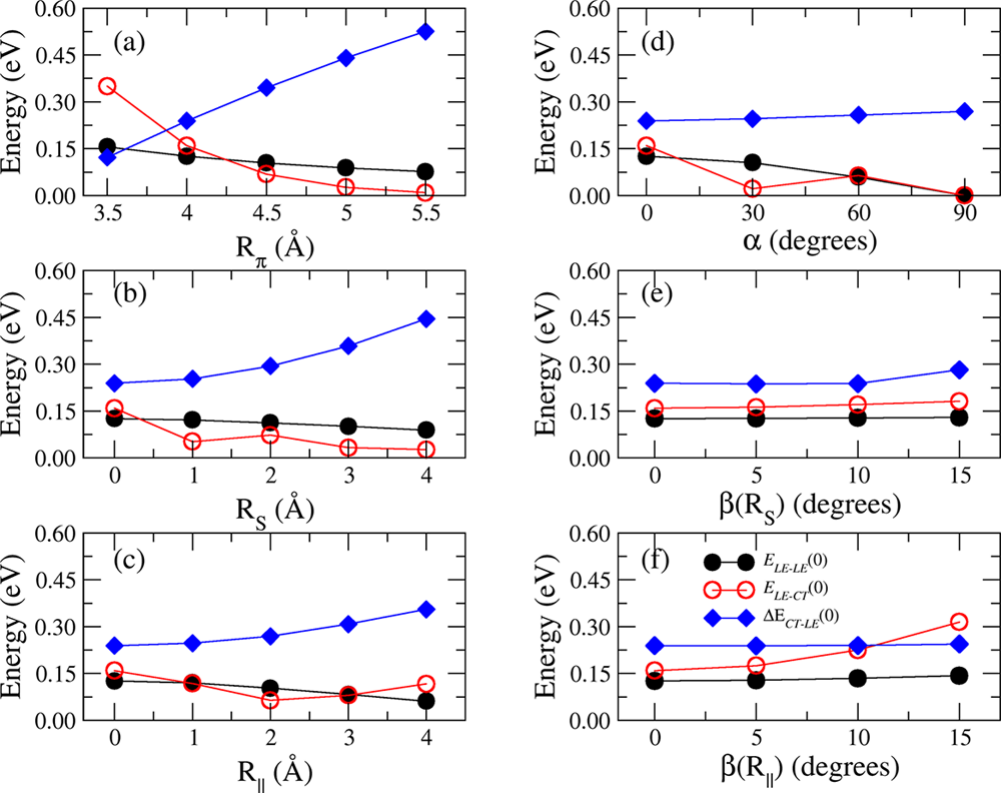
Dependence of the constant interstate coupling E_*ij*_(0) (for *i* ≠ *j*) and the LE–CT energy gap (Δ*E*_CT–LE_(0)) with different parameters of the PDI dimer: (a) R_π_, (b) R_*s*_, (c) R_∥_, (d) α, (e) β (component along R_*s*_), and (f) β (component along R_∥_). The scans were performed on the structure with R_π_ = 4.0 Å, changing one parameter at time and setting all others to zero. Because several choices of *E*_LE–CT_(0) are possible, we specify that only the *E*_LE–CT_ coupling between the states |*L*_1_⟩ and |CT(1 → 2⟩ is reported because it is larger in magnitude. By symmetry, the |*L*_2_⟩ and |CT(2 → 1⟩ coupling is the same.

In the qualitative spirit of this extended analysis, the dimers in this section were built with two ideal PDI monomers where lateral chains were substituted by hydrogens. Each monomer was in its equilibrium geometry (*D*_2*h*_ symmetry), and the starting reference consisted of two coplanar and perfectly superimposed monomers, from which each intermolecular displacement was separately applied. Figure 5 shows the dependence of the coupling terms on the geometrical descriptors, which were varied in the range suggested by the previous MD analysis. According to these results—and with the expected exception of the interchromophore distance *R*_π_—the dependence of the *E*_LE–LE_(0) coupling was in general small with the structural parameters and ranged between 0.08 and 0.12 eV. On the other hand, the *E*_LE–CT_(0) coupling was more sensitive to the structural changes of the dimer: it showed large fluctuations as the structure changed on R_*s*_ and β(R_∥_) and a very large decrease with increasing R_π_, but it was almost independent of β(R_*s*_). Interestingly, CT states were less stable than LE ones, but the energy gap decreased with the decrease of the three distances *R*_*s*_, R_∥_, and, especially, *R*_π_. For *R*_π_ < 3.9 Å, this gap was smaller than the coupling *E*_LE–CT_(0), causing a large mixing of LE and CT states in the adiabatic states of the dimer. The absorption spectra corresponding to the dimer structures considered in these scans are shown in Figure S23. The effects were complex because all of the couplings changed at the same time. On the one hand, it is evident that larger couplings resulted in a red-shift, a decrease of the intensity of the lowest energy band, and an increase and blue-shift of the most intense peak. On the other hand, when the coupling decreased, the spectra progressively became similar to that of the monomer, characterized by a maximum of the intensity for the lowest energy band.

### 4.4 Ad-MD|gLVC Absorption Spectrum of the Dimer in Solution

We now move to the discussion of the PDI dimer spectrum in ACN and in water, computed with the Ad-MD|gLVC method, which accounts for the slow dimer and solvent dynamics, by averaging over 100 configurations of the soft coordinates sampled with the MD runs carried out in each solvent.

**Figure 6:**
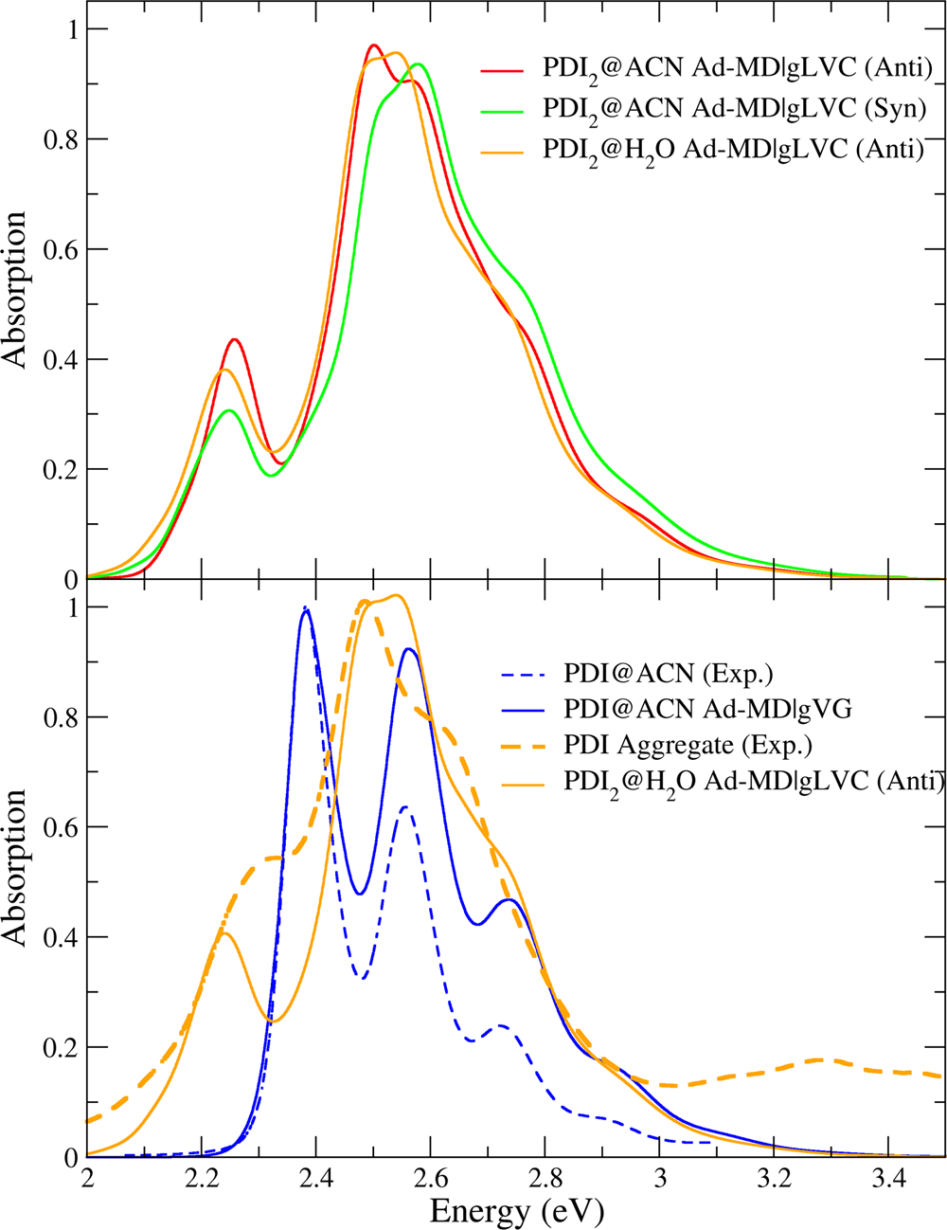
(Top) Ad-MD|gLVC for PDI dimer in ACN (from anti and syn MD runs) and water (anti MD trajectory). (Bottom) Comparison of Ad-MD|gVG (monomer) and Ad-MD|gLVC (dimer) spectra with the experimental spectra in ACN and water, respectively. The calculated spectra were red-shifted by 0.25/0.27 eV, and all stick transitions were convoluted with a Gaussian of HWHM = 0.005/0.03 eV for monomer/dimer, respectively. In the top panel, the spectra were scaled to have unit area and then rescaled by the same factor. For the bottom panel, the calculated spectra were scaled to match the experimental intensities.

Figure 6 shows the thermally averaged spectra of the PDI dimer computed along the anti and syn trajectories in ACN and compares them with the spectrum obtained for the monomer in the same solvent through the corresponding Ad-MD|gVG method (see section S2.8 for further details). As displayed in the top panel, the computed spectra with anti and syn trajectories are very similar, and the same holds for the anti spectra in ACN and water. In the bottom panel of Figure 6, our results are also compared with the experimental ones for the monomer in ACN^10^ and for the PDI aggregate obtained in this work by adding few drops of a PDI@ACN solution to water. Given the similarities, in this panel for the dimer we only report the computed spectrum of the anti trajectory in water. To ease comparison, computed spectra of the dimer have all been artificially shifted by 0.27 eV in order to match the maxima of the computed spectra of the dimer and the experimental spectrum of the aggregate (analogously the spectrum of the monomer was shifted by 0.25 eV). The Ad-MD|gLVC spectrum of the dimer reproduces very well the existence of two bands of the experimental lineshape of the aggregate, at 2.3 and 2.5 eV, and the shoulder at ∼2.7 eV. It is noteworthy that the blue-shift of the maximum of the experimental spectrum from the monomer to the aggregate was reproduced with a remarkable accuracy (with an error of 0.02 eV considering the different shifts applied for the monomer and dimer spectra). The fact that the lowest energy band in the dimer was red-shifted with respect to the monomer was also reproduced, although the extent of the shift was overestimated, indicating that the splitting of the two computed bands was too large. On the contrary, the relative intensity of the lowest energy peak with respect to the maximum peak (i.e., the ratio *R* discussed in the Introduction) was underestimated being slightly smaller for the syn configurations (see the top panel of Figure 6). It should be noticed, however, that the relative intensity of the first band was underestimated even for the monomer, and this inaccuracy was traced back^49^ to the use of CAM-B3LYP, by showing that a standard hybrid functional would produce the opposite effect. We remind the reader that the choice of a long-range corrected functional for the dimer was mandatory to get a proper description of the possible role of the intermonomer CT states. Nonetheless, a further analysis of the possible causes of such underestimation is reported for the dimer in section S2.9.

### 4.5 Analysis of the Factors Determining the Spectral Shape

**Figure 7:**
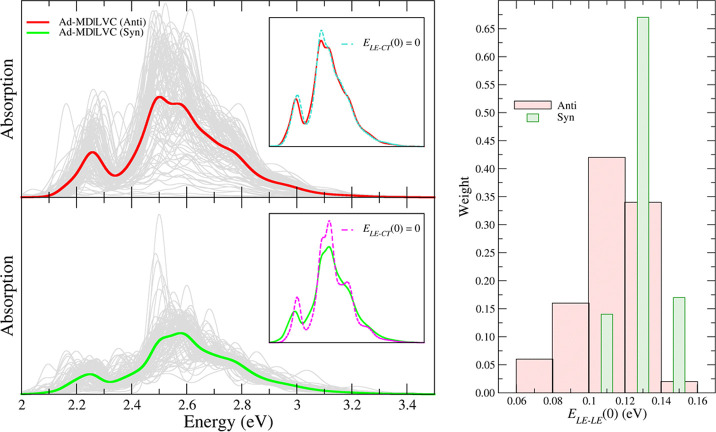
Left panels: Ad-MD|gLVC averaged (red and green lines) spectrum and individual (gray lines) spectra for the anti (top) and syn (bottom) MD trajectories of the PDI dimer. The insets show the averaged spectrum computed with or without accounting for the *E*_LE–CT_(0) coupling. All calculated spectra are red-shifted by 0.27 eV. Spectra are convoluted with a Gaussian of HWHM = 0.03 eV. Right panel: Distribution of the *E*_LE–LE_(0) coupling along the anti (red) and syn (green) MD trajectories. The sampling interval employed for *E*_LE–LE_(0) is the same for both sets and corresponds to the one shown by the red bins; syn data are reported with narrower bins only for clarity.

The separate vibronic spectra computed for each snapshot sampled along the anti and syn trajectories in ACN are shown as thin gray lines in the left panel of Figure 7, while the same spectra are reported for water in Figure S29.

**Figure 8:**
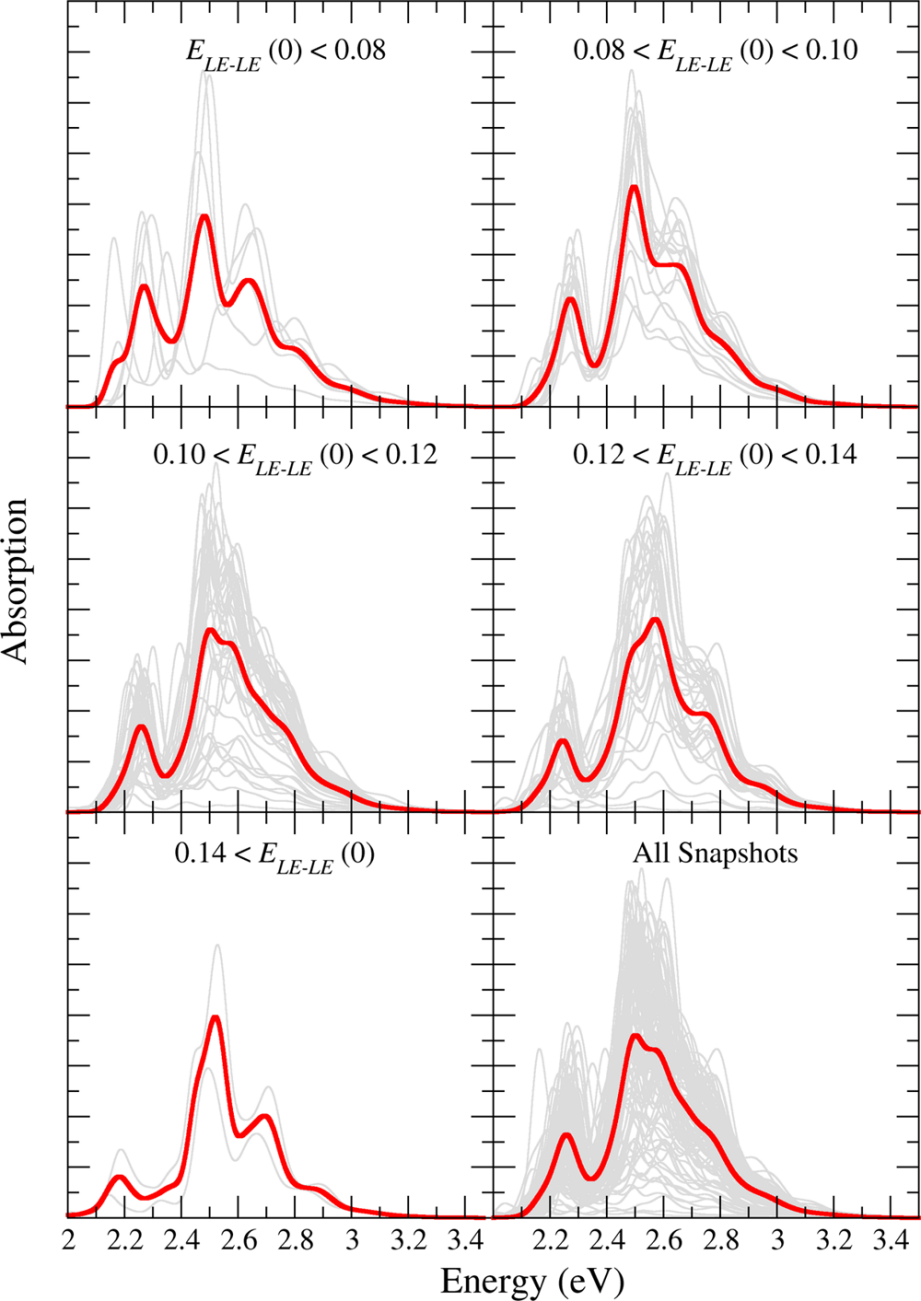
Calculated spectra of a set of snapshots with different values of the *E*_LE–CT_(0) coupling. The PDI dimer had lateral chains in the anti conformation. All of the calculated spectra were red-shifted by 0.27 eV, and all stick transitions were convoluted with a Gaussian of HWHM = 0.03 eV.

The total spectrum, displayed in Figure 7 and evidenced through thick lines, clearly resulted in a broadening and smoothing of the different peaks exhibited by the many individual signals, which, however, were not wiped out by averaging, in agreement with what was observed in the experiment. Actually, the computed spectra were even slightly too wide with respect to the experiment, and this may be attributed to the broadening Gaussian functions employed. In fact, the Ad-MD|gLVC method already includes all of the main possible sources of broadening, and so a phenomenological function would not be required. However, computational feasibility allowed us to use only a small number of effective coordinates, therefore a small broadening with an HWHM of 0.03 eV was applied to overcome this issue. To investigate the effect of the coupling between the bright LE and dark CT states, in the insets of the left panels of Figure 7 we compare the average spectra computed including or neglecting (*E*_LE–CT_(0) = 0) the LE–CT contribution. Notably, the differences were quite modest, indicating that the CT states had a marginal effect on the shape of the absorption spectra. This was not simply an averaging-out effect, as the spectra of the individual snapshots also showed only a moderate CT contribution. It is interesting that for this solvated dimer the CT states did not play a large role in the spectral shape, while for perylene aggregates they were shown to have significant effects.^13,14^ Of further interest is that this did not mean that the CT states were not populated after photoexcitation, as we will show in the following section. The exciton coupling *E*_LE–LE_, on the contrary, had a large impact on the spectral shape. The right panel of Figure 7 shows that it ranged from 0.06 to 0.16 eV for the different snapshots of the anti trajectory, although most of them (∼75%) exhibited a coupling between 0.10 and 0.14 eV. The effect of such a coupling on the spectral shape was further investigated for the anti trajectory by collecting, in the different panels of Figure 8, the individual and averaged spectra corresponding to exciton couplings falling into the same bins considered in Figure 7. It is apparent that, as the exciton coupling increased, the lowest-energy band became weaker with respect to the maximum band, in agreement with the results reported by Spano, showing that for H-aggregates the ratio *R* between the first two vibronic peaks decreased by increasing excitonic coupling.^12,18^ In addition, the splitting between the two bands also increased with the coupling: for snapshots with *E*_LE–LE_ ≈ 0.08 eV, the gap between both bands was 0.2 eV, while for *E*_LE–LE_ > 0.14 eV, it was 0.3 eV.

The different ratios *R* found in the simulated spectrum of the dimer with respect to the one experimentally obtained for a PDI aggregate could therefore be due, at least partially, to the value of the exciton coupling. On the one hand, as reported in Table C of the Supporting Information, the exciton coupling did not change significantly either upon variation of the functional or if it was computed from the Coulomb coupling of the transition densities^88,89^ rather than with the present diabatization scheme, thus confirming the robustness of our estimates. On the other hand, the present results strongly point to a connection between the extent of the exciton coupling and the spectral shape. For instance, according to Figure 7, the distribution of such couplings over the syn trajectory was narrower than that for the anti one, and its tallest bin was shifted at larger values, in agreement with the higher number of aligned dimer arrangements seen by the dynamics (see Figure 4). These findings explain why the ratio *R* was lower for syn (larger couplings) and why the fluctuation of the spectral shapes of the different snapshots was smaller (narrower distribution).

**Figure 9:**
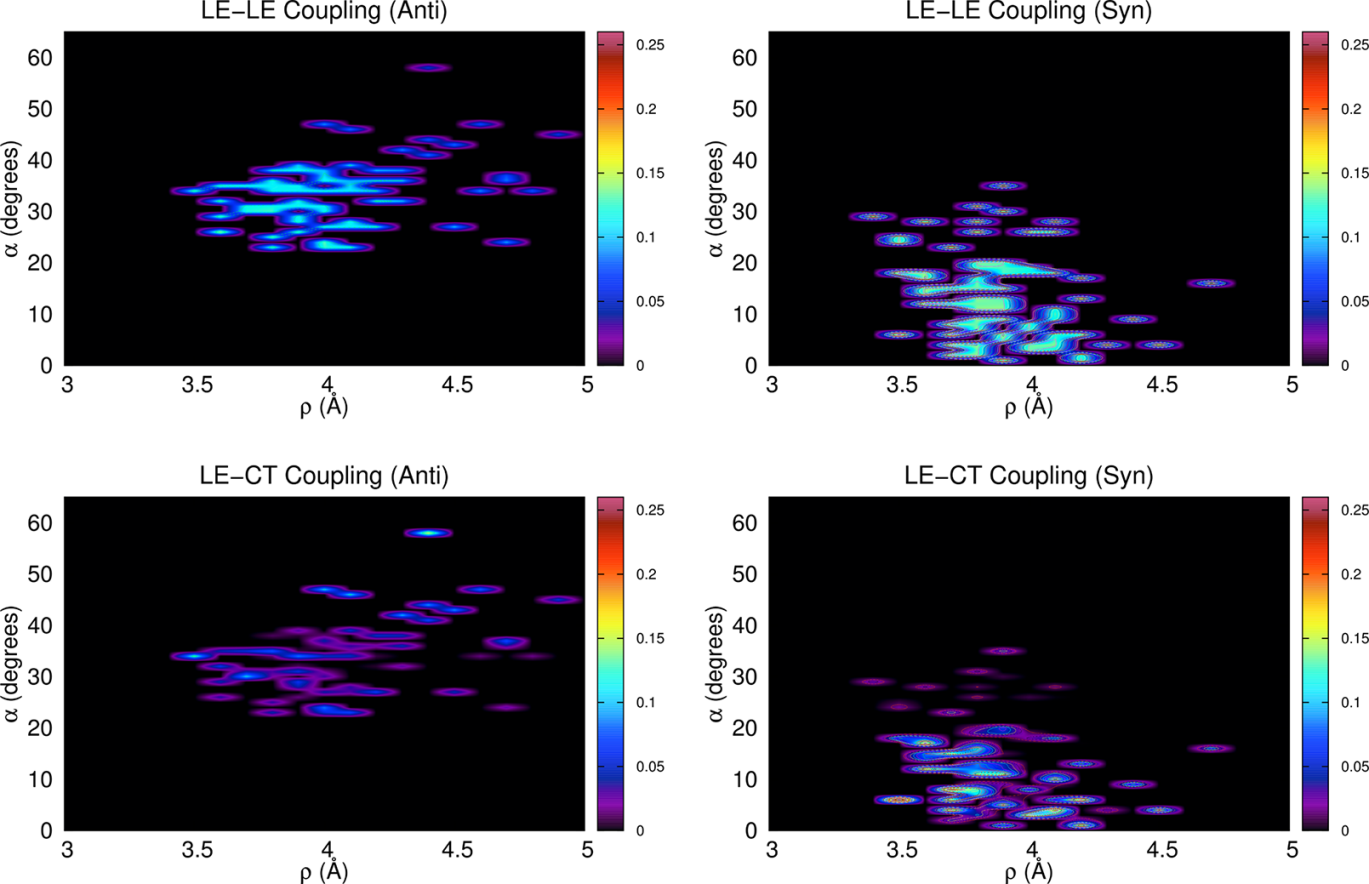
2D heat maps, correlating the intensity of the LE–LE coupling (top panels) and the LE–CT coupling (bottom panels) with the values of ρ (Å) and α (degrees) assumed from the dimer. For this plot we consider the snapshots extracted from the anti (left panels) and syn (right panels) MD trajectories. The color palette refers to the intensity of the interstate couplings (eV).

A more in-depth microscopic understanding can be achieved by correlating the magnitude of the *E*_*ij*_ terms (*i* ≠ *j*) with the variation, along the syn and anti MD trajectories, of the intermolecular descriptors defined in Figures 3a and S1. Here we focus on both the LE–LE and LE–CT couplings to separately analyze the different roles played on the spectral shape. Nonetheless, because LE–CT couplings were found to affect such shape only slightly, we display only the coupling between the |*L*_1_⟩ and |CT(1 → 2)⟩ states, which for the majority of the snapshots was the largest LE–CT coupling. As shown in Figures 9 and S30–S41, both *E*_LE–LE_ and *E*_LE–CT_ were larger for the syn aggregate, revealing that the diabatic couplings were generally larger for configurations showing the two PDIs in a more cofacial arrangement. This analysis also showed that the magnitude of the LE–CT couplings was more sensitive to the geometric variations of the aggregates observed along the MD trajectories compared to the LE–LE ones. This outcome agreed well with the considerations drawn for the parametric study reported in section 4.3 for the unsubstituted PDI dimer. In terms of the absolute value, the *E*_LE–CT_ terms may reach higher values (up to ∼0.26 eV for the syn aggregate) compared to the *E*_LE–LE_, although for most of the snapshots, and therefore for most stable PDI dimer configurations, these terms were significantly smaller.

Up to now we have discussed the existing correlation between the extent of the exciton coupling and the ratio *R*. However, Figure 6 shows the computed *R* was already too large in the monomer, and such a finding could only be due to an overestimation of the energy gradient of the LE state (i.e., the **λ**_LE–LE_ vector). Looking for a qualitative understanding of the interplay between **λ**_LE–LE_ and *E*_LE–LE_ in determining the spectral shape, in section S2.9 we examined the predictions of a minimal two-states, two-coordinates model that was able to capture the main features of the spectrum. It included the two local excitations, *L*_1_ and *L*_2_, and the two coordinates, *q*_1_ and *q*_2_ (i.e., the first members of the hierarchy), which described the displacement of the *L*_1_ and *L*_2_ equilibrium positions. In such a small model, **λ**_LE–LE_ became a scalar, λ. Figure S28 shows that *R* decreased with the increase of *E*_*L*_1_,*L*_2__. On the contrary, *R* increased with the increase of λ, as an indirect effect due to the transfer of intensity to the third band at 2.7 eV. The splitting of the two lowest bands increased with both *E*_*L*_1_,*L*_2__ and λ. While confirming that the slight disagreement on *R* with respect to the experiment was related to the values of the exciton coupling, this analysis also suggested that the excessive splitting of the two lowest-energy computed bands arose from the combination of the high values of the exciton coupling and the overestimation of the displacement of the equilibrium position of the LE state due to the employment of the CAM-B3LYP functional. Indeed, a similar overestimation of excited-state gradients/reorganization energies with the CAM-B3LYP functional was also noted for a PDI dimer in ref (52).

### 4.6 Time Evolution of the Diabatic Electronic Populations

**Figure 10:**
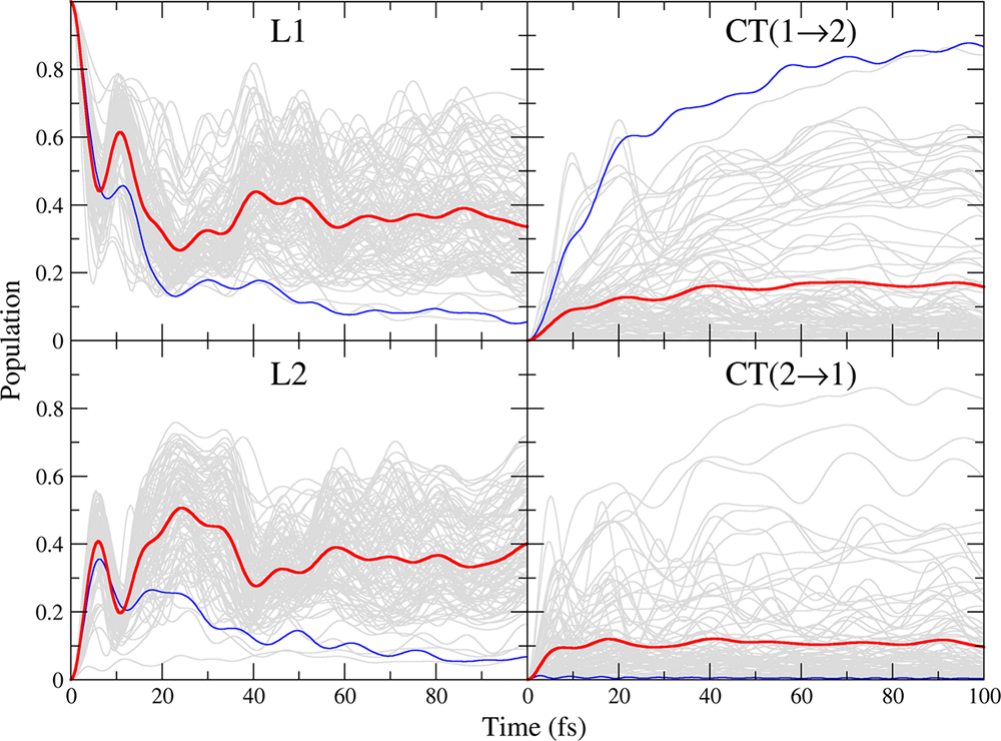
Population dynamics of the diabatic states |*L*_1_⟩ (top left), |*L*_2_⟩ (bottom left), |CT(1 → 2)⟩ (top right), and |CT(2 → 1)⟩ (bottom right) after the initial excitation on |*L*_1_⟩. The dynamics of individual snapshots are shown in gray, and the average is shown in red. The results for a specific case in which the populations of CT(1 → 2) and CT(2 → 1) are, respectively, large and vanishingly small are colored in blue. ML-MCTDH including 44 effective coordinates.

Although the main focus of the present article is on the absorption spectrum of a PDI dimer in solution, our approach also gave direct access to truly QD time-dependent observables, like the electronic populations of the different diabatic states. This is a noteworthy result because the QD simulation of nonadiabatic dynamics in the condensed phase is still an open challenge for current research.^90^ In Figure 10 we report the time evolution of the diabatic populations after an instantaneous excitation of the first PDI (state |*L*_1_⟩), for each of the snapshots extracted from the anti trajectory and their average. The latter, displayed in Figure 10 with thick red lines, represents the final QD prediction for the photoexcited PDI dimer in ACN solution in the limiting situation in which the environment was considered to be frozen during the excited-state dynamics. Such a limiting case, sometimes known as static disorder, has been considered by several authors in the recent past for different systems.^91−94^ Indeed, here we assume that the soft degrees of freedom—which comprise all solvent coordinates as well as those describing the PDI cores relative arrangement and the flexible dihedrals of the alkyl chains—are so slow with respect to the electronic dynamics that they can be considered as frozen in the configuration sampled by the MD run when the photoexcitation occurs. Then, as mentioned in the computational details, QD propagations were performed with the ML-MCTDH method including all of the relevant fast coordinates (44).

Figure 10 shows that immediately after photoexcitation of one monomer a fast population transfer occurred, and in a short time (∼60 fs) the averaged populations (red lines) reached limiting values. The final populations were 35–40% for both of the local excitations and 10–15% for the CT states, with a slightly higher population for CT(1 → 2), i.e., the CT state with a hole on the PDI monomer that was initially excited.

Interestingly, the population of the local excitations showed some quantum beats, which were more evident in the first 40 fs, due to the coupling with the vibrational motion: when the population of |*L*_1_⟩ was a maximum, the one of the |*L*_2_⟩ state was a minimum, and vice versa. On the contrary, the CT population underwent a nearly monotonic growth with some weak oscillations due to the vibrations. The differences in the dynamics observed in each snapshot, and individually displayed in Figure 10 with thin gray lines, highlight the sensitivity of the population exchange to the initial configuration of the slow degrees of freedom. In this respect, it is noteworthy that quantum beats survived even after the average was taken, indicating that the coupling of the electronic populations and the vibrational motion was quite strong. Additionally, despite the fact that the average population for CT states was ∼10%, for some specific snapshots much larger populations were observed, up to 60% in a few fs. Indeed, the CT state populations were much more sensitive to the specific snapshot than the LE populations. This finding can be rationalized by the fact that the CT diabatic energies and LE–CT couplings exhibited a larger distribution than the other energies and couplings (shown by the standard deviation of these values from the snapshots in Table D). Focusing on the results of a specific snapshot, highlighted in blue in Figure 10, it is interesting to notice that, whereas the |CT(1 → 2)⟩ state was steadily populated, |CT(2 → 1)⟩ was not. We verified that this behavior was systematic in other snapshots: when the |CT(1 → 2)⟩ was strongly favored, |CT(2 → 1)⟩ was not populated at all, and vice versa. This finding can be explained by the fact that, as shown in Table E in the Supporting Information, for the blue highlighted snapshot |CT(1 → 2)⟩ was the most stable state (0.35 eV below |*L*_1_⟩ and |*L*_2_⟩), whereas |CT(2 → 1)⟩ was remarkably higher in energy (+0.72 eV with respect to |*L*_1_⟩). Comparison to gas-phase results in the same table indicated that the stabilization/destabilization of the CT states was caused by the specific solvent configuration. This finding highlighted that the solvent electrostatic field could be strong enough to impart a preferential direction to the charge transfer, suggesting that the hole/electron separation could be very efficiently realized in PDI aggregates, in line with the experimental evidence reported at the interface between an n-type semiconductor and a water oxidation catalyst.^9^

## 5 Discussion and Conclusions

The general MQC computational method presented in this work aims to simulate the nonadiabatic vibronic spectra of molecular aggregates, coupling classical MD sampling, and QD wave-packet propagations. The protocol, named Ad-MD|gLVC, provides a robust framework to account for the effect of the fluctuations of both the aggregate and its embedding environment. The slow dynamics of the aggregate’s supramolecular structure, the flexibility of the alkyl chains, and the fluctuations of the explicit solvent molecules are accounted for with classical MD sampling, whereas the effect of the motion of the fast vibrations within PDI cores on the coupled surfaces of local excitations and charge-transfer states are included at the QD level (thus preserving the vibronic resolution). Ad-MD|gLVC largely extends the capabilities of Ad-MD|gVH, a MQC approach recently proposed by some of the authors for cases with negligible interstate couplings (see ref (50)) and applied to the monomer of PDI in ACN in ref (49).

Here we applied this method to the simulation of the absorption spectrum of a PDI dimer in ACN and in water. The results reproduced all of the trends observed experimentally when comparing the spectra of the PDI in monomeric and aggregate form. In particular, the blue-shift of the maximum and the inversion of the intensities of the first and second vibronic peaks were nicely reproduced by Ad-MD|gLVC, correctly predicting that the vibronic structure remains visible despite the fluctuations of the soft degrees of freedom and the environment.

The slight underestimation of the ratio *R* of the intensities of the first two vibronic peaks and the overestimation of their separation were traced back to the combination of two different factors: a too-large displacement after the local excitation (already observed in the case of the monomer and attributed to the use of the CAM-B3LYP functional^49^) and an overestimation of the exciton coupling. The latter suggests that, in the dimer arrangements sampled by MD, the two monomers were closer to each other with respect to the experimental aggregate. Considering the agreement found between the adopted intermolecular FF and the reference CAM-B3LYP/D3 interaction energy curves, such closer distances more likely can be attributed to the different number of PDI units in our model with respect to the experimental aggregate. It is likely, in fact, that interactions with neighbors on both sides of a PDI monomer will lead to greater intermonomer spacing within the aggregate than in the dimer. In summary, our approach allows one to accurately describe the difference of the spectral shape between monomers and dimers even if, in absolute terms, some inaccuracies in the relative intensities of the vibronic bands exist even for the monomer. DFT/TD-DFT usually offers a good compromise between accuracy and computational cost. However, to adopt our approach as a predictive tool, i.e., for species not yet synthesized, it might be beneficial to run a preliminary becnhmark analysis on similar systems to select the best DFT functional. Alternatively, it is in principle possible to couple our approach with different electronic-structure methods. This potentiality has already been shown for pyrene, adopting our code Overdia to parameterize an LVC Hamiltonian with multiconfiguration methods with perturbative corrections, like RASPT2.^95^ Extensions in combination with methods of the coupled cluster family, like the promising domain-based local pair natural orbital similarity transformed equation of motion–coupled cluster singles and doubles (DLPNO-STEOM-CCSD), are also possible.^96,97^

The Ad-MD|gLVC approach also allowed us to investigate the time evolution of the electronic populations after the photoexcitation. To this end, we assumed that the motion of the soft coordinates was much slower than the nonadiabatic dynamics of interest, a limit sometimes described as static disorder. Remarkably, although our results indicated that the existence of CT states altered only slightly the shape of the absorption spectrum of the dimer, they also pointed out that such states are partially populated in short time scales. More importantly, we showed that some specific arrangements of the adjacent PDIs, as well as of the solvent, can induce a marked directionality in the charge transfer, preferentially populating CT state 1 → 2 with respect to 2 → 1, or vice versa. This result is interesting for the investigation of the optoelectronic properties of PDI stacked aggregates, which are often adopted in photovoltaic devices, where charge separation and transport within the aggregates initiate the interfacial charge separation and collection.^9^ At the same time, the fast and effective population transfer between the two local excitations indicated that exciton migration/diffusion also might be very effective in PDI aggregates.

It should be mentioned that most of the calculations reported in this work have been performed with the simplified route of the Ad-MD|gLVC method described in section 2.3 because it allowed a remarkable reduction of the computational time. Its reliability was based on the assumption that the fluctuation of the dimer structure and solvent cavity mostly affected the vertical transition energies and the interstate couplings, whose dependence on the small oscillations of the fast coordinates could be neglected. Whereas for PDI_2_ these approximations were shown to be accurate, it will be interesting in future work to investigate if they can be challenged in different systems featuring, for instance, stronger intermonomeric interactions like hydrogen bonds.

Alternative routes to speed up the Ad-MD|gLVC calculations can also be envisaged. For instance, instead of running a different QD simulation for the LVC Hamiltonian parameterized at each snapshot α, it is possible to run a single QD simulation driven by a LVC Hamiltonian averaged over all of the snapshots. Of course, the two procedures are not expected to be equivalent, and the former approach that we adopted in this manuscript is in principle more correct. We compare results reported in this manuscript with those predicted by the averaged Hamiltonian for both the absorption spectrum and the population dynamics in the last section of the Supporting Information. Very interestingly, the spectrum compares nicely, even if not perfectly, although there is the need to adopt a larger phenomenological broadening to reintroduce the effect of the fluctuation of the LVC parameters that is lost in the averaging procedure. The comparison of the time evolution of the diabatic populations is also good, although, not surprisingly, those predicted by a single averaged Hamiltonian exhibit larger quantum beats, which are smoothed away when averaging the predictions of the snapshot-specific Hamiltonians. However, a significant difference that was noted is that the CT population predicted by the average Hamiltonian is remarkably smaller. This is a clear demonstration that, as already pointed out by some of the authors in ref (98), the outcome of nonadiabatic dynamics is not linear with the fluctuation of the parameters of the Hamiltonian. In a situation in which CT states are generally less stable than LE ones, their further destabilization in some snapshots leads to a minor effect on the CT populations. On the contrary, specific snapshots for which CT states are stabilized predict a very large population transfer toward these states, making the CT population averaged over the different dynamics larger than the one predicted by the average Hamiltonian.

In summary, combining classical MD sampling and QD simulations, our fully atomistic and nonphenomenological approach has allowed for an in-depth analysis of the correlation between the instantaneous structure of the dimer, the parameters of the diabatic Hamiltonian, and the predicted vibronic spectral shape and population dynamics, giving us the possibility to achieve a detailed microscopic understanding of the structural parameters determining the photophysics of the system. Among the other things, we found the following: (i) The exciton coupling is a key parameter for determining the spectral shape and has a coupled dependence on both the stacking distance and the rotation α, being larger for cofacial arrangements of the dimer. The orientation of flexible pendants plays a role in its modulation. On the contrary, the dependence of the exciton coupling on the twisting of the two monomeric planes is less relevant. (ii) Additionally, we discovered that the CT states are not important for the spectrum, although they gain non-negligible populations in the 100 fs time scale. Such a population would be underestimated without a careful description of the fluctuation of the dimer structure. It is worth highlighting that these aspects might have been missed by more reductionist approaches that compute the effect of one factor per time without considering their interplay. (iii) Finally, we determined that, although an effective mode for each monomer can qualitatively explain the shape of the spectra, a minimal, nonphenomenological model to accurately reproduce the vibronic spectral shape determined by all the fast coordinates is a chain of 6 sequentially coupled effective modes on each monomer.

The Ad-MD|gLVC method may be compared to similar recent coupled quantum-classical methodologies, which aim to reproduce absorption spectra and analyze photoexcited dynamics of perylenes in solution.^35,52^ First, the use of QMD-FFs in the MD simulations allows one to accurately consider the effect of the flexible side chains, a factor neglected in ref (52), where the authors assumed that the side groups have little influence on the relative geometries of the two perylene units. On the contrary, the present results indicate that some modulation of the final spectra can be connected with the conformational dynamics of the side chains, which does give rise to different dimer arrangements. This should be noted with the caveat that in ref (52) the perylenes were linked by a bridging phosphate group, thus limiting the conformational space sampled by the side chains with respect to two untethered PDI units. Next, our FrD methodology allows us to straightforwardly include both LE and CT states, while in ref (52) only LE were considered. However, both approaches can account for mutual polarization effects of closely spaced chromophores on the LE energies and couplings. Conversely, the effect of CT states was included in ref (35), but directly from the adiabatic TD-DFT states of the dimer rather than from a diabatization scheme as in the present work. Using rigorously defined LE and CT states from a diabatization scheme in principle can give a more straightforward interpretation of the photoexcited dynamics when adiabatic states have strongly mixed LE/CT character. Finally, both ourselves and the authors of ref (52) included nonadiabatic effects on the spectra, while in ref (35) the authors did not, as they calculated the spectra via single-point energy computations on MD snapshots with the nonadiabatic dynamics investigated separately using a surface-hopping approach. In ref (52) the nonadiabatic effects on the spectra are included semiclassically and with a density matrix formalism through the partially linearized density matrix (PLDM) method,^99^ whereas we utilize a quantum wave-packet treatment through the LVC model and MCTDH method. The PLDM method offers the advantage of more straightforward inclusion of temperature and dissipative effects, while the MCTDH method offers the advantage of numerical exactness, as well as wave-packet approaches typically being more numerically efficient than density matrix ones.^100^ However, recent ML-MCTDH calculations on PDI aggregates have included temperature effects through the thermofield dynamics approach,^101,102^ and this represents a promising future avenue for comparison of temperature effects on vibronic spectra via wave packet versus density matrix based methods.

In conclusion, in addition to the specific application presented here, we believe that the proposed Ad-MD|gLVC model will be a powerful tool in the close future to study the optoelectronic and photophysical properties of diverse molecular aggregates. In fact, although here we considered a dimer, application at longer oligomers is possible, thanks to the effectiveness of the hierarchical representation of the LVC Hamiltonians, and the simplifications of the full protocol introduced in the present article.

## Supporting Information Available

Theory and further computational details, additional results on model systems, experimental absorption and difference spectra, and supplementary 2D heat maps (PDF)
