# Supplementary material for: How the Interplay among Conformational Disorder, Solvation, Local, and Charge-Transfer Excitations Affects the Absorption Spectrum and Photoinduced Dynamics of Perylene Diimide Dimers: A Molecular Dynamics/Quantum Vibronic Approach
Source: J Chem Theory Comput. 2022 Apr 4;18(6):3718–36. doi: 10.1021/acs.jctc.2c00063 (PMC9202308; doi:10.1021/acs.jctc.2c00063)
Supplement: Supplementary file 1 — ct2c00063_si_001.pdf [file ct2c00063_si_001.pdf]

Supporting Information: How the Interplay among Conformational Disorder, Solvation, Local and Charge Transfer Excitations affects the Absorption Spectrum and Photoinduced Dynamics of Perylene-diimide Dimers: a Molecular Dynamics/Quantum Vibronic Approach

Alekos Segalina,[†,⊥] Daniel Aranda,[‡,⊥] James A. Green,[¶] Vito Cristino,[§] Stefano Caramori,[§] Giacomo Prampolini,[*,‖] Mariachiara Pastore,[*,†] and Fabrizio Santoro[*,‖]

†*Université de Lorraine & CNRS, LPCT, UMR 7019, F-54000 Nancy, France*

‡*Instituto de Ciencia Molecular (ICMol), Universidad de Valencia, Catedrático J. Beltrán 2, 46980, Paterna, Valencia, Spain*

¶*Consiglio Nazionale delle Ricerche, Istituto di Biostrutture e Bioimmagini (IBB-CNR), via Mezzocannone 16, I-80136 Napoli, Italy*

§*Dipartimento di Scienze Chimiche, Farmaceutiche ed Agrarie, Via Fossato di Mortara 17 44121 Ferrara, Italy*

‖*Istituto di Chimica dei Composti Organo Metallici, Consiglio Nazionale delle Ricerche, (ICCOM-CNR), SS di Pisa, Area della Ricerca, via G. Moruzzi 1, I-56124 Pisa, Italy*

⊥*Contributed equally to this work*

E-mail: giacomo.prampolini@pi.iccom.cnr.it; mariachiara.pastore@univ-lorraine.fr; fabrizio.santoro@pi.iccom.cnr.it

Contents

S1 Additional Computational Details **S3**

S1.1 Definition of intermolecular dimer coordinates S3

S1.2 The coupled diabatic States of the PDI dimer S4

S1.3 Spectral intensity and first moment S4

S1.4 Constrained dimer optimization S4

S1.5 MCTDH settings S5

S2 Additional Results **S8**

S2.1 Experimental spectra S8

S2.2 Dimer Static spectrum with a fully parametrized LVC Hamiltonian. S11

S2.3 Accuracy of the simplified Ad-MD|gLVC route S11

S2.4 MD Structural Analysis: lateral pendants S13

S2.5 MD Structural Analysis: aggregate shape in ACN S15

S2.6 MD Structural Analysis: aggregate shape in H_2O S19

S2.7 Parametric study of stacked dimers S20

S2.8 The prediction of different models for PDI monomer spectrum S22

S2.9 Further investigations on the origin of the lowest energy peak underestimation. A small two-states two-modes model S23

S2.10Comparison of the Ad-MD|LVC spectra for the PDI dimer in for the individual snapshots in ACN and water S28

S2.11Robustness of the estimate of the interstate couplings S29

S2.12Relation between aggregate structure and excited states couplings S30

S2.13Diabatic energies and inter-state couplings QD statistics S37

S2.14Absorption spectrum and population dynamics predicted by the averaged Hamiltonian S38

References **S40**

S1 Additional Computational Details

S1.1 Definition of intermolecular dimer coordinates

The intermolecular dimer coordinates employed in this work, and shown in Figure 3.a in the main text, are based on three unit vectors defined with respect of each PDI aromatic core. In fact, as displayed in Figure S1, for each core k, $\hat{u}_{||}$ lies parallel to the PDI core's long axis, $\hat{u}_{\perp}$ is the unit vector normal to the aromatic plane and $\hat{u}_S$ is obtained by the vector product between the first two. According to these molecular reference

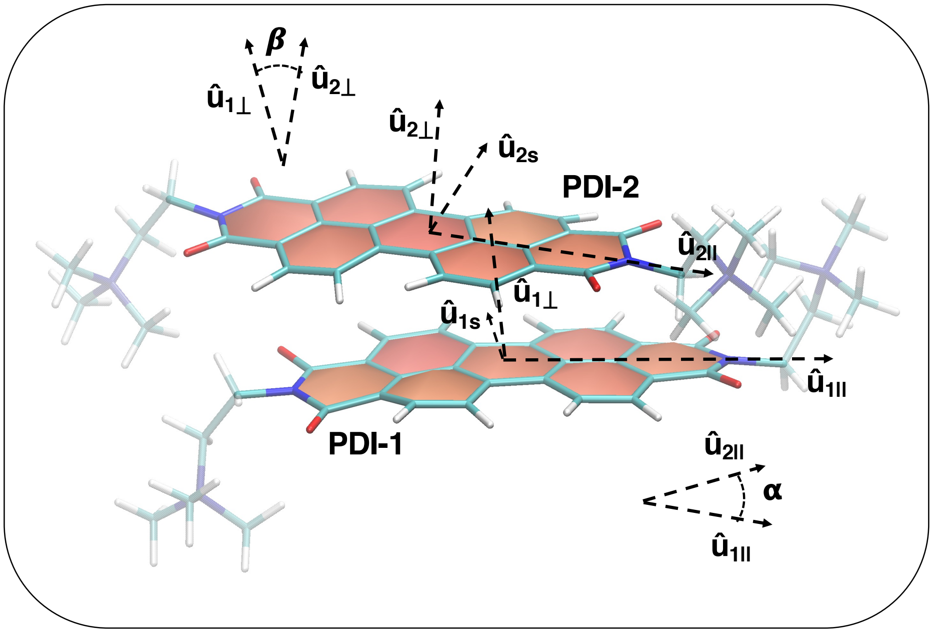

Figure S1: Definition of unit vectors on each monomer.

systems, the intermolecular coordinates shown in Figure 3 can be defined as follows:

$$\rho = \vec{R}_{||} \cdot \hat{u}_{1||} + \vec{R}_{\perp} \cdot \hat{u}_{1\perp} + \vec{R}_s \cdot \hat{u}_{1s} \tag{1}$$

$$\alpha = \hat{u}_{1||} \cdot \hat{u}_{2||} \tag{2}$$

$$\beta = \hat{u}_{1\perp} \cdot \hat{u}_{2\perp} \tag{3}$$

S1.2 The coupled diabatic States of the PDI dimer

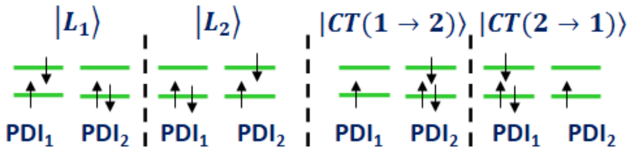

Figure S2: A cartoon representing the the four coupled diabatic states defined for the PDI dimer in terms of the individual monomers. The two green lines indicate the HOMO (lower line) and LUMO (upper line) of each monomer, and the arrows represent the electrons with their spin.

S1.3 Spectral intensity and first moment

Analytical expressions for the total intensity (moment 0, M_0) can be straightforwardly obtained considering the absorption lineshape, $\epsilon(\omega)/\omega$ neglecting the prefactor $\mathcal{C}_\epsilon$, and integrating over the frequency.

$$I_{ABS} = M_0 = \sum_i |\boldsymbol{\mu}_{gi}|^2 = 2|\boldsymbol{\mu}_{g,L}|^2 \tag{4}$$

where $\boldsymbol{\mu}_{g,L}$ is the transition dipole of the diabatic states representing the local excitations. The first moment, i.e. the center of gravity of the spectrum is

$$M_1 = M_0^{-1} \sum_{i,j} \boldsymbol{\mu}_{gi}\boldsymbol{\mu}_{jg} \langle d_i|H|d_j\rangle \tag{5}$$

S1.4 Constrained dimer optimization

According to the original Ad-MD|gVH scheme,[S1] the reference geometry in the reduced space of the fast coordinates, $\mathbf{q}_o^r$, is computed performing an harmonic extrapolation from the gradient and Hessian matrix. Here, since in the simplified route of the Ad-MD|LVC scheme specific normal modes for each snapshot are not needed, the time consuming computation of the Hessian matrices is not longer required, and $\mathbf{q}_o^r$ can be retrieved simply by re-optimizing the PDI core of each monomer. However $\mathbf{q}_o^{r,\alpha}$ is still needed to perform the single-point FrD-Ex diabatization. Concretely, to retrieve $\mathbf{q}_o^{r,\alpha}$ for each MD snapshot α, we carried out a DFT geometry re-optimization for each individual monomer, where the atoms pertaining to its lateral pendants and to the other PDI monomer were constrained, hence only the atoms of one PDI core were allowed to relax. An ONIOM QM/MM partition was followed, where the surrounding chemical species located within a radius of 4 Å (ACN, Cl^-, and the other PDI) are included in the DFT optimization at MM

level (employing QMD-FF point charges and LJ terms), whereas all the chemical species within a radius of 20 Å are instead treated as QMD-FF point charges. Although approximated, this is the easiest procedure to perform the required constrained optimization in the space of the stiff coordinates only. Finally, for each snapshot, the optimized geometry of the dimer is straightforward obtained by combining the two optimized monomers.

In principle, to have a more accurate structure, the aforementioned re-optimization could be carried out considering both PDI monomers at the QM level, hence including the effect of the mutual polarization. Yet, to avoid to alter the soft intermolecular coordinates describing the relative disposition of the PDI cores sampled by MD, several constraints between the PDI cores should be applied, with a significant impact on the computational cost. In order to verify the small impact of neglecting such mutual polarization, we compared the structures of the PDI dimer for 4 snapshots (2 extracted from the MD of the *anti* isomers and 2 from the one of the *syn* isomers) obtained by including the second PDI monomer at either MM or QM level. The differences between the two structures were found extremely limited, being the averaged Root Mean Square Deviation (RMSD) between them of the order of 0.06 Å. To further corroborate the validity of the approximation, we performed TD-DFT calculations and adiabatic-to-diabatic transformation considering both the constraint optimization geometries and the fully-relaxed ones, to eventually compute the distance between the diabatic Hamiltonian matrices utilizing the Frobenius distance:

$$d^F_{AB} = \sqrt{Tr((A-B)*(A-B)')} \tag{6}$$

The distances thus calculated have been averaged for the 4 snapshots considered in this analysis obtaining very small both distance and standard deviation: 0.063 ± 0.016 eV, and hence validating our procedure.

S1.5 MCTDH settings

Nuclear wavepackets were propagated for 100 fs in steps of 0.5 fs. For the parametric study and the dimer minimum calculation, we used a combination of a 7th order Bulirsch-Stoer integrator with a stepsize of $2.5{\cdot}10^{-4}$ and a 5th order SIL integrator, both with an accuracy of 10^5. The mean field matrices were assumed constant during the time interval (Constant Mean Field, CMF), also with an accuracy of 10^5. We used effective modes generated with a hierarchical representation of the Hamiltonian. For the convergence of the spectrum broadened with a Gaussian with HWHM=0.05 eV 2 blocks were necessary (8 coordinates when only λ_{ii} coupling are included, 12 for λ_{ii} and λ_{ij}). For the calculation of the spectra of each snapshot we adopted a narrower broadening with HWHM=0.03 eV. Therefore the effective modes of one additional hierarchy blocks were required, for a total of 12 modes. In these cases we used the Multi-Layer extension

ML-MCTDH with a 7th order Runge-Kutta integration and the mean field matrices were updated at each integration step (Variable Mean Field, VMF). The same ML-MCTDH settings were used for the computation of the time-evolution of the populations.

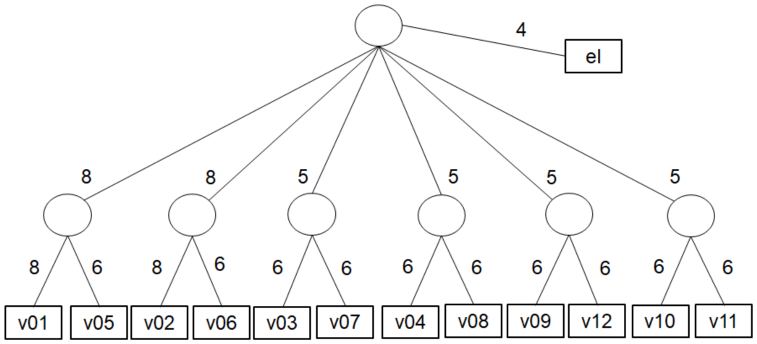

Figure S3: Combination of degrees of freedom for the MCTDH calculations of the full LVC Hamiltonian, including all $\boldsymbol{\lambda_{ii}}$ and the linear couplings $\boldsymbol{\lambda_{12}}$ and $\boldsymbol{\lambda_{34}}$. All $E_{ij}(0)$ terms were included. We adopted 2 hierarchy blocks for a total number of 12 coordinates.

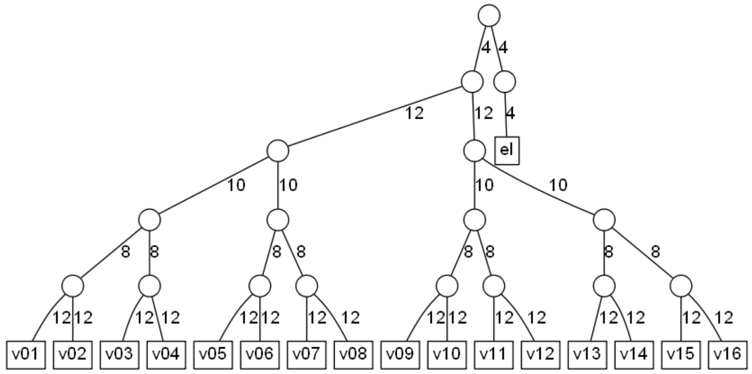

Figure S4: ML-MCTDH tree used of the simplified route employed for the snapshot calculations, where the LVC Hamiltonian only includes the diagonal gradients $\boldsymbol{\lambda_{ii}}$ and all the constant terms $E_{ij}(0)$. We adopted 4 hierarchy blocks for a total number of 16 coordinates for testing, 3 blocks for narrow spectra (HWHM = 0.03eV) and 2 hierarchy blocks for a total number of 8 coordinates for broader spectra (HWHM=0.05eV). The tree for 2 hierarchy blocks was obtained by suppressing the right branch of coordinates, while on the tree for 3 blocks, the right branch of coordinates was suppressed the coordinates were combined in groups of 3 on the lowermost level.

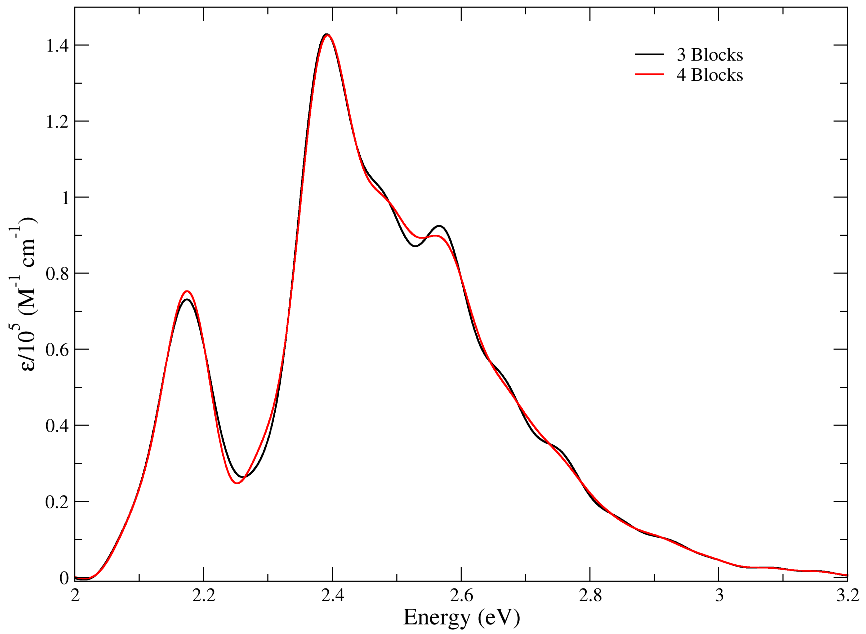

Figure S5: Convergence test for the number of hierarchical blocks. Spectra obtained using the LVC gradients $\boldsymbol{\lambda}_{ii}$ and the constant coupling terms $E_{ij}(0)$ calculated for the minimum of the PDI dimer. The spectra were redshifted by 0.25eV and convoluted with a Gaussian with HWHM = 0.03eV. The calculations with 3 and 4 blocks include respectively 12 and 16 coordinates.

S2 Additional Results

S2.1 Experimental spectra

Difference spectra were obtained by subtracting, from the initial spectrum of PDI in pure acetonitrile, the subsequent spectra in the presence of increasing water concentration (Figure S7) or the spectrum obtained by adding 20μl of the saturated solution on PDI in ACN to water solution (Figure S9). In order to do so, each spectrum was normalized between 0 and 1 by using the lowest wavelength absorption peak and by subtracting the background baseline between 600 and 800 nm. In fact, the spectral baseline progressively increased upon the addition of water due to some light scattering caused by aggregate formation.

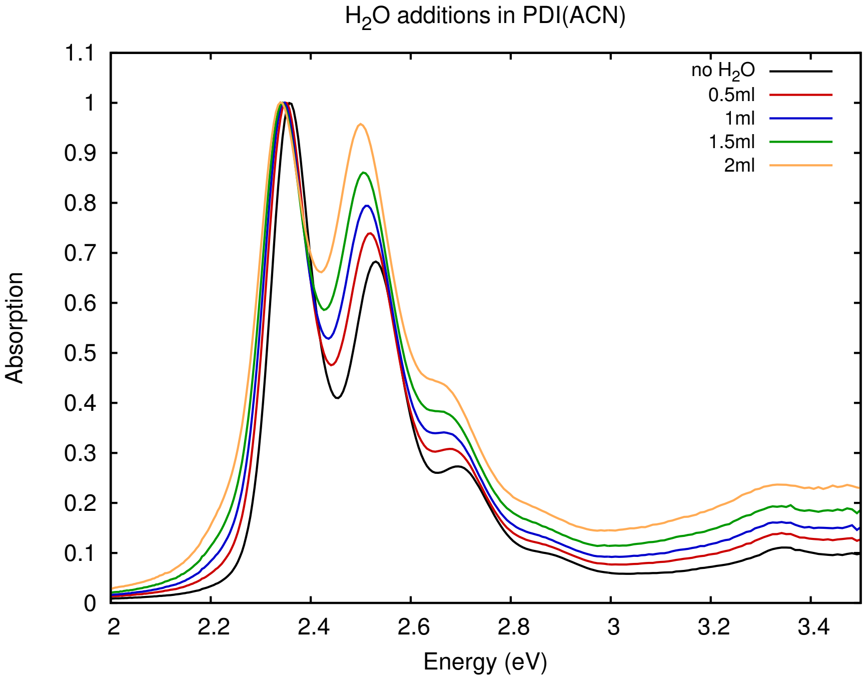

Figure S6: Normalized absorption spectrum registered for a saturated 2 ml solution of PDI in ACN (black line), and normalized absorption spectra obtained by adding to the original solution an increasing quantity of water (from 0.5 ml up to 2 ml).

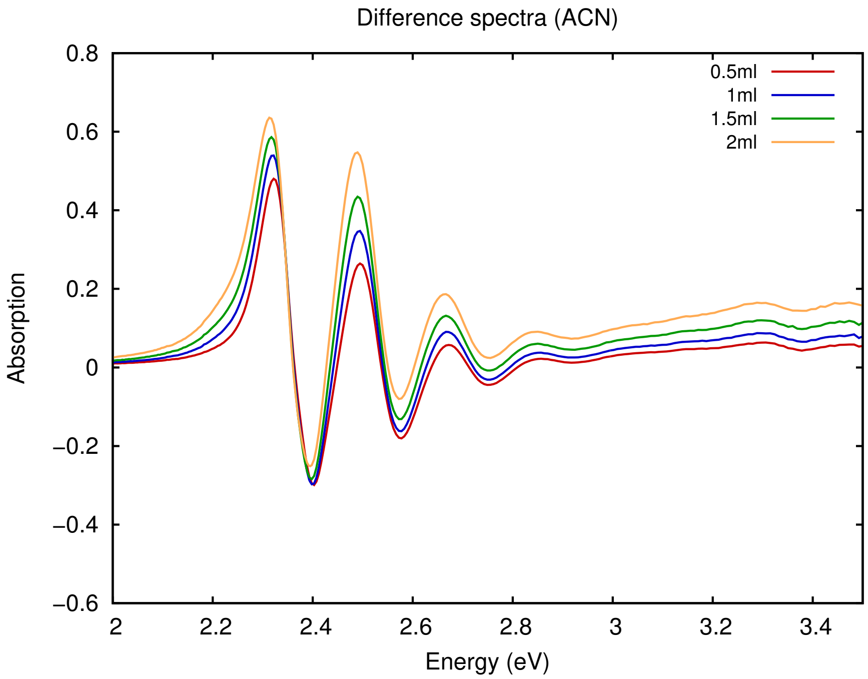

Figure S7: Difference between the spectra registered for PDI(ACN) in the presence of different amount of water (0.5 ml - 2 ml) and the spectrum registered for the saturated solution of PDI in ACN (black line in Figure S6).

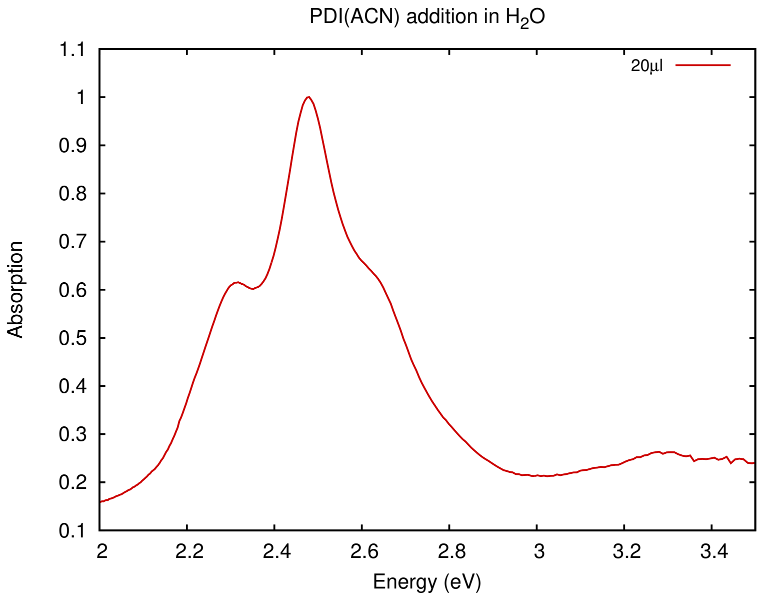

Figure S8: Normalized absorption spectra registered from a solution of water and 20μl of the saturated solution of PDI(ACN).

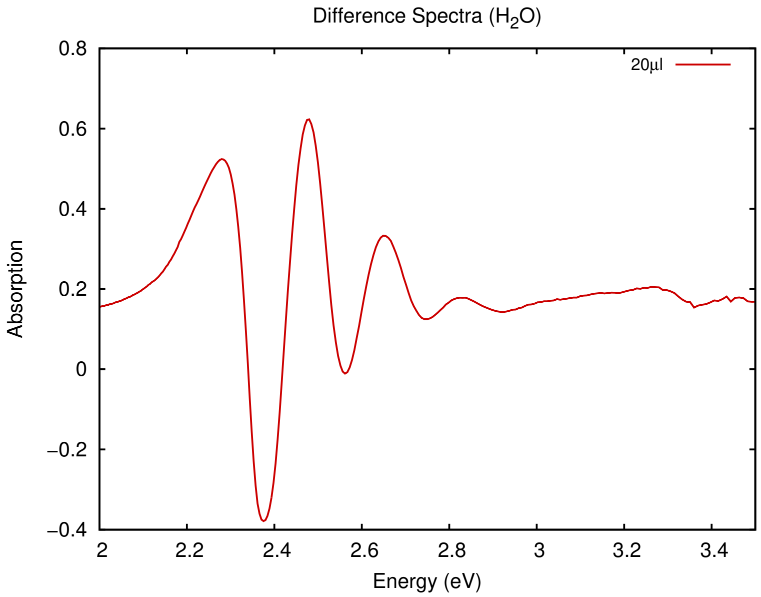

Figure S9: Difference between the spectrum registered when 20μl of the PDI(ACN) solution is added in water and the spectrum registered for the saturated solution of PDI in ACN (black line in Figure S6).

S2.2 Dimer Static spectrum with a fully parametrized LVC Hamiltonian.

As discussed in the main text, the full LVC protocol was employed to perform a static computation of the vibronic spectrum in ACN solution. With the term "static" we mean that we neglected all the effects of the fluctuations of the soft coordinates and we computed the spectrum considering a single reference geometry: the equilibrium geometry of the dimer in the *anti* conformation, obtained by a full QM optimization in ACN (C-PCM[S2]). The coordinates of the flexible lateral chains, i.e. all bonds, angles and dihedrals related to atoms in the chain, which are weakly involved in the electronic transition, were projected out of the coordinate space, following the iterative procedure introduced in previous works.[S1,S3] The inter-monomer coordinates, defined adopting the translation-rotation-internal coordinate (TRIC) system introduced by Wang and Song,[S4] were also projected out using the same protocol. This reduces the coordinate space from 450 normal coordinates to 224. The LVC parameters of the reduced dimensionality system were thereafter computed as described in the Computational Details. This step is computationally costly as it requires 449 TD-DFT computations of the dimer (**152** atoms).

Thereafter, the absorption spectrum was eventually computed adopting a hierarchical representation by blocks of the Hamiltonian.[S5–S9] Two different sets of collective coordinates were tested for the dimer minimum. The first set included all the relevant LVC parameters (gradients and linear coupling) while the second contained only the gradients. Figure S3 (2 blocks, 12 collective coordinates) and Figure S4 (2/4 blocks, 8/16 collective coordinates) give details on the adopted ML-MCTDH tree. We also tested the convergence of the spectra with respect to the number of hierarchical blocks in Figure S5. Both spectra are very similar and for larger broadening they are identical, however, for HWHM=0.03eV 3 blocks do not fully converge the high energy region and 4 blocks most be used.

S2.3 Accuracy of the simplified Ad-MD|gLVC route

In this section we test the accuracy of the approximations that allow us to speed up the full computational protocol presented in the left panel of Figure 2, leading to the simplified approach shown in the right panel of the same Figure. To this end, Figure S10 shows the results of a static computation of the vibronic spectrum in ACN solution. The computed spectrum is reported in black in Figure S10, and is only marginally different from a spectrum computed with $\boldsymbol{\lambda}_{ij} = \mathbf{0}$ (green line), proving that this approximation does not introduce any significant error. This optimal result is not surprising considering that the constant coupling terms E_{ij} are much larger than the norm of the vector of the corresponding linear terms $|\boldsymbol{\lambda}_{ij}|$, as evident from Table A. It is also noteworthy that, with this simplification, the number of coordinates corresponding to two blocks of the hierarchy is smaller (8) since it is not necessary to include coordinates parallel to the $\boldsymbol{\lambda}_{ij}$, making

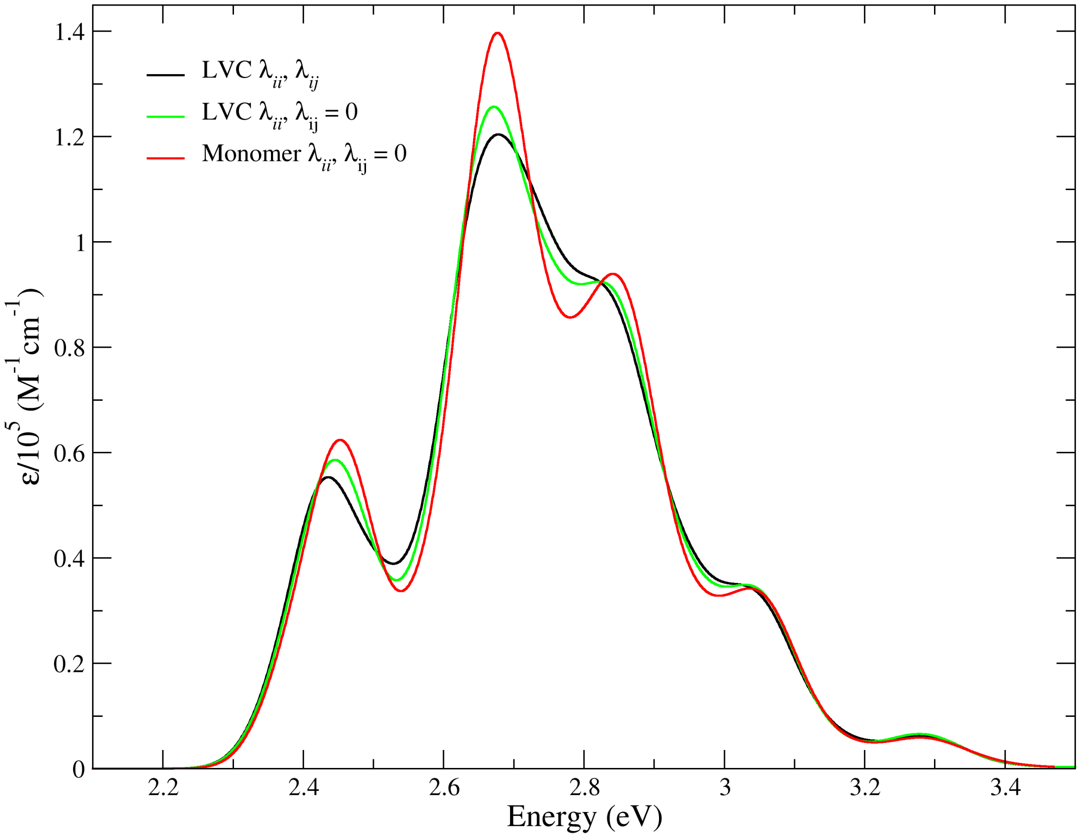

Figure S10: Calculated spectra obtained by including in the Hamiltonian i) all λ_{ii} and the most relevant LVC linear coupling parameters λ_{ij} (specifically λ_{LE-LE} and λ_{CT-CT}, ii) only the LVC gradients (λ_{ii}) or iii) using the gradients of the monomer local excitation and cationic and anionic states. In all cases, the constant coupling $E_{ij}(0)$ are included. All stick transition were convoluted with a Gaussian of HWHM = 0.05eV.

the MCTDH propagations faster. This is especially relevant for those snapshots where calculations with 4 blocks are required for high-resolution spectra: omitting $\boldsymbol{\lambda}_{ij}$ reduces the dimensions of the vibrational space from 24 to 16.

We also tested the second approximation proposed in the right panel of Figure 2, namely that the diagonal gradients $\boldsymbol{\lambda}_{ii}$ may be computed on monomers, rather than the dimer. Despite this crude approximation,

Table A: Constant coupling in the FC point, $E_{ij}(0)$, and sum of the magnitudes of the linear coupling parameters $|\lambda_{ij}(\alpha)|$, both in eV, for the minimum of the PDI dimer.

i	j	$E_{ij}(0)$ (eV)	$\Sigma_\alpha|\lambda_{ij}(\alpha)|$ (eV)
1	2	0.092	0.025
1	3	0.052	0.032
1	4	0.031	0.029
2	3	0.031	0.030
2	4	0.052	0.032
3	4	0.002	0.022

the shape of the computed spectrum (red line) is quite similar to the one of obtained with the accurate parametrization of the LVC Hamiltonian, showing that the gradients of the states are quite insensitive to the precise dimer arrangement and to the presence of the solvent. The latter result is not surprising since some of us recently showed that the shapes of the vibronic spectrum of the PDI monomer computed in gas and in ACN are extremely similar.[S3] The only appreciable difference is that the vibronic peaks of the approximated spectrum appear slightly more resolved, but this effect is then washed out by the average over a representative set of configurations of the dimer.

S2.4 MD Structural Analysis: lateral pendants

Figures S11-S13 show the distribution and the time evolution of the dihedral angle defining the rotation of the lateral pendants with respect to the π-plane of the PDI core ($\delta 1$). To highlight the rotation around $\delta 1$, and therefore the inter-conversion from the anti/anti dimer to the syn/anti one, we defined the starting value of this dihedral angle as always positive. Such definition allows us to easily recognize the *anti* $\rightarrow$ *syn* transition because, when this occurs, $\delta 1$ assumes negative values.

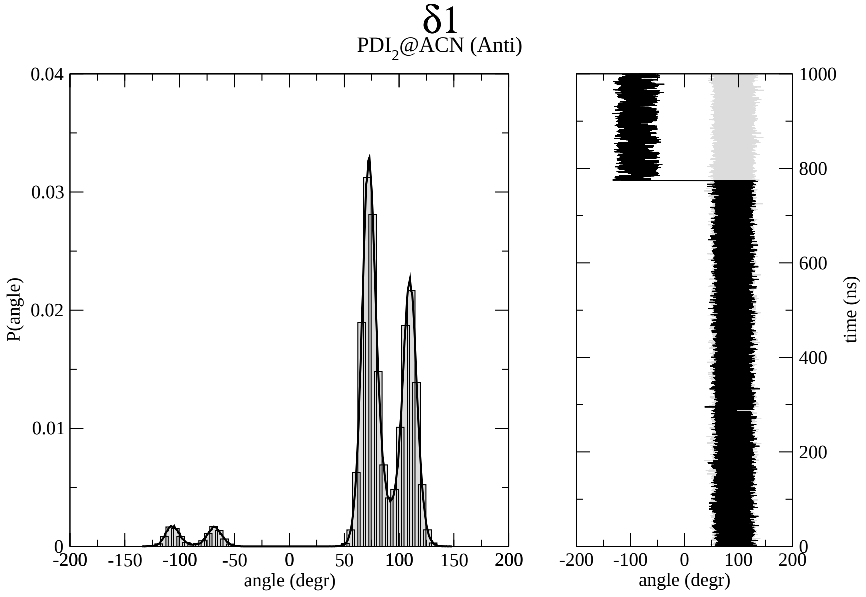

Figure S11: Distribution (left panel) and time series (right panel) of $\delta 1$ computed along the MD trajectories of the PDI_2@ACN starting with the *anti* isomers. In the right panel, the $\delta 1$ highlighted in black shows the lateral pendant rotating 180°.

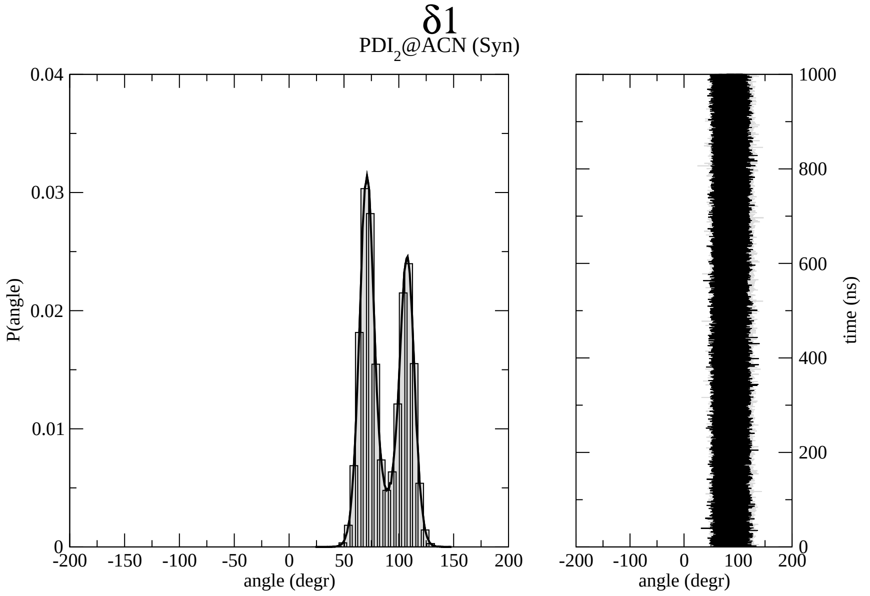

Figure S12: Distribution (left panel) and time series (right panel) of $\delta 1$ computed along the MD trajectories of the PDI_2@ACN starting with the *syn* isomers.

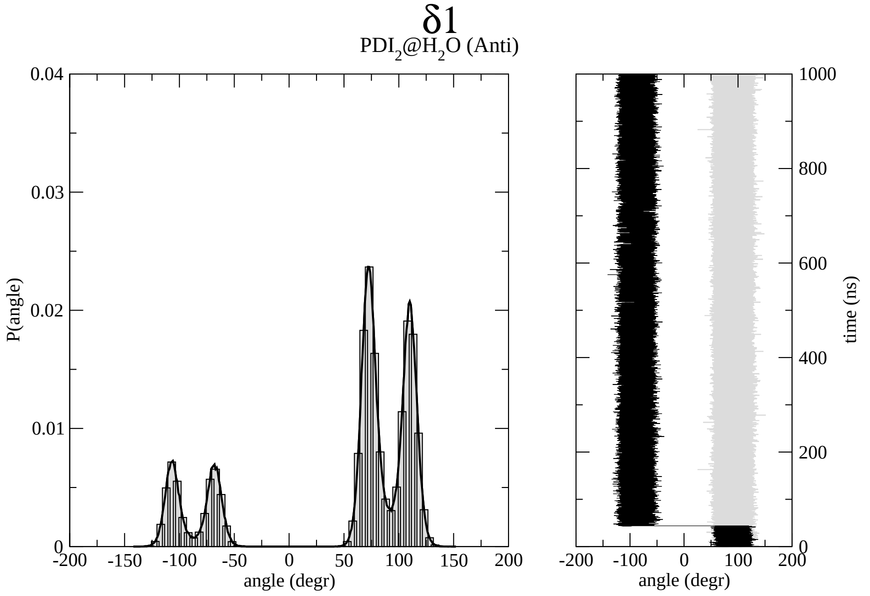

Figure S13: Distribution (left panel) and time series (right panel) of $\delta 1$ computed along the MD trajectories of the PDI_2@H_2O starting with the *anti* isomers. In the right panel, the $\delta 1$ highlighted in black shows the lateral pendant rotating 180°.

S2.5 MD Structural Analysis: aggregate shape in ACN

Figure S14 shows the time evolution of the distance between the center of the PDI cores (see ρ in Equation S1 and in Figure 3 of the main text). This Figure shows the tendency of PDI to form self-assembled dimers and has been computed along the MD simulations of both the isomers (syn and anti) for the PDI_2@ACN system. The 2D colour plots (Figures S14-S20) display the cross-correlation of the distribution of the intermolecular geometrical parameters analyzed in this work. The color scale reported on the right hand side of these graphs represents the frequency with which it is populated a PDI_2 structure having the intermolecular geometrical characteristics displayed in the X and Y axes. Also in this case, as for Figure S14, the MD simulations have been analyzed of both isomers of the PDI_2@ACN system.

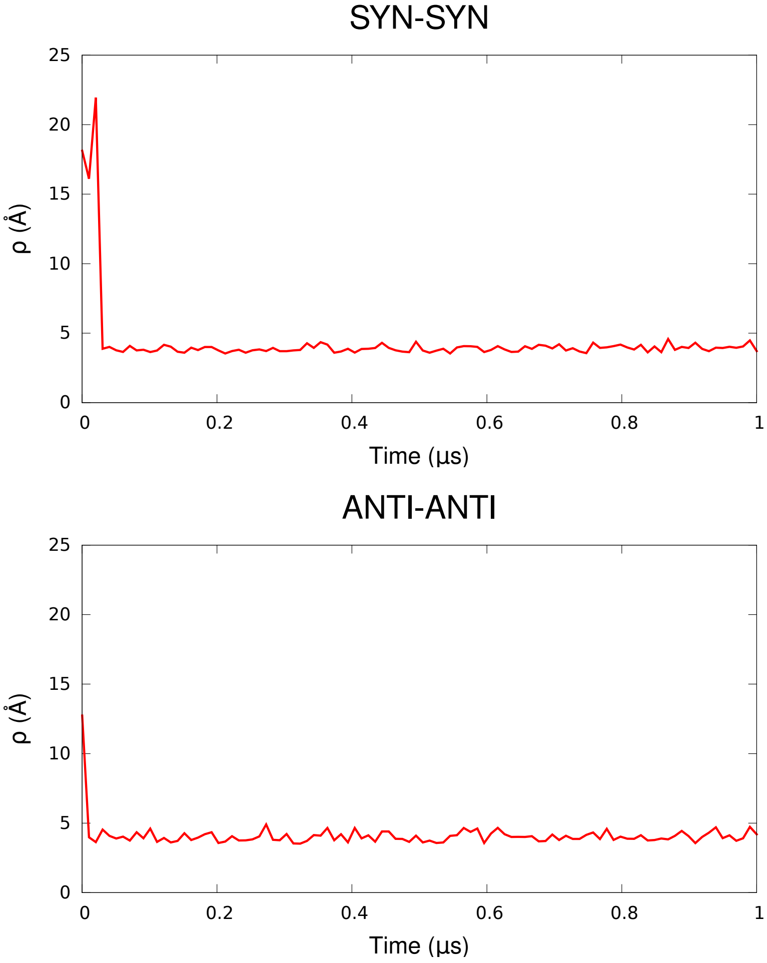

Figure S14: Time evolution of the intermolecular distance ρ(Å) for the two MD runs starting with the two PDI monomers in either *syn* (top panel) or *anti* (bottom panel) conformation.

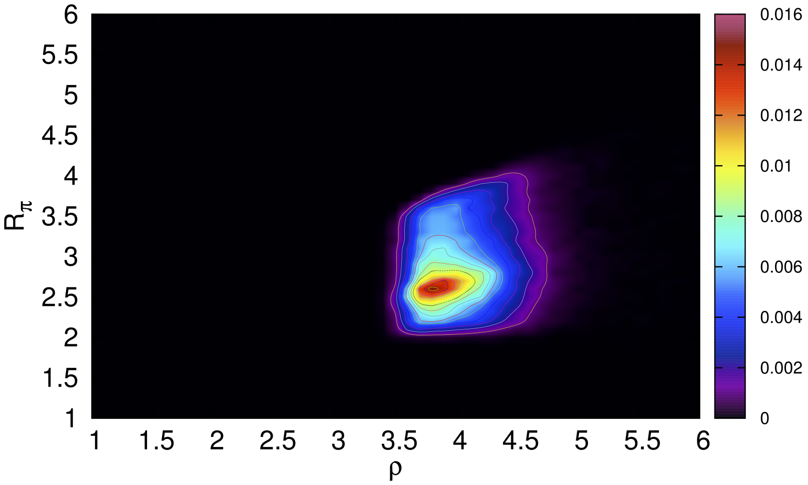

Figure S15: 2D colour plot correlating the distribution of ρ(Å) and of R_π(Å) computed along MD simulations starting with the *anti* isomers. The monitored PDI's intermolecular IC are displayed in Figure 3 and Figure S1.

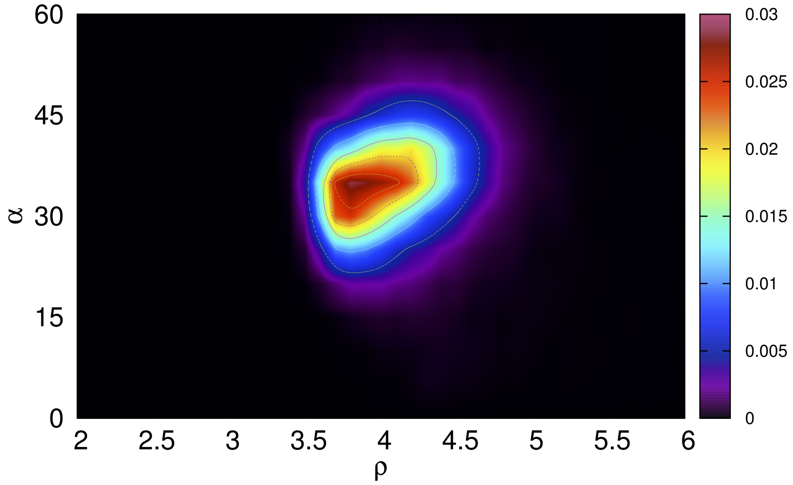

Figure S16: 2D colour plot correlating the distribution of ρ(Å) and of α(degrees) computed along MD simulations starting with the *anti* isomers. The monitored PDI's intermolecular IC are displayed in Figure 3 and Figure S1.

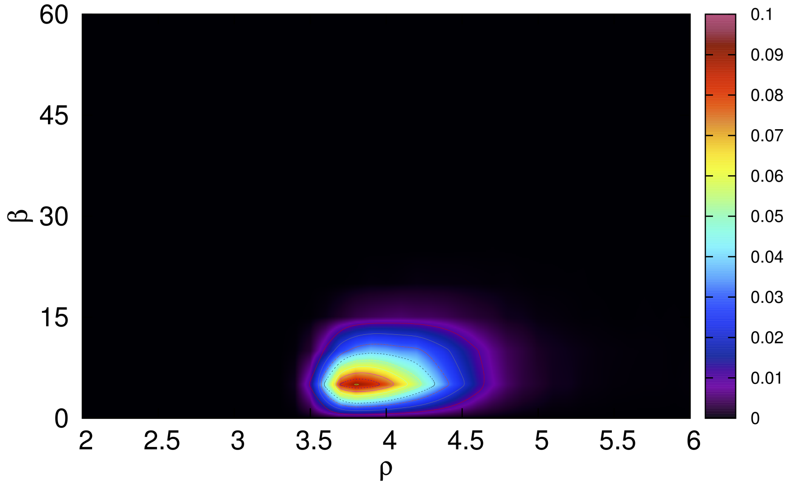

Figure S17: ρ-β 2D colour plot correlating the distribution of ρ(Å) and of β(degrees) computed along MD simulations starting with the *anti* isomers. The monitored PDI's intermolecular IC are displayed in Figure 3 and Figure S1.

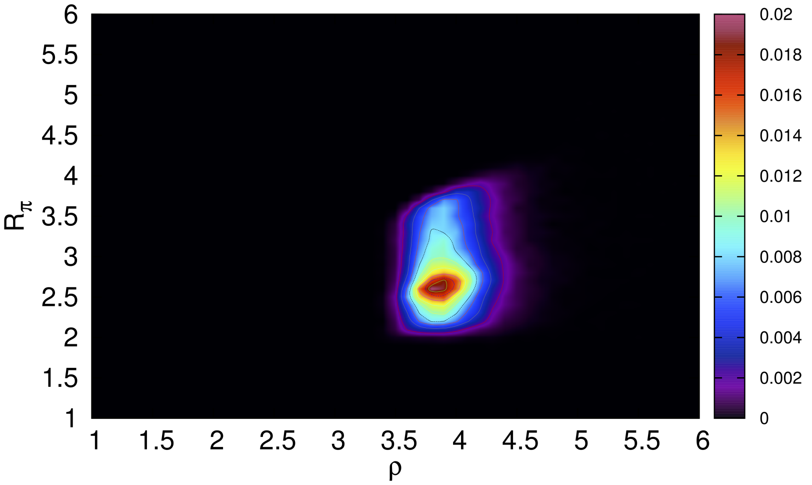

Figure S18: 2D colour plot correlating the distribution of ρ(Å) and of R_π(Å) computed along MD simulations starting with the *syn* isomers. The monitored PDI's intermolecular IC are displayed in Figure 3 and Figure S1.

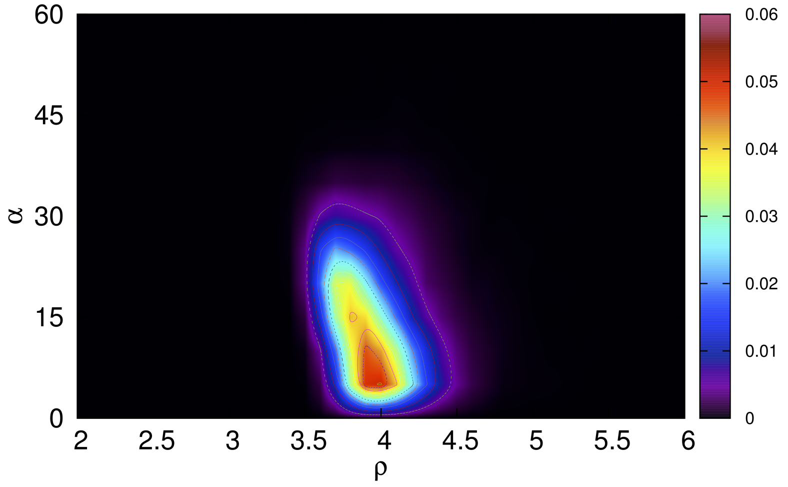

Figure S19: 2D colour plot correlating the distribution of ρ(Å) and of α(degrees) computed along MD simulations starting with the *syn* isomers. The monitored PDI's intermolecular IC are displayed in Figure 3 and Figure S1.

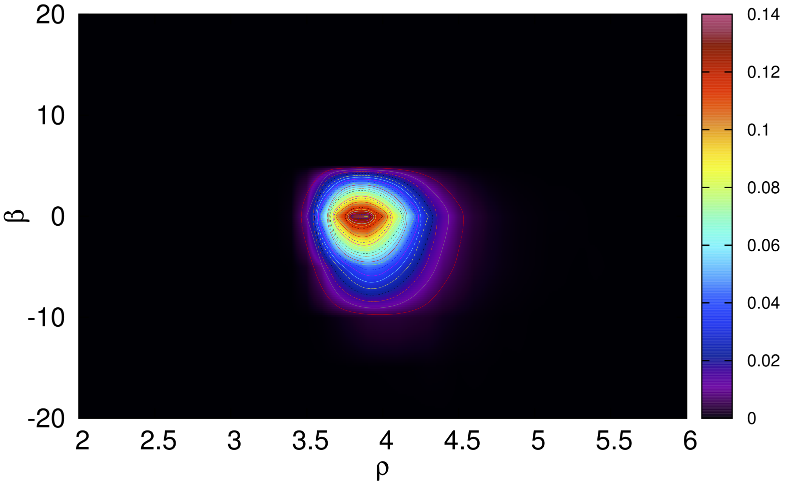

Figure S20: 2D colour plot correlating the distribution of ρ(Å) and of β(degrees) computed along MD simulations starting with the*syn* isomers. The monitored PDI's intermolecular IC are displayed in Figure 3 and Figure S1.

S2.6 MD Structural Analysis: aggregate shape in H_2O

Figure S21 shows the distributions of the intermolecular descriptors used to analyze the dimer shape in ACN reported in Figure 4 of the main text. Recalling that the anti isomers only have been used for the MD simulations in water and that the *anti* $\rightarrow$ *syn* transition is faster than in ACN (see Figures S11-S13), the distributions of Figure S21 do not differ substantially from those computed for the corresponding simulations in ACN (Figure 4a). The only difference worth mentioning concerns the β angle distribution that shows smaller values in Figure S21, demonstrating the tendency of PDI to form dimers with more parallel planes in aqueous solutions.

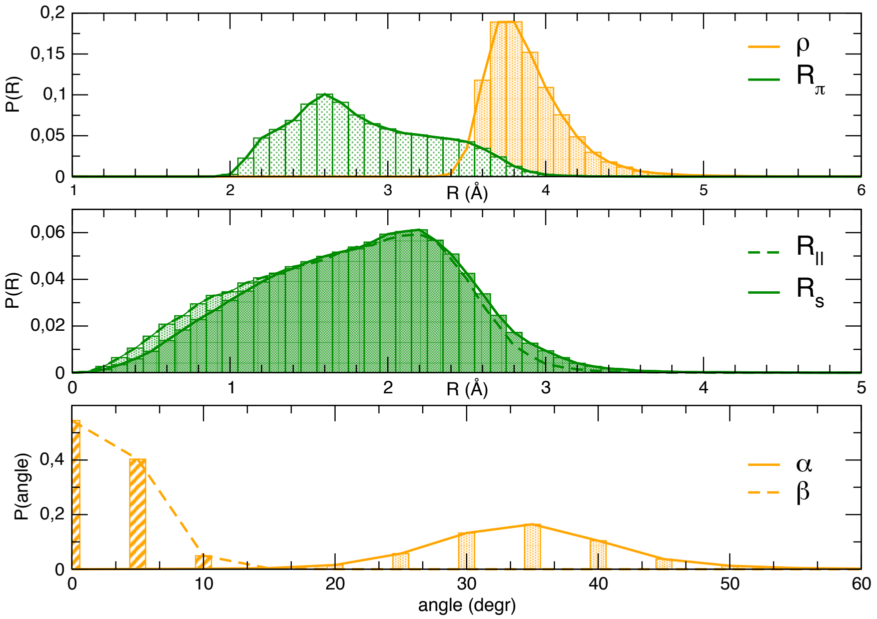

Figure S21: Distributions of inter-molecular (PDI-PDI) angles, distances, and displacements as defined in Figure 3 and Figure S1 computed along the MD trajectories of the $PDI_2@H_2O$ starting with the *anti* isomer.

S2.7 Parametric study of stacked dimers

Figure S22 complements Figure 5 in the main text. While for the sake of brevity, in the latter figure we only reported the energy gap between the CT and LE states, here we plot the absolute energies of the four diabatic states. It is interesting to notice that LE energies change only very moderately. On the contrary CT energies decrease remarkably with the three distances and in particular with R_π and then R_S. They are insensitive to α and more sensitive to the two β angles, which also induce a difference in energy between the two CT states.

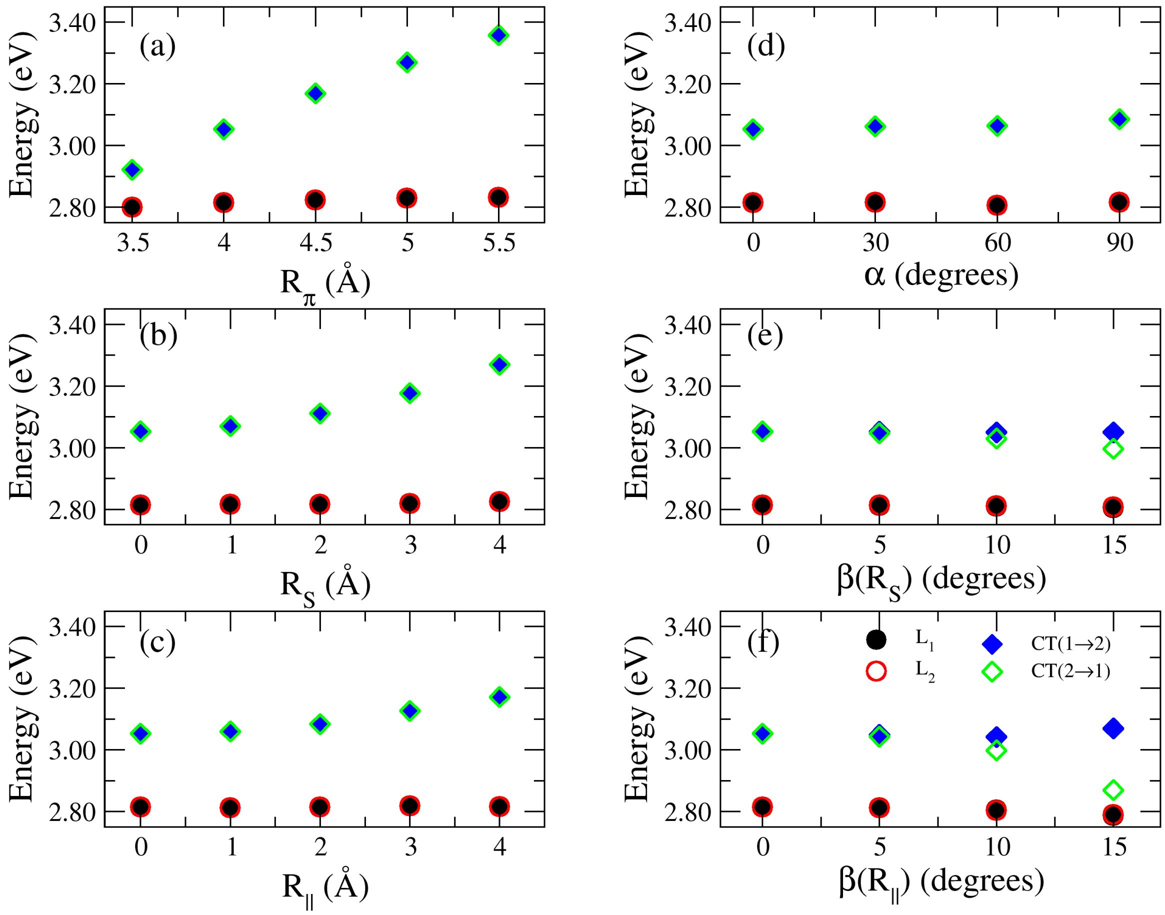

Figure S22: Vertical energies for the diabatic states of the PDI dimer without pendants and in gas phase for different structural parameters.

Figure S23 shows the absorption spectra computed for the dimer configurations investigated in the parametric study. Notice that spectra were red-shifted by the same amount (0.27 eV) adopted to plot the spectra in Figure 6 in the main text. It is evident that R decreases upon decreasing either one of the three considered distances, or α. In all these cases, these movements correspond to an increase of the exciton coupling. On the contrary, the spectral shape seems rather insensitive to $\beta(R_S)$, whereas large tilting around $\beta(R_{\|})$ lead to a number of weak bands on the red wing of the spectrum.

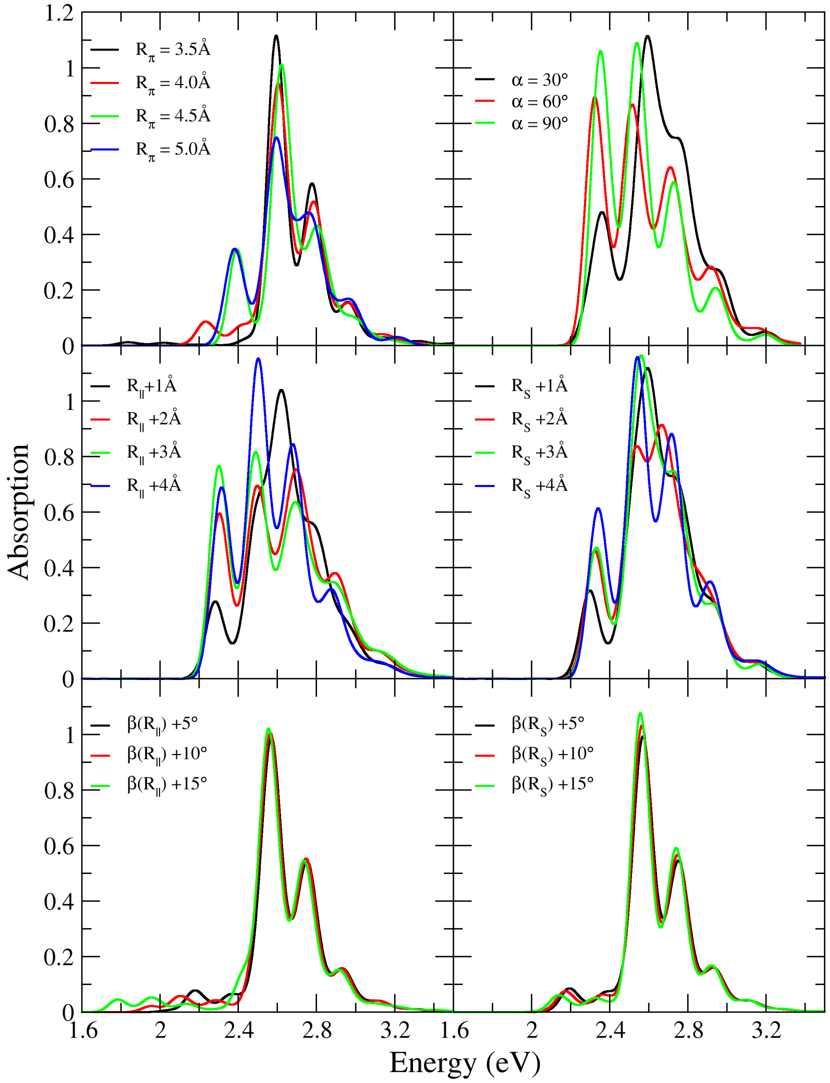

Figure S23: Spectra of the PDI dimer without pendants and in gas phase for different structural parameters. All calculated spectra were redshifted by 0.27eV and arbitrarily scaled so to fit in the same figures. We applied the same scaling factor to all spectra in the same panel, but such factor can vary from panel to panel. Spectra convoluted with a Gaussian of HWHM = 0.05 eV.

S2.8 The prediction of different models for PDI monomer spectrum

It should be noticed that the monomer spectrum reported in Figure 6 in the main text is slightly different from the Ad-MD|gVH one given in Ref. S3. Indeed, to be coherent with the approximations in the LVC model, here we neglected quadratic differences in the PESs from the ground to the excited states, i.e. Duschinsky rotation and changes in the vibrational frequencies, replacing the VH model by the simpler VG one.[S10] Ad-MD|gVH and Ad-MD|gVG are compared in Figure S24, showing that such quadratic corrections (neglected also in the spectra of the dimer) have a very slight impact on the spectral shape and simply cause a red-shift by ~ 0.08 eV.

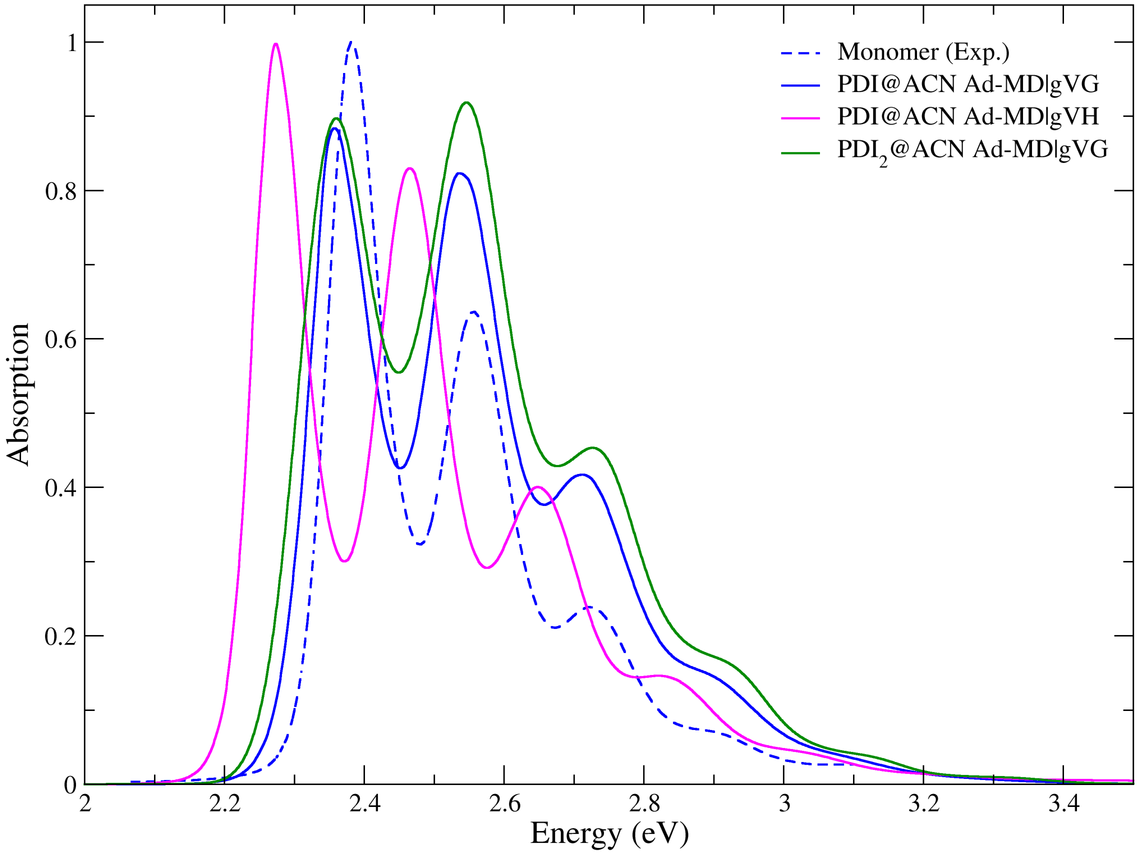

Figure S24: Comparison of experimental and calculated spectra for the PDI monomer. The calculated spectra were obtained either from the dimer snapshots, setting all inter-state couplings to zero (PDI_2@ACN Ad-Md|gVG) or by applying the Ad-MD|gVH protocol whose data are reproduced from a previous publication,[S3] or, finally, still adopting the same electronic data of ref.[S3] but employing the simpler Ad-MD|gVG protocol. The latter model, like the LVC one, assumes that all excited electronic states have the same normal modes and frequencies of the ground state. All calculated spectra were redshifted by 0.27eV and arbitrarily scaled by the same factor. The stick transitions were convoluted with a Gaussian of HWHM=0.05eV.

Figure S24 also compares the monomer spectrum with Ad-MD|VG and with Ad-MD|gLVC, setting to zero all inter-state couplings (PDI_2@ACN Ad-MD|gVG). This is equivalent to compute the spectrum of one monomer in a solvation shell comprising also the second monomer. The position of the spectrum is practically

the same, showing that the effect of one PDI on the vertical excitation of the other monomer is very small. The intensity of the second peak is moderately larger for the Ad-MDg|LVC, and this finding is attributed to the effect of the replacement of the pendants by H atoms (notice that a similar effect was appreciated in Figure S10). Comparison with the experiment shows that all the computed spectra overestimates the ratio R and the larger discrepancy is observed for the "PDI_2@ACN Ad-MD|gVG" calculation. As discussed in the main text, this suggests that the simplified model adopted to compute the gradients of the LE states can contribute to the excessive splitting of the lowest two bands in the dimer spectrum.

S2.9 Further investigations on the origin of the lowest energy peak underestimation. A small two-states two-modes model

To gain insight on the origin of the spectral bands, we performed time-independent calculations of the spectra on a fully eclipsed PDI dimer (R_π = 4.0 Å). In this framework, including the nonadiabatic effects is very computational demanding so we used a very reduced model for the dimer composed of 2 electronic states (the two LE) and two normal coordinates (the one carrying the largest gradient for each state). In addition, the constant interstate coupling $E_{12}(0)$ was also included in the model while the linear vibronic coupling terms $\lambda_{ij}(k)$ were neglected, like it is done in the simplified route adopted in the paper. Moreover the coupling to the CT states is neglected. The analysis of Figure 7 shows that it has a very limited impact on the spectral shape. Whereas the two states are the two local excitations $|L_1\rangle$ and $|L_2\rangle$, the two considered coordinates are the first members of the hierarchy, i.e. the coordinates q_1 and q_2 describing the displacement of the L_1 and L_2 equilibrium geometries with respect to the ground state geometry.

First, we check convergence by comparing with the spectrum obtained for the same model in the time-dependent framework for which convergence is easily achieved. Figure S25 shows that the TI spectrum agrees very well with the TD one so we can confidently assign the spectral bands from this model.

Figure S26 compares the predictions of the two-states two-coordinates model (2Q) with those of the model adopted in the main text and involving all the normal coordinates (All Q). We report both non-adiabatic spectra (LVC) of the dimer and the diabatic limit obtained switching off the excitonic coupling and corresponding to the monomer spectrum (FC|VG). Focusing first on the monomer spectra, the figure shows that the "2Q" model reproduces the general shape of the spectrum obtained including all coordinates, but for a relevant overestimation of the ratio R. Switching on the excitonic coupling, the 2Q model nicely captures the change of the spectrum from the monomer to the dimer predicted by the "All Q" model, thus reproducing the main experimental observations. Like for the monomer case, also for LVC nonadiabatic spectra of the dimer the "2Q" model predicts larger R than the "All Q" model.

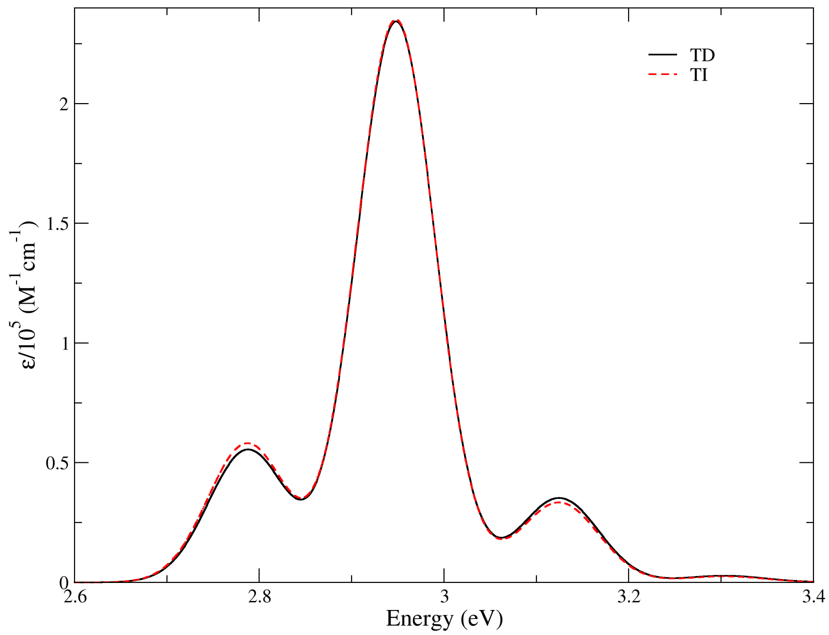

Figure S25: Comparison of the calculated LVC time-independent and time-dependent spectra for a reduced dimensionality model including 2 normal coordinates and 2 excited states. Stacking distance of 4Å and relative rotation angle $\alpha = 0°$. For TD approach, both auto and cross terms were included in the computation of the spectrum.

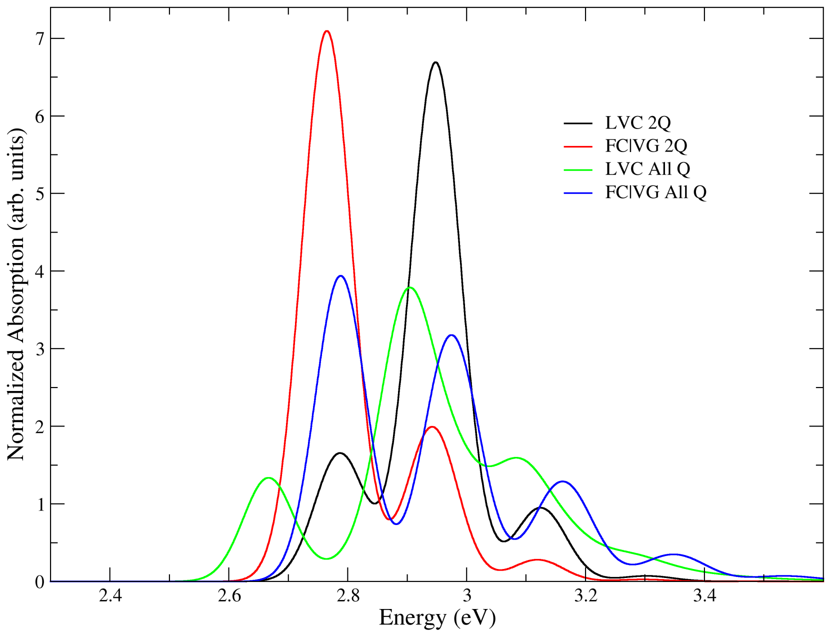

Figure S26: Calculated LVC spectra for the two-states-two-coordinates simplified model and the all coordinates model turning on or off the interstate coupling between the local excitation. The CT states were excluded of the model. To simplify the comparison, all spectra have unit area. Stacking distance of 4Å and relative rotation angle $\alpha = 0°$.

After proving that the 2Q model capture the main features of the monomer and dimer spectra, we employ it to further analyse the role of the exciton coupling and the gradient of the LE state (which determines the displacement of its equilibrium). For such an analysis the 2Q model is quite helpful because it is so small to allow us the computation of the vibronic eigenstates whose assignment is straightforward introducing symmetry-adapted electronic states $|A_1\rangle = 1/\sqrt{2}(|L_1\rangle - |L_2\rangle)$, $|A_2\rangle = 1/\sqrt{2}(|L_1\rangle + |L_2\rangle)$ and coordinates $q_- = 1/\sqrt{2}(q_1 - q_2)$, $q_+ = 1/\sqrt{2}(q_1 + q_2)$. With reference to a perfectly cofacial arrangement, the symmetric $|A_2\rangle$ is higher in energy and bright, whereas the anti-symmetric one $|A_1\rangle$ is lower in energy and dark.

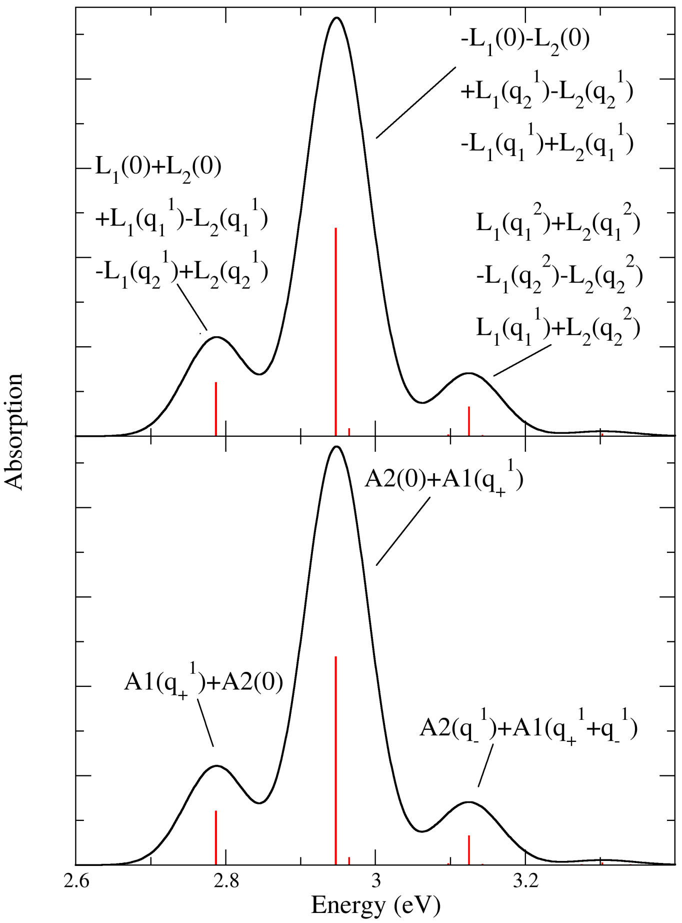

Figure S27: Calculated LVC time-independent spectra and assignment of the vibronic bands on the diabatic representation (top) and the symmetry-adapted adiabatic representation (bottom) for a two-states-two-coordinates simplified model. In the diabatic representation each label (1 and 2) correspond to one monomer, while for the symmetry-adapted adiabatic representation the states are ordered in increasing energy and since the excitation coupling is positive, S1 and S2 correspond to the antisymmetric and symemtric combination of the local excitations. The coordinates q_+ and q_- are respectively the symmetric and antisymmetric combinations of the monomer normal coordinates. "0" stands for the 0-0 vibronic transition. Stacking distance of 4Å and relative rotation angle $\alpha = 0°$. For this arrangement S1 is totally dark and S2 is bright.

Figure S27 shows the assignment of the three bands in terms of the normal coordinates on the monomers (i.e., q_1 and q_2, top panel) and also in terms of symmetry adapted coordinates $q_+ = q_1 + q_2$ and $q_- = q_1 - q_2$

(bottom). Before starting the analysis, it is worth stressing that the position of the bands of the 2Q spectrum does not correspond to the one of the spectra reported in Figure 6. This finding is due to a number of reasons (i) the fact that the spectra in Figure 6 were red-shifted by 0.27 eV, (ii) the different spatial arrangement of the two monomers (an average from MD snapshots in Figure 6, a cofacial arrangement with a distance 4Å here), (iii) the lack of the effect of the other coordinates. Therefore the 2Q spectrum has three bands at $\sim$ 2.78, 2.95 and 3.12 eV, which correspond to the three bands in Figure 6 at $\sim$ 2.3, 2.5 and 2.7 eV.

The lowest two bands of the spectrum (those at $\sim$ 2.78 and $\sim$ 2.95 eV, respectively) are combinations of the two states $|A_2, 0_-, 0_+\rangle$ (bright) and $|A_1, 1_-, 0_+\rangle$ (dark), where the first label specifies the electronic state and the second and third numbers give the quantum number of the vibrations q_- and q_+. The highest band at $\sim$ 3.12 eV arises from the maximum band with a progression along the total-symmetric mode q_+ and is a combination of $|A_2, 0_-, 1_+\rangle$ and $|A_1, 1_-, 1_+\rangle$. All the data for the assignment of the bands are given in Table B.

Table B: Contribution of vibronic states to the first (top), second (middle) and third (bottom) bands upon variation of $E_{12}(0)$ (third column, $\lambda_{12}(\mathbf{q})$ fixed at -0.13 eV) and the linear coupling term $\lambda_{12}(\mathbf{q})$ (last column with $E_{12}(0)$ fixed at 0.126 eV). The states are labelled as (e, n_{q_-},n_{q_+}) where e represents the adiabatic electronic state and n_{q_-},n_{q_+} respectively are the quanta on the coordinates q_-,q_+.

		$\lambda_{12}(\mathbf{q}_-)$=-0.13eV					$E_{12}(0)$=0.126eV				
		$E_{12}(0)$					$\lambda_{12}(\mathbf{q}_-)$				
vibro. state	q. numbers	0.086	0.106	0.126	0.146	0.166	-0.09	-0.11	-0.13	-0.15	-0.17
1	(A_1,0,1)	0.55	0.67	0.76	0.83	0.87	0.46	0.49	0.51	0.52	0.53
	(A_2,0,0)	0.42	0.29	0.20	0.14	0.10	0.52	0.49	0.46	0.44	0.42
2	(A_1,0,1)	0.37	0.26	0.18	0.12	0.09	0.50	0.45	0.40	0.35	0.30
	(A_2,0,0)	0.57	0.70	0.79	0.85	0.89	0.48	0.51	0.53	0.55	0.56
3	(A_1,1,1)	0.37	0.25	0.18	0.12	0.09	0.50	0.45	0.40	0.35	0.30
	(A_2,1,0)	0.57	0.70	0.79	0.85	0.89	0.48	0.51	0.53	0.55	0.56

Finally, a parametric study changing the exciton coupling E_{L_1,L_2} and the gradient λ along the two coordinates q_1 and q_2 displayed in Figure S28 shows that the ratio of the intensity of the central band at 2.95 eV with respect to the lowest-one at 2.78 eV increases at the increase of E_{L_1,L_2}. At variance, it decreases at the increase of λ, but this is an indirect effect due to the transfer of intensity to the third band at 3.12 eV. The splitting of the two lowest bands increases both with E_{L_1,L_2} and λ. This suggests that the disagreement in the relative intensity of these bands with respect to the experiment should be due to an overestimation of the exciton coupling. This latter contributed also to the excessive splitting of the two bands, in this case in combination with the overestimation of the displacement of the equilibrium positions of the local excitations (already noticed for the monomer).

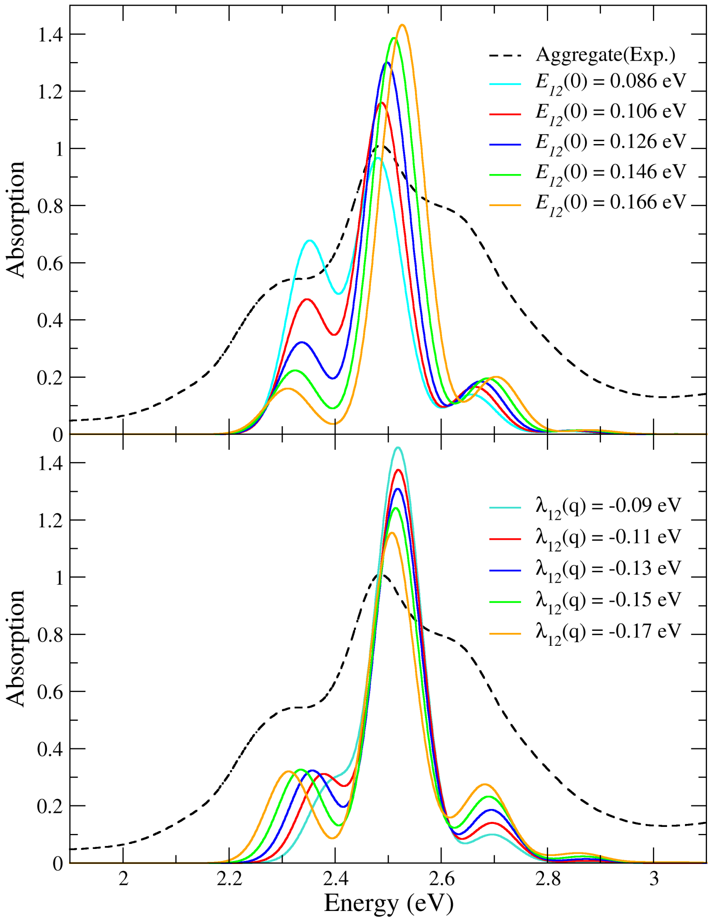

Figure S28: Calculated LVC time-independent spectra for a two-states-two-coordinates reduced dimensionality system with different values of the local excitation interstate constant coupling $E_{12}(0)$ (top, $\lambda_{12}(\mathbf{q}_-)$ fixed to -0.13eV) or the linear coupling $\lambda_{12}(\mathbf{q})$ (bottom $E_{12}(0)$ fixed to 0.126eV). Therefore, the blue spectrum is the same in both panels. All stick transitions were convoluted with a Gaussian of HWHM = 0.05eV and computed spectra were redshifted by 0.45eV. The experimental spectrum is added as reference. Stacking distance of 4Å and relative rotation angle α=0°.

S2.10 Comparison of the Ad-MD|LVC spectra for the PDI dimer in for the individual snapshots in ACN and water

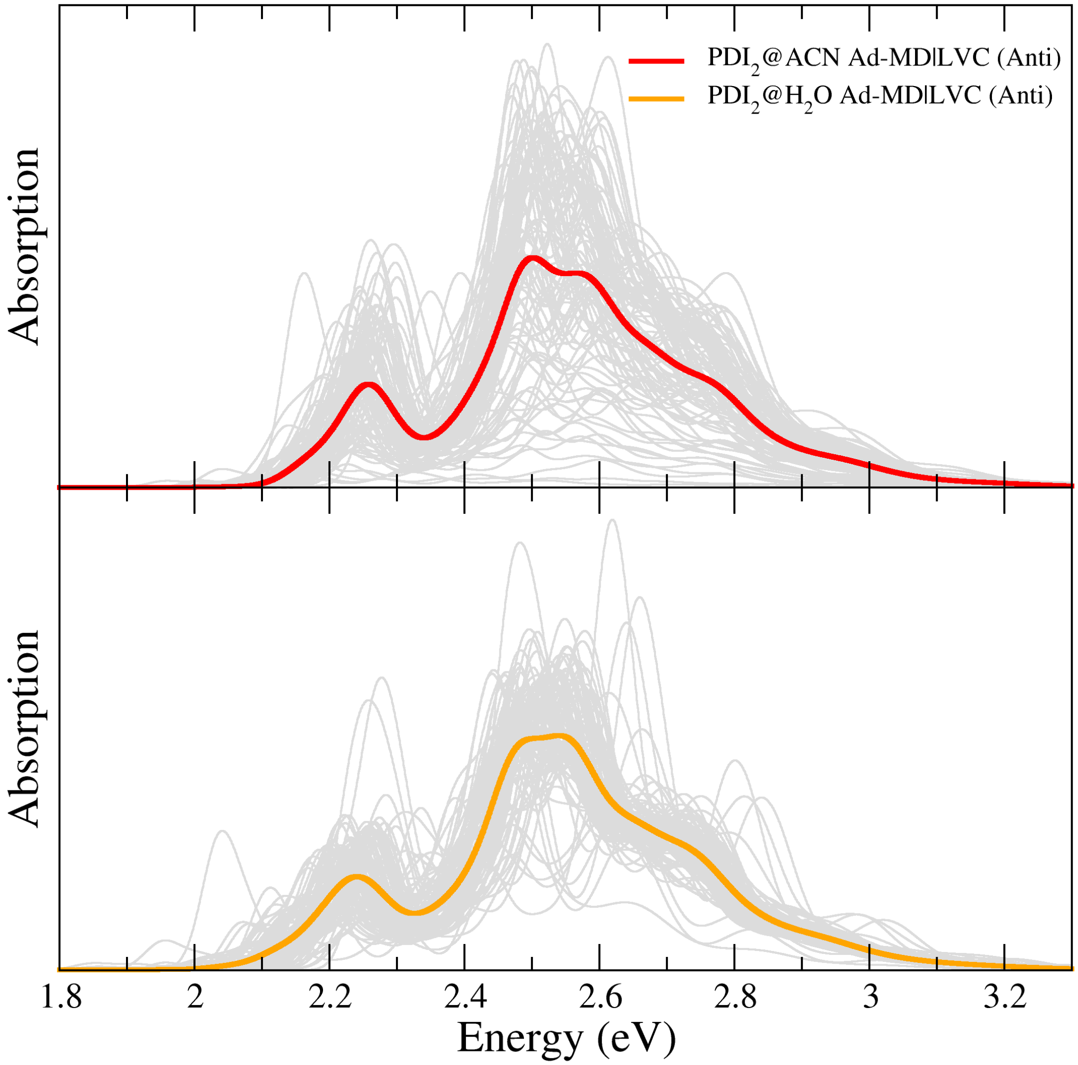

Figure S29: Ad-MD|LVC averaged spectrum and the individual contribution from each snapshot for PDI_2 anti conformer at ACN (top) and water (bottom). All stick transitions were convoluted with a Gaussian of HWHM = 0.03 eV.

S2.11 Robustness of the estimate of the interstate couplings

Table C: Interstate coupling, in eV, obtained with different functionals, all in combination with the 6-31G(d) basis set. PDI dimer with stacking distance of 4Å and relative rotational angle $\alpha = 0°$. For comparison the excitonic coupling between the two local excitations has been computed also from the Coloumb interaction betweeen transition densities (EET method in Gaussian 16, using the CAM-B3LYP functional[S11]). Labels for excited states are $1 = |L_1\rangle$, $2 = |L_2\rangle$, $3 = CT(1 \rightarrow 2)$ and $4 = CT(2 \rightarrow 1)$

Coupling	CAM-B3LYP	ωB97XD	M062X	PBE0	Coulomb(CAM)
$E_{21}(0)$	0.12606	0.12589	0.12425	0.11966	0.11916
$E_{31}(0)$	0.15926	0.15780	0.16371	0.14053	
$E_{32}(0)$	-0.14442	-0.14278	-0.14739	-0.12840	
$E_{41}(0)$	-0.14442	-0.14278	-0.14739	-0.12840	
$E_{42}(0)$	0.15926	0.15780	0.16371	0.14053	
$E_{43}(0)$	0.00005	-0.00046	0.00024	-0.00007	

S2.12 Relation between aggregate structure and excited states couplings

To have an accurate description of how the PDI dimer shape influences the exciton nature and delocalization, for all snapshots extracted from MD simulations in ACN and used to compute the spectra, we analyzed the Diabatic Hamiltonian matrices and displayed the relations between the geometrical descriptors used throughout the manuscript and the interstate couplings (off-diagonal terms of diabatic Hamiltonians) through 2D color maps. Whilst the color scales in Figures S15-S20 represent the probability of finding the PDI dimer in a specific geometrical configuration, the color scales in Figures S30-S41 represent the magnitude in eV of the interstate coupling between local excitations (LE-LE Coupling) and between the $|L_1\rangle$ and $|CT(1 \rightarrow 2)\rangle$ states (LE-CT Coupling). Although in the diabatic Hamiltonian there are four possible LE-CT couplings terms, these do not differ substantially in magnitude, therefore for the sake of simplicity, the coupling between $|L_1\rangle$ and $|CT(1 \rightarrow 2)\rangle$ only has been considered.

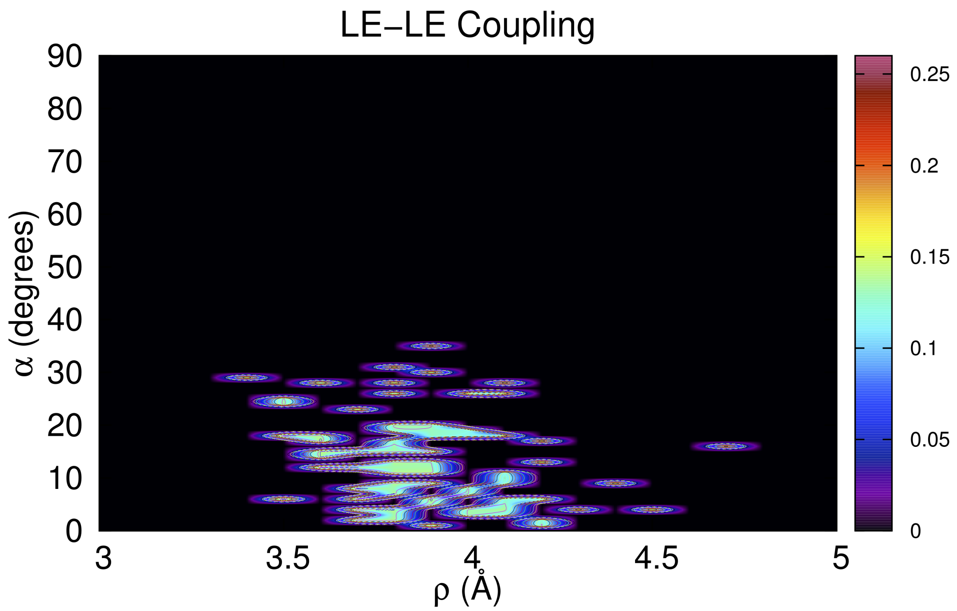

Figure S30: 2D heat map correlating the magnitude of the LE-LE coupling (colour palette in eV) with the values of ρ(Å) and α(degrees) assumed from the dimer. Snapshots extracted from the *syn* MD trajectory.

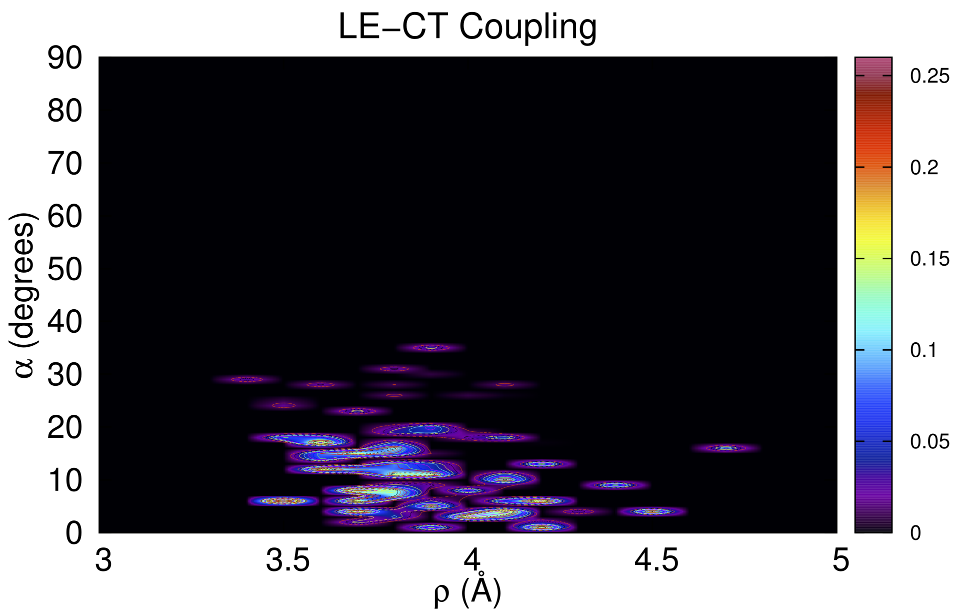

Figure S31: 2D heat map correlating the magnitude of the LE-CT coupling (colour palette in eV) with the values of ρ(Å) and α(degrees) assumed from the dimer. Snapshots extracted from the *syn* MD trajectory.

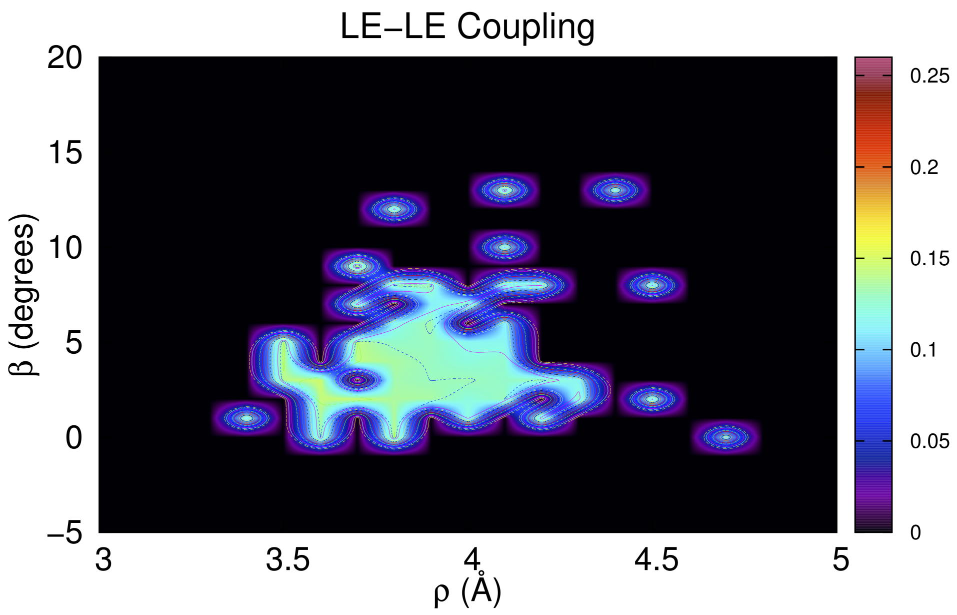

Figure S32: 2D heat map correlating the magnitude of the LE-LE coupling (colour palette in eV) with the values of ρ(Å) and β(degrees) assumed from the dimer. Snapshots extracted from the *syn* MD trajectory.

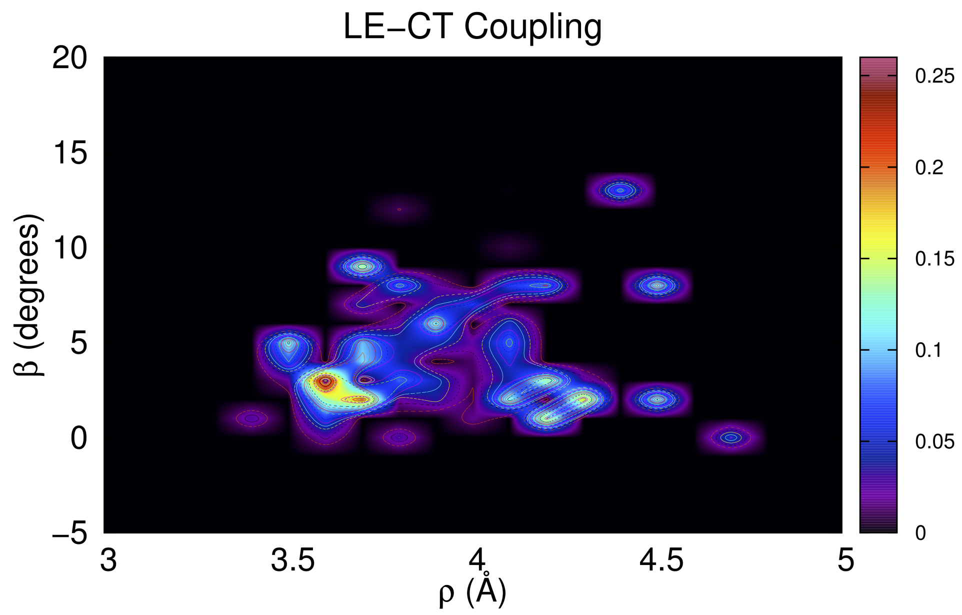

Figure S33: 2D heat map correlating the magnitude of the LE-CT coupling (colour palette in eV) with the values of ρ(Å) and β(degrees) assumed from the dimer. Snapshots extracted from the *syn* MD trajectory.

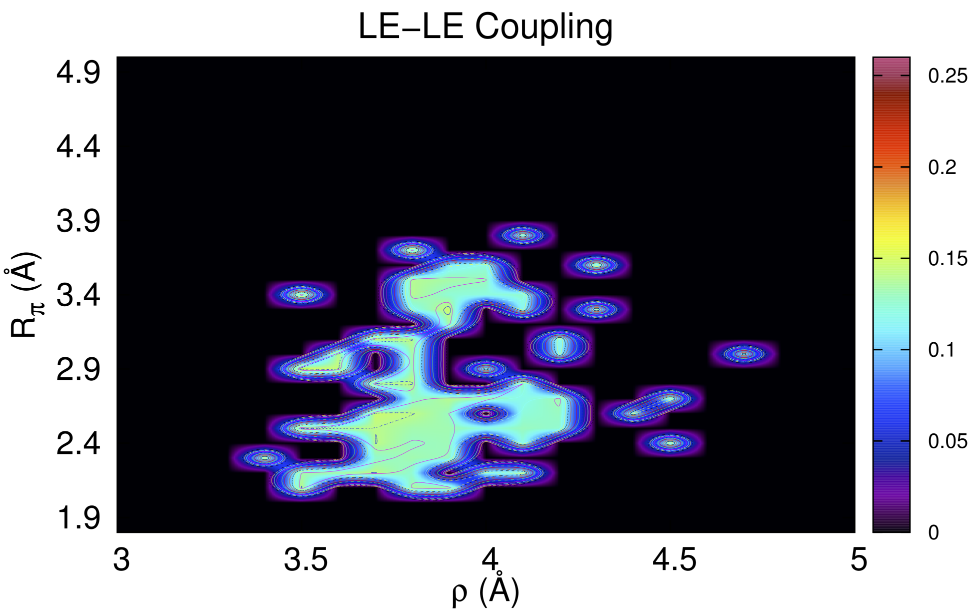

Figure S34: 2D heat map correlating the magnitude of the LE-LE coupling (colour palette in eV) with the values of ρ(Å) and R_π(Å) assumed from the dimer. Snapshots extracted from the *syn* MD trajectory.

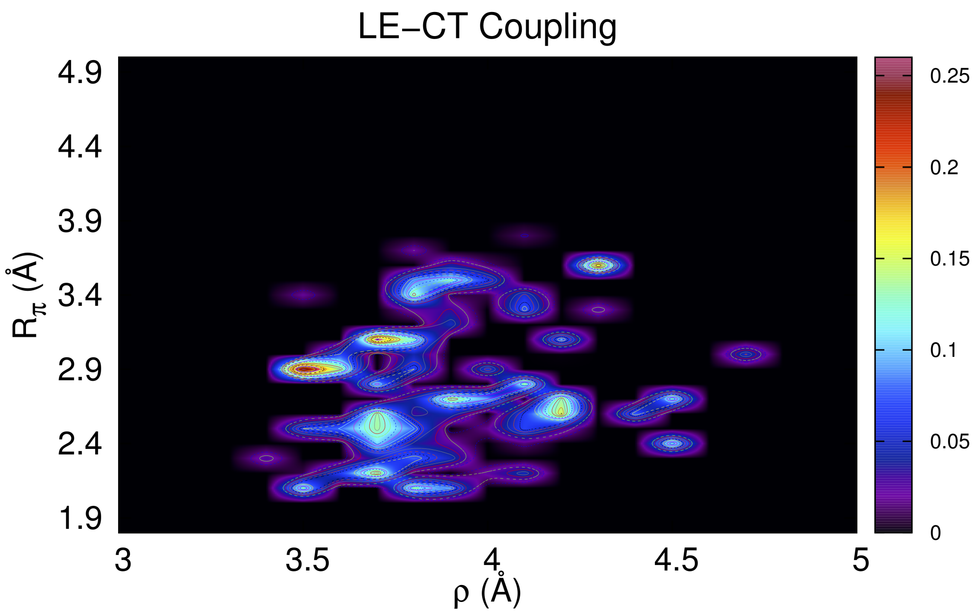

Figure S35: 2D heat map correlating the magnitude of the LE-LE coupling (colour palette in eV) with the values of ρ(Å) and R_π(Å) assumed from the dimer. Snapshots extracted from the *syn* MD trajectory.

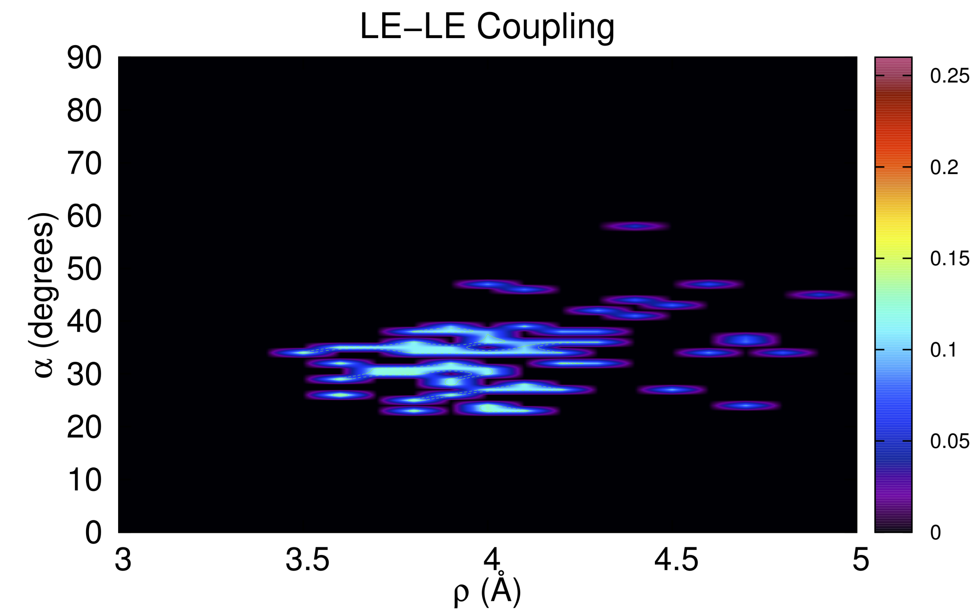

Figure S36: 2D heat map correlating the magnitude of the LE-LE coupling (colour palette in eV) with the values of ρ(Å) and α(degrees) assumed from the dimer. Snapshots extracted from the *anti* MD trajectory.

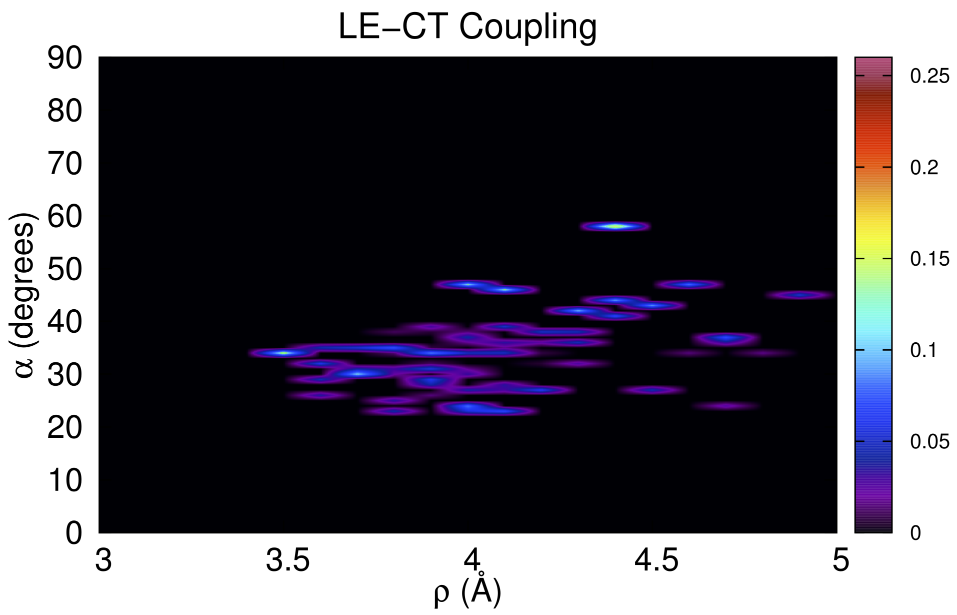

Figure S37: 2D heat map correlating the magnitude of the LE-CT coupling (colour palette in eV) with the values of ρ(Å) and α(degrees) assumed from the dimer. Snapshots extracted from the *anti* MD trajectory.

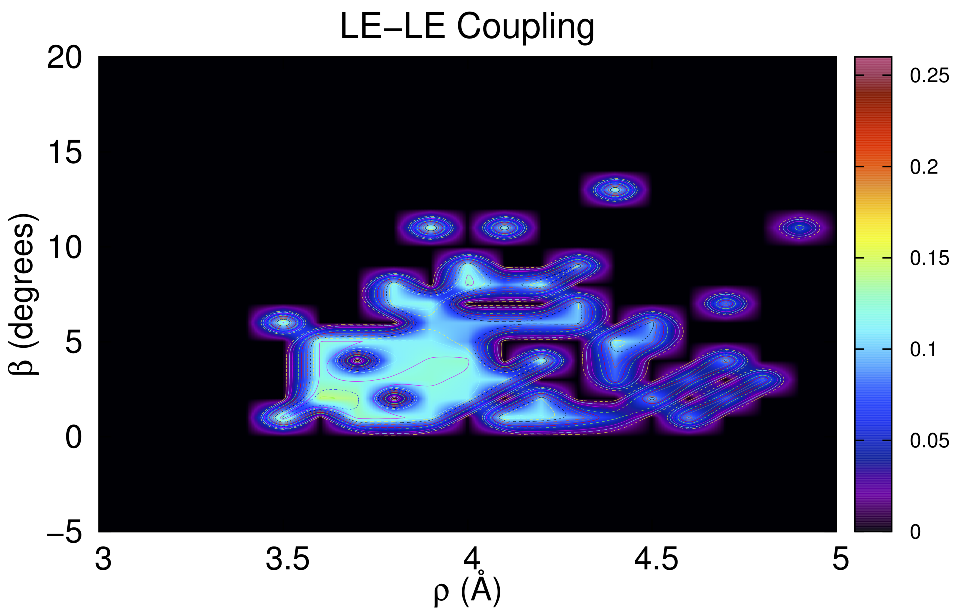

Figure S38: 2D heat map correlating the magnitude of the LE-LE coupling (colour palette in eV) with the values of ρ(Å) and β(degrees) assumed from the dimer. Snapshots extracted from the *anti* MD trajectory.

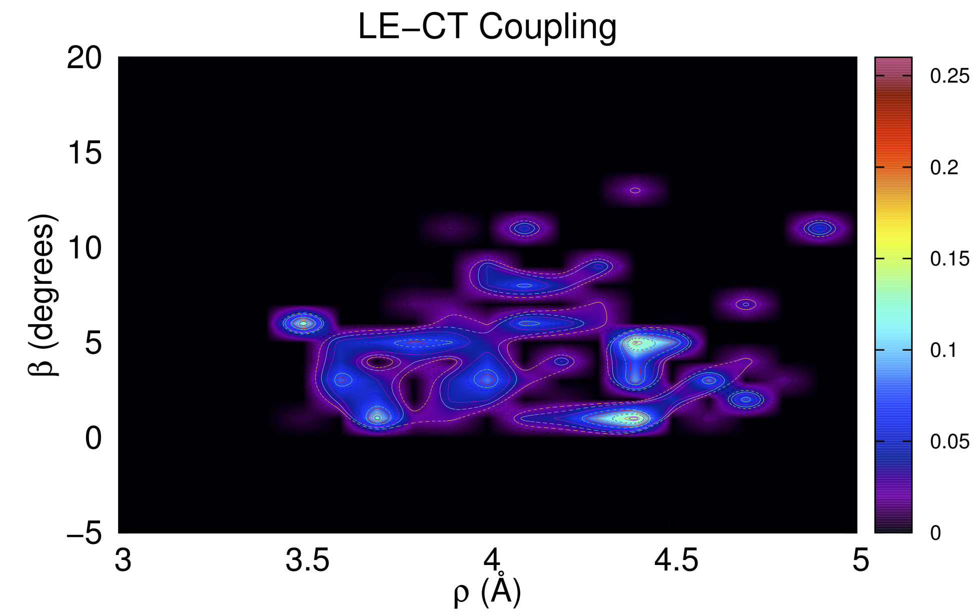

Figure S39: 2D heat map correlating the magnitude of the LE-CT coupling (colour palette in eV) with the values of ρ(Å) and β(degrees) assumed from the dimer. Snapshots extracted from the *anti* MD trajectory.

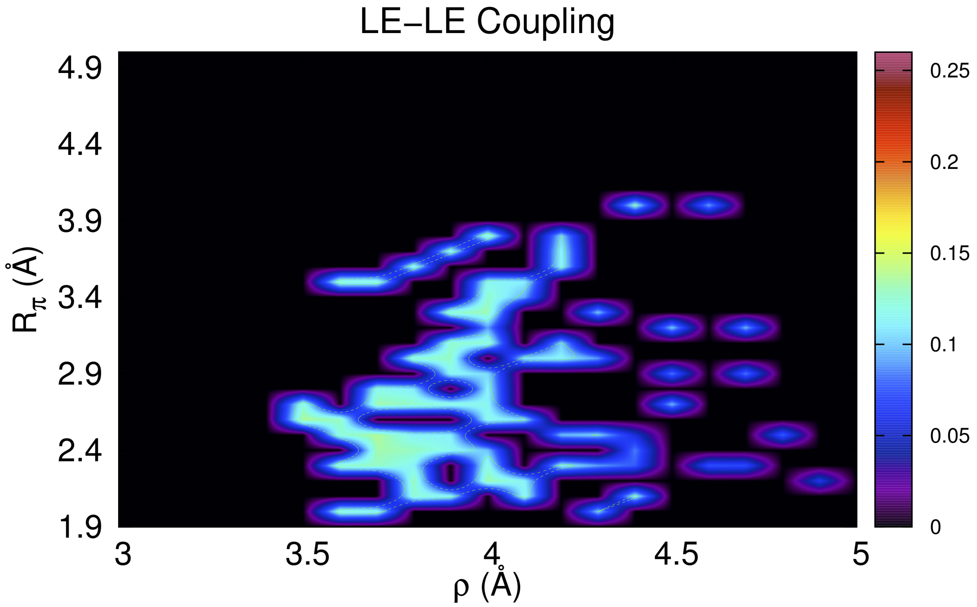

Figure S40: 2D heat map correlating the magnitude of the LE-LE coupling (colour palette in eV) with the values of ρ(Å) and R_π(Å) assumed from the dimer. Snapshots extracted from the *anti* MD trajectory.

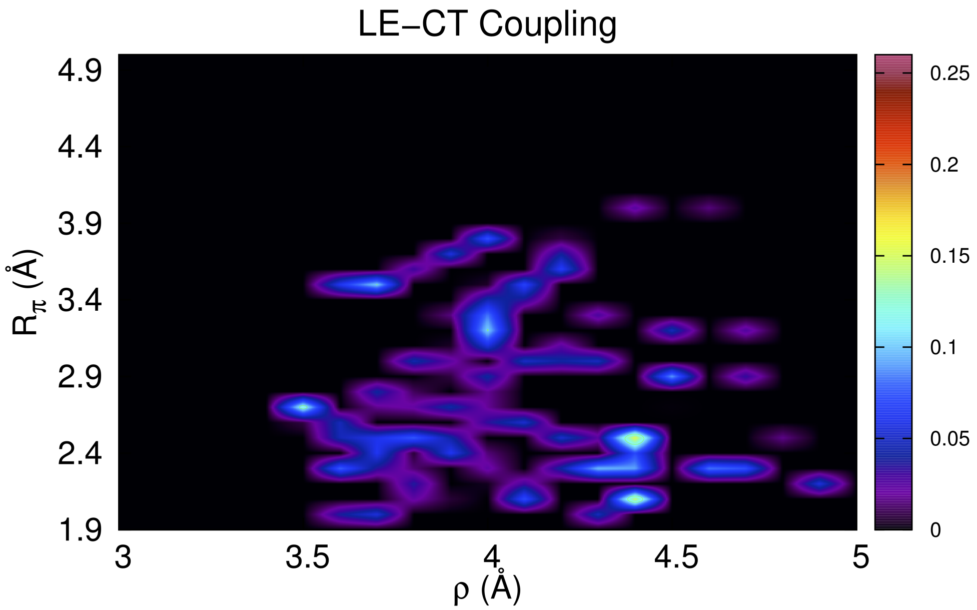

Figure S41: 2D heat map correlating the magnitude of the LE-LE coupling (colour palette in eV) with the values of ρ(Å) and R_π(Å) assumed from the dimer. Snapshots extracted from the *anti* MD trajectory.

S2.13 Diabatic energies and inter-state couplings QD statistics

Table D: Average values (left) and standard deviation (right), in eV, of the diabatic states energies (diagonal) and inter-state coupling (off-diagonal), for the set of *anti* (top) and *syn* (bottom) snapshots. The matrices are symmetric and only the upper half is reported. Absolute values were taken for each snapshot before the statistical analysis.

	Average (anti)				Standard deviation (anti)			
	L_1	L_2	CT(1→2)	CT(2→1)	L_1	L_2	CT(1→2)	CT(2→1)
L_1	2.72	0.11	0.03	0.04	0.02	0.02	0.02	0.03
L_2		2.72	0.04	0.03		0.02	0.03	0.02
CT(1→2)			2.91	0.01			0.16	0.00
CT(2→1)				2.94				0.16
	Average (syn)				Standard deviation (syn)			
	L_1	L_2	CT(1→2)	CT(2→1)	L_1	L_2	CT(1→2)	CT(2→1)
L_1	2.67	0.13	0.07	0.06	0.02	0.01	0.05	0.05
L_2		2.67	0.07	0.07		0.02	0.05	0.05
CT(1→2)			2.86	0.00			0.14	0.00
CT(2→1)				2.84				0.14

Table D reports the average values and standard deviations of the elements of the Hamiltonian matrix at position (0) taken over the snapshots extracted from *anti* and *syn* trajectories in acetonitrile. It clearly shows that for *syn* trajectory vertical energies are slightly smaller (with smaller standard deviation for CT states) and couplings slightly larger (with larger standard deviations).

Table E reports the values of the elements of the LVC Hamiltonian matrix at the reference position (0) for a specific snapshot in acetonitrile where the CT(1→2) and CT(2→1) populations? are respectively very large and vanishingly small. By repeating the computation of the matrix after removing all solvent molecules we clearly show the remarkable effect played by the specific solvent configuration. In gas phase in fact the two CT states are almost degenerate and less stable than the local excitations by ∼ 0.2 eV. On the contrary, in solution CT(1→2) and CT(2→1) are respectively stabilized and de-stabilized by ∼ 0.5 eV, so that the former becomes strongly more stable than the initial state of the dynamics L_1 while the latter is so higher in energy that cannot be populated.

Table E: Hamiltonian in the diabatic basis in solution (left) and in gas phase (right) for a specific snapshot of the PDI dimer in *anti* configuration on which the $|CT(1 \rightarrow 2)\rangle$ state acquires high population during the quantum dynamics. The matrices are symmetric and only the upper half is reported. The terms in the diagonal represent the vertical excitation energies. All values are in eV.

	Solution				Gas Phase			
	L_1	L_2	CT(1→2)	CT(2→1)	L_1	L_2	CT(1→2)	CT(2→1)
L_1	2.76	0.12	-0.04	0.04	2.75	0.12	0.04	-0.05
L_2		2.73	0.04	-0.04		2.72	-0.05	0.04
CT(1→2)			2.41	0.00			2.92	0.00
CT(2→1)				3.48				2.95

S2.14 Absorption spectrum and population dynamics predicted by the averaged Hamiltonian

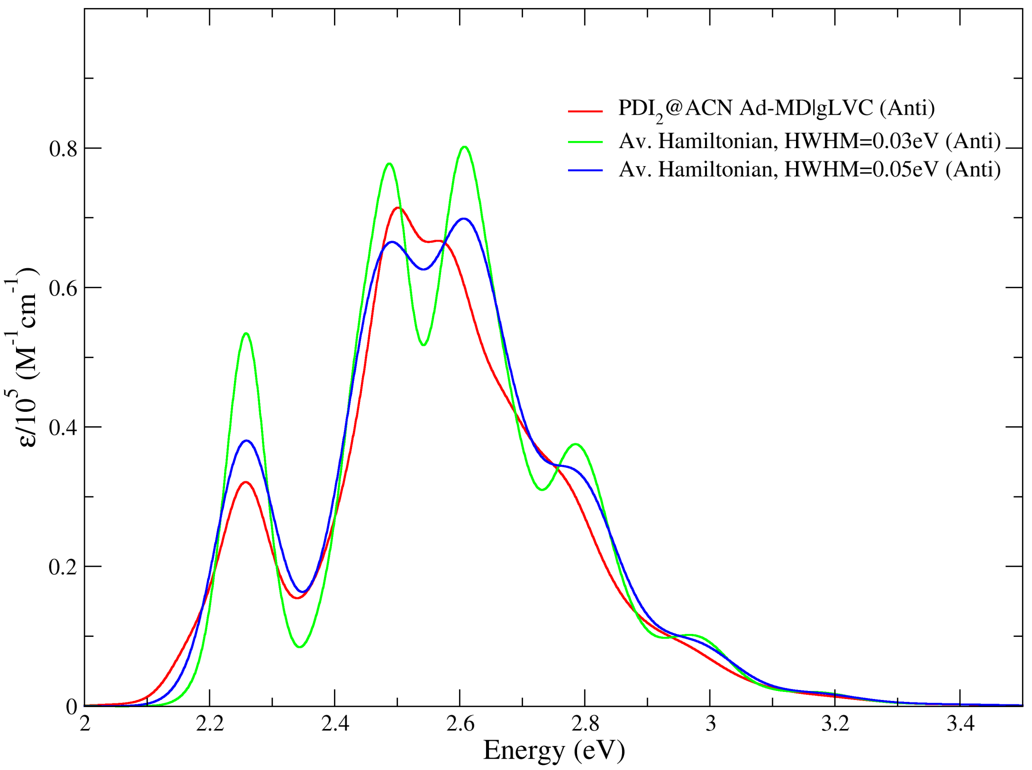

Figure S42: Calculated spectra obtained by averaging the individual spectra of each snapshot (red) and the spectra obtained from a wavepacket propagation under the effect of a single LVC Hamiltonian with values obtained by the average of $E^d_{ii}(0)$ and $E^d_{ij}(0)$ values over the set of 100 snapshots (green and blue). Notice that we also added the results with a larger broadening (blue) selected so try to phenomenologically account for the effect of the fluctuation of the parameters, which is lost in the average procedure. Average values taken from Table D, *anti* conformer PDI dimer in ACN.

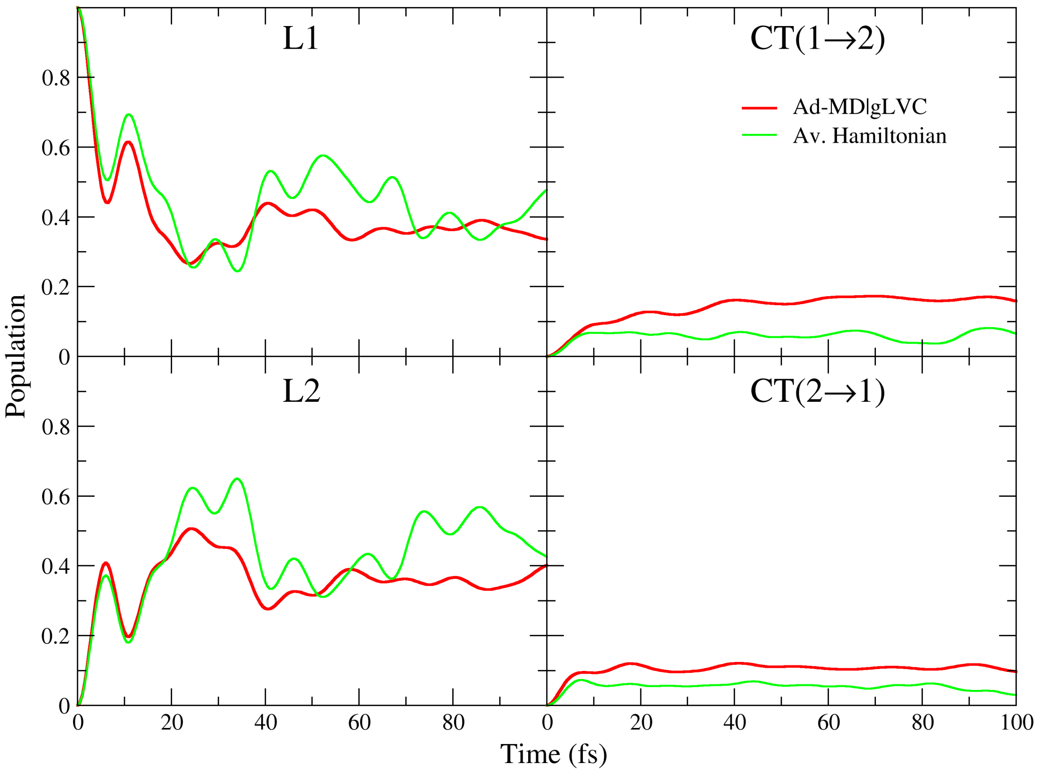

Figure S43: Population dynamics of the diabatic states after photoexcitation to $|L_1\rangle$ obtained by averaging the populations of 100 snapshots (red) or from a single propagation of a single LVC Hamiltonian with values obtained by the average of $E^d_{ii}(0)$ and $E^d_{ij}(0)$ values over the set of 100 snapshots (green). Average LVC values taken from Table D, *anti* conformer PDI dimer in ACN.

References

(S1) Cerezo, J.; Aranda, D.; Avila Ferrer, F. J.; Prampolini, G.; Santoro, F. Adiabatic-molecular dynamics generalized vertical hessian approach: a mixed quantum classical method to compute electronic spectra of flexible molecules in the condensed phase. *Journal of chemical theory and computation* **2019**, *16*, 1215–1231.

(S2) Tomasi, J.; Mennucci, B.; Cammi, R. Quantum Mechanical Continuum Solvation Models. *Chem. Rev.* **2005**, *105*, 2999–3094.

(S3) Segalina, A.; Cerezo, J.; Prampolini, G.; Santoro, F.; Pastore, M. Accounting for Vibronic Features through a Mixed Quantum-Classical Scheme: Structure, Dynamics, and Absorption Spectra of a Perylene Diimide Dye in Solution. *Journal of Chemical Theory and Computation* **2020**, *16*, 7061–7077.

(S4) Wang, L.-P.; Song, C. Geometry optimization made simple with translation and rotation coordinates. *J. Chem. Phys.* **2016**, *144*, 214108.

(S5) Cederbaum, L. S.; Gindensperger, E.; Burghardt, I. Short-Time Dynamics Through Conical Intersections in Macrosystems. *Phys. Rev. Lett.* **2005**, *94*, 113003.

(S6) Gindensperger, E.; Burghardt, I.; Cederbaum, L. S. Short-time dynamics through conical intersections in macrosystems. I. Theory: Effective-mode formulation. *The Journal of Chemical Physics* **2006**, *124*, 144103.

(S7) Gindensperger, E.; Burghardt, I.; Cederbaum, L. S. Short-time dynamics through conical intersections in macrosystems. II. Applications. *The Journal of Chemical Physics* **2006**, *124*, 144104.

(S8) Padula, D.; Picconi, D.; Lami, A.; Pescitelli, G.; Santoro, F. Electronic circular dichroism in exciton-coupled dimers: vibronic spectra from a general all-coordinates quantum-dynamical approach. *The Journal of Physical Chemistry A* **2013**, *117*, 3355–3368.

(S9) Picconi, D.; Lami, A.; Santoro, F. Hierarchical transformation of Hamiltonians with linear and quadratic couplings for nonadiabatic quantum dynamics: Application to the $\pi\pi^*/n\pi^*$ internal conversion in thymine. *The Journal of Chemical Physics* **2012**, *136*, 244104.

(S10) Avila Ferrer, F. J.; Santoro, F. Comparison of vertical and adiabatic harmonic approaches for the calculation of the vibrational structure of electronic spectra. *Phys. Chem. Chem. Phys.* **2012**, *14*, 13549–13563.

(S11) Iozzi, M. F.; Mennucci, B.; Tomasi, J.; Cammi, R. Excitation energy transfer (EET) between molecules in condensed matter: A novel application of the polarizable continuum model (PCM). *The Journal of Chemical Physics* **2004**, *120*, 7029–7040.
